# A Molecularly Cloned, Live-Attenuated Japanese Encephalitis Vaccine SA_14_-14-2 Virus: A Conserved Single Amino Acid in the *ij* Hairpin of the Viral E Glycoprotein Determines Neurovirulence in Mice

**DOI:** 10.1371/journal.ppat.1004290

**Published:** 2014-07-31

**Authors:** Sang-Im Yun, Byung-Hak Song, Jin-Kyoung Kim, Gil-Nam Yun, Eun-Young Lee, Long Li, Richard J. Kuhn, Michael G. Rossmann, John D. Morrey, Young-Min Lee

**Affiliations:** 1 Department of Animal, Dairy, and Veterinary Sciences; Utah Science Technology and Research, College of Agriculture and Applied Sciences, Utah State University, Logan, Utah, United States of America; 2 Department of Microbiology, College of Medicine, Chungbuk National University, Cheongju, Republic of Korea; 3 Department of Anatomy, College of Medicine, Chungbuk National University, Cheongju, Republic of Korea; 4 Department of Biological Sciences, Purdue University, West Lafayette, Indiana, United States of America; Saint Louis University, United States of America

## Abstract

Japanese encephalitis virus (JEV), a mosquito-borne flavivirus that causes fatal neurological disease in humans, is one of the most important emerging pathogens of public health significance. JEV represents the JE serogroup, which also includes West Nile, Murray Valley encephalitis, and St. Louis encephalitis viruses. Within this serogroup, JEV is a vaccine-preventable pathogen, but the molecular basis of its neurovirulence remains unknown. Here, we constructed an infectious cDNA of the most widely used live-attenuated JE vaccine, SA_14_-14-2, and rescued from the cDNA a molecularly cloned virus, SA_14_-14-2^MCV^, which displayed *in vitro* growth properties and *in vivo* attenuation phenotypes identical to those of its parent, SA_14_-14-2. To elucidate the molecular mechanism of neurovirulence, we selected three independent, highly neurovirulent variants (LD_50_, <1.5 PFU) from SA_14_-14-2^MCV^ (LD_50_, >1.5×10^5^ PFU) by serial intracerebral passage in mice. Complete genome sequence comparison revealed a total of eight point mutations, with a common single G^1708^→A substitution replacing a Gly with Glu at position 244 of the viral E glycoprotein. Using our infectious SA_14_-14-2 cDNA technology, we showed that this single Gly-to-Glu change at E-244 is sufficient to confer lethal neurovirulence in mice, including rapid development of viral spread and tissue inflammation in the central nervous system. Comprehensive site-directed mutagenesis of E-244, coupled with homology-based structure modeling, demonstrated a novel essential regulatory role in JEV neurovirulence for E-244, within the *ij* hairpin of the E dimerization domain. In both mouse and human neuronal cells, we further showed that the E-244 mutation altered JEV infectivity *in vitro*, in direct correlation with the level of neurovirulence *in vivo*, but had no significant impact on viral RNA replication. Our results provide a crucial step toward developing novel therapeutic and preventive strategies against JEV and possibly other encephalitic flaviviruses.

## Author Summary

A group of mosquito-borne flaviviruses that cause fatal encephalitis in humans is among the most important of all emerging human pathogens of global significance. This group includes Japanese encephalitis (JE), West Nile, St. Louis encephalitis, and Murray Valley encephalitis viruses. In this work, we have developed a reverse genetics system for SA_14_-14-2, a live JE vaccine that is most commonly used in JE-endemic areas, by constructing an infectious bacterial artificial chromosome that contains the full-length SA_14_-14-2 cDNA. Using this infectious SA_14_-14-2 cDNA, combined with a mouse model for JEV infection, we have identified a key viral neurovirulence factor, a conserved single amino acid in the *ij* hairpin adjacent to the fusion loop of the viral E glycoprotein, which regulates viral infectivity into neurons within the central nervous system *in vivo* and neuronal cells of mouse and human *in vitro*. Thus, our findings elucidate the molecular basis of the neurovirulence caused by JEV and other closely related encephalitic flaviviruses, a major step in understanding their neuropathogenesis. From a clinical perspective, the discovery of the viral neurovirulence factor and its role will have direct application to the design of a novel class of broad-spectrum antivirals to treat and prevent infection of JEV and other taxonomically related neurotropic flaviviruses.

## Introduction

Japanese encephalitis virus (JEV) is the most common cause of viral encephalitis in Asia and parts of the Western Pacific, with ∼60% of the world's population at risk of infection [1]. Within the family *Flaviviridae* (genus *Flavivirus*), JEV belongs to the JE serological group, which also includes medically important human pathogens found on every continent except Antarctica [2], [3]: West Nile virus (WNV), St. Louis encephalitis virus (SLEV), and Murray Valley encephalitis virus (MVEV). Historically, the JE serological group members have clustered in geographically distinct locations, but the recent emergence and spread of JEV in Australia [4] and WNV in North America [5], [6] have caused growing concern that these viruses can spread into new territory, posing a significant challenge for global public health [3], [7]. In the US, where WNV and SLEV are endemic, the situation is particularly problematic because the likelihood of JEV being introduced is considerable [8], [9]. Worldwide, ∼50,000–175,000 clinical cases of JE are estimated to occur annually [10]; however, this incidence is undoubtedly a considerable underestimate because surveillance and reporting are inadequate in most endemic areas, and only ∼0.1–4% of JEV-infected people develop clinical disease [11], [12]. On average, ∼20–30% of patients die, and ∼30–50% of survivors suffer from irreversible neurological and/or psychiatric sequelae [13]. Most clinical cases occur in children under age 15 in endemic areas, but in newly invaded areas, all age groups are affected because protective immunity is absent [14]. Thus, given the current disease burden and significant threat of the JEV emergence, resurgence, and spread among much larger groups of susceptible populations, control of JEV remains a high public health priority.

JEV contains a nucleocapsid composed of an ∼11-kb plus-strand genomic RNA, complexed with multiple copies of the highly-basic α-helical C proteins [15], [16]. The nucleocapsid is surrounded by a host-derived lipid bilayer containing the membrane-anchored M and E proteins [17]–[19]. The initial step in the flavivirus replication cycle involves attachment of the virions to the surface of susceptible cells [20]–[24]. The viral E protein is then assumed to bind with high affinity and specificity to an as-yet unidentified cellular receptor(s), which triggers receptor-mediated, clathrin-dependent endocytosis [25]–[27]. The acidic conditions in the endosome lead to a conformational change in the E protein [28]–[32], which triggers fusion of the viral membrane with host endosomal membrane [33]. Once the genome is released into the cytoplasm, the genomic RNA is translated into a single polyprotein, which is processed co- and post-translationally by host and viral proteases to yield at least 10 functional proteins [34]: three structural (C, prM, and E) and seven nonstructural (NS1, NS2A, NS2B, NS3, NS4A, NS4B, and NS5). The nonstructural proteins actively replicate the viral genomic RNA in the replication complex [35]–[38] that is associated with the virus-induced, ER-derived membranes [39]–[41]. Newly synthesized genomic RNA and C proteins are initially enveloped by the prM and E proteins to generate immature virions [42], [43] that bud into the lumen of the ER [44]. These immature virions are then transported via the secretory pathway to the Golgi apparatus. In the low-pH environment of the *trans*-Golgi network, the furin-mediated cleavage of prM to M induces the maturation of the viral particles [45], which is also accompanied by significant structural rearrangements of the M and E proteins [42], [46], [47]. Finally, mature virions are released into the extracellular space by exocytosis.

JEV is maintained in an enzootic cycle involving multiple species of mosquito vectors (primarily *Culex* species) and vertebrate hosts/reservoirs (mainly domestic pigs/wading birds). Humans become infected incidentally when bitten by an infected mosquito [48]. In the absence of antiviral therapy, active immunization is the only strategy for sustainable long-term protection. Four types of JE vaccines are used in different parts of the world [49], [50]: (*i*) the mouse brain-derived inactivated vaccine based on the Nakayama or Beijing-1 strain, (*ii*) the cell culture-derived inactivated vaccine based on the Beijing-3 or SA_14_-14-2 strain, (*iii*) the cell culture-derived live-attenuated vaccine based on the SA_14_-14-2 strain, and (*iv*) the live chimeric vaccine developed on a yellow fever virus (YFV) 17D genetic background that carries two surface proteins of JEV SA_14_-14-2. Of the four vaccines, the only one that is available internationally is the mouse brain-derived inactivated Nakayama [11]. Unfortunately, the production of this vaccine was discontinued in 2006 [51] because of vaccine-related adverse events, short-term immunity, and high production cost [13], [52]. To date, the most commonly used vaccine in Asia is the live-attenuated SA_14_-14-2 [53], but this vaccine is not recommended by the WHO for global immunization [13], [54]. In addition to the dependence of the duration of immunity on the number of doses received, there is at least a theoretical risk of virus mutation and reversion of the vaccine virus to high virulence. Recently, the SA_14_-14-2 vaccine virus has been utilized to produce a new Vero cell-derived inactivated vaccine that has been approved in the US, Europe, Canada, and Australia since 2009 [51], [55], [56]. In the US, this vaccine is recommended for adults aged ≥17 years travelling to JEV-endemic countries and at risk of JEV exposure [51], [57], but no vaccine is currently available for children under 17 [58]. More recently, the prM and E genes of JEV SA_14_-14-2 have been used to replace the corresponding genes of YFV 17D [59], creating a live chimeric vaccine [60] that is now licensed in Australia and Thailand [61], [62]. Thus, the application of JEV SA_14_-14-2 to vaccine development and production is continuously expanding, but the viral factors and fundamental mechanisms responsible for its loss of virulence are still elusive.

The virulence of JEV is defined by two properties: (*i*) neuroinvasiveness, the ability of the virus to enter the central nervous system (CNS) when inoculated by a peripheral route; and (*ii*) neurovirulence, the ability of the virus to replicate and cause damage within the CNS when inoculated directly into the brain of a host. Over the past 20 years, many investigators have sought to understand the molecular basis of JEV virulence, by using cell and animal infection model systems to compare the nucleotide sequences of the genomes of several JEV strains that differ in virological properties [63]–[74]. These studies have identified a large number of mutations scattered essentially throughout the entire viral genome. Because of the complexity of the mutations, however, the major genetic determinant(s) critical for either JEV neurovirulence or neuroinvasiveness remains unclear. In particular, the situation is more complicated for the live-attenuated SA_14_-14-2 virus, which has been reported to have a number of different mutations, i.e., 47–64 nucleotide changes (17–27 amino acid substitutions), when compared to its virulent parental strain SA_14_; the exact number depends on both the passage history of the viruses and the type of cell substrate used for virus cultivation [63]–[65]. A more comprehensive sequence comparison with another SA_14_-derived attenuated vaccine strain, SA_14_-2-8, together with two other virulent strains, has suggested seven common amino acid substitutions that may be involved in virus attenuation: 4 in E, 1 in NS2B, 1 in NS3, and 1 in NS4B [64]. However, the genetic component directly responsible for the attenuation of SA_14_-14-2 is still unknown. Given that SA_14_-14-2 has been administered to >300 million children for >20 years in China and recently in other Asian countries [53], it is striking that there is a fundamental gap in our knowledge at the molecular level about how SA_14_-14-2 is attenuated.

Here we report the development of an infectious cDNA-based reverse genetics system for JEV SA_14_-14-2 that has enabled the analysis of molecular aspects of its attenuation in neurovirulence. By *in vivo* passage of a molecularly defined, cDNA-derived SA_14_-14-2 virus, we generated three isogenic variants, each displaying lethal neurovirulence in mice, with a common single G^1708^→A substitution that corresponds to a Gly→Glu change at position 244 of the viral E glycoprotein. By *in vitro* site-directed mutagenesis of the infectious SA_14_-14-2 cDNA, coupled with conventional virologic and experimental pathologic methods and homology-based structure modeling, we have demonstrated a novel regulatory role in JEV neurovirulence of a conserved single amino acid at position E-244 in the *ij* hairpin adjacent to the fusion loop of the E dimerization domain. These findings offer new insights into the molecular mechanism of JEV neurovirulence and will directly aid the development of new approaches to treating and preventing JEV infection.

## Results

### Development of a full-length infectious cDNA of SA_14_-14-2, a live JE vaccine virus

**Figure 1:**
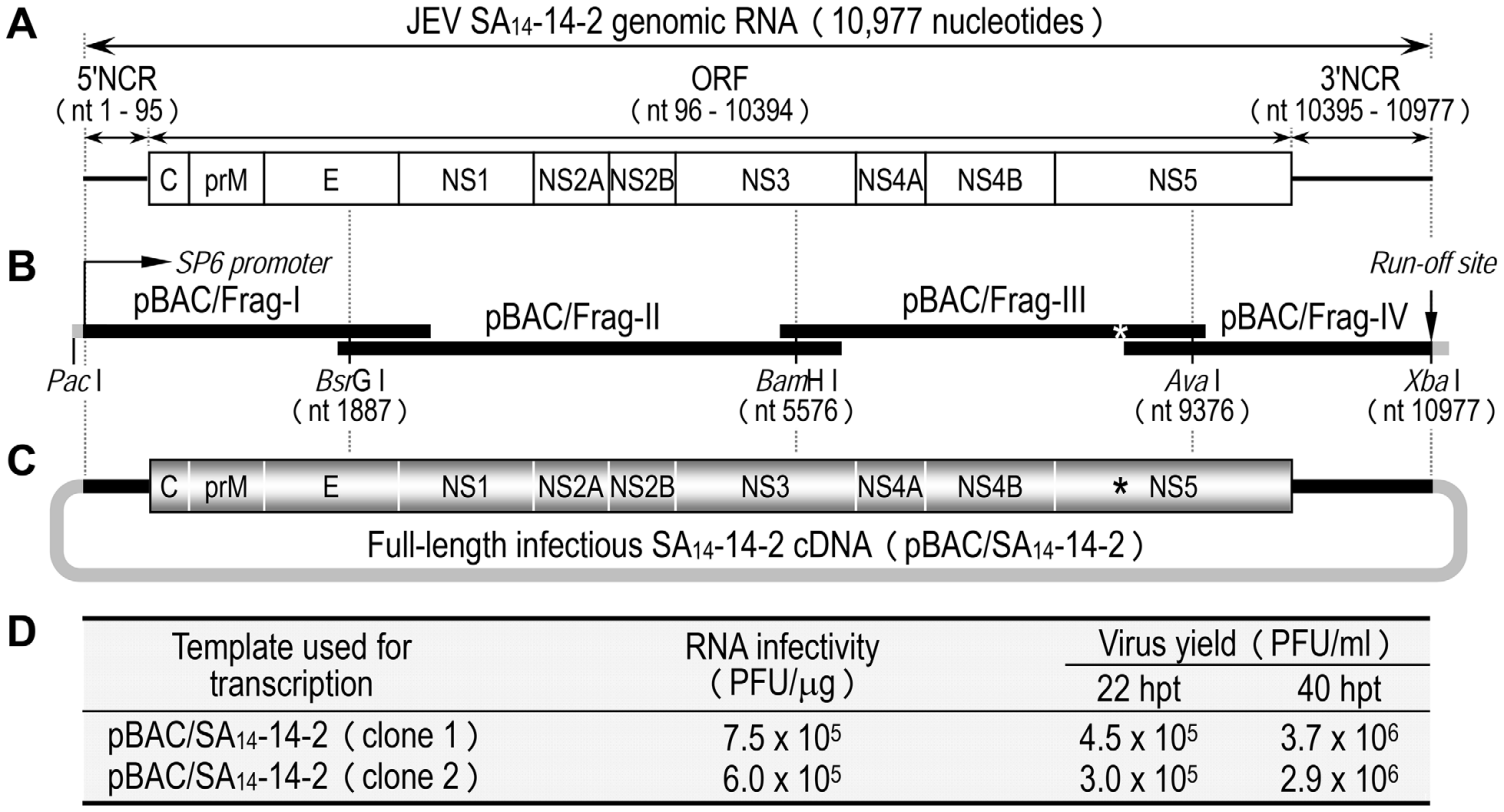
Construction of the full-length infectious SA_14_-14-2 cDNA as a BAC. (**A**) Structure of the SA_14_-14-2 genomic RNA (GenBank accession no. JN604986). NCR, non-coding region; ORF, open reading frame. (**B**) Diagram of a panel of four overlapping SA_14_-14-2 cDNAs contained in pBAC/Frag-I to IV. SP6 promoter and an artificial run-off site are shown. An asterisk indicates a pre-existing *Xba*I site at nucleotide 9131 that was inactivated by introducing a silent point mutation, A^9134^→T. (**C**) Structure of the full-length SA_14_-14-2 cDNA (pBAC/SA_14_-14-2). (**D**) Functionality of pBAC/SA_14_-14-2. After *in vitro* transcription with SP6 RNA polymerase, RNA transcripts were electroporated into BHK-21 cells, and infectious plaque centers were determined (RNA infectivity). At 22 and 40 hpt, supernatants from RNA-transfected cells were harvested for virus titration on BHK-21 cells (Virus yield).

As an initial step in investigating the molecular basis for the virulence attenuation of SA_14_-14-2, we generated a full-length infectious SA_14_-14-2 cDNA to serve as a template for genetic manipulation of the viral genome (Fig. 1A). The 10,977-nucleotide genome of SA_14_-14-2 (GenBank accession number JN604986) was first cloned as four contiguous cDNAs into the bacterial artificial chromosome (BAC) designated pBAC/Frag-I to IV (Fig. 1B). pBAC/Frag-I was modified to have an SP6 promoter immediately upstream of the viral 5′-end, and pBAC/Frag-IV was engineered to contain an artificial *Xba*I run-off site just downstream of the viral 3′-end, allowing *in vitro* run-off transcription of capped, genome-length RNAs bearing authentic 5′ and 3′ ends of the genomic RNA. Since the viral genome already had an internal *Xba*I site at nucleotide 9131 in the NS5 protein-coding region, this pre-existing site was eliminated in pBAC/Frag-III by introducing a silent point mutation, A^9134^→T (Fig. 1B, asterisk), which in turn served as a genetic marker to identify the cDNA-derived virus. In the last cloning step, a panel of the four overlapping SA_14_-14-2 cDNAs was sequentially assembled by joining at three natural restriction sites (*Bsr*GI, *Bam*HI, and *Ava*I) to create the full-length SA_14_-14-2 cDNA, pBAC/SA_14_-14-2 (Fig. 1C).

The functionality of pBAC/SA_14_-14-2 was analyzed by determining the specific infectivity of the synthetic RNAs transcribed *in vitro* from the cDNA after RNA transfection into susceptible BHK-21 cells (Fig. 1D). Two independent clones of pBAC/SA_14_-14-2 were linearized by *Xba*I, followed by mung bean nuclease treatment to remove the 5′ overhang left by the *Xba*I digestion. Each was then used as a template for SP6 polymerase run-off transcription in the presence of the m^7^G(5′)ppp(5′)A cap structure analog. Transfection of the synthetic RNAs into BHK-21 cells gave specific infectivities of 6.0–7.5×10^5^ PFU/µg; the virus titers recovered from the RNA-transfected cells were 3.0–4.5×10^5^ PFU/ml at 22 h post-transfection (hpt) and increased ∼10-fold to 2.9–3.7×10^6^ PFU/ml at 40 hpt (Fig. 1D). Unequivocally, the recovered virus contained the marker mutation (A^9134^→T) that had been introduced in pBAC/SA_14_-14-2 (data not shown). Our results show that the synthetic RNAs generated from the full-length SA_14_-14-2 cDNA are highly infectious in BHK-21 cells, producing a high titer of molecularly defined, infectious virus.

**Figure 2:**
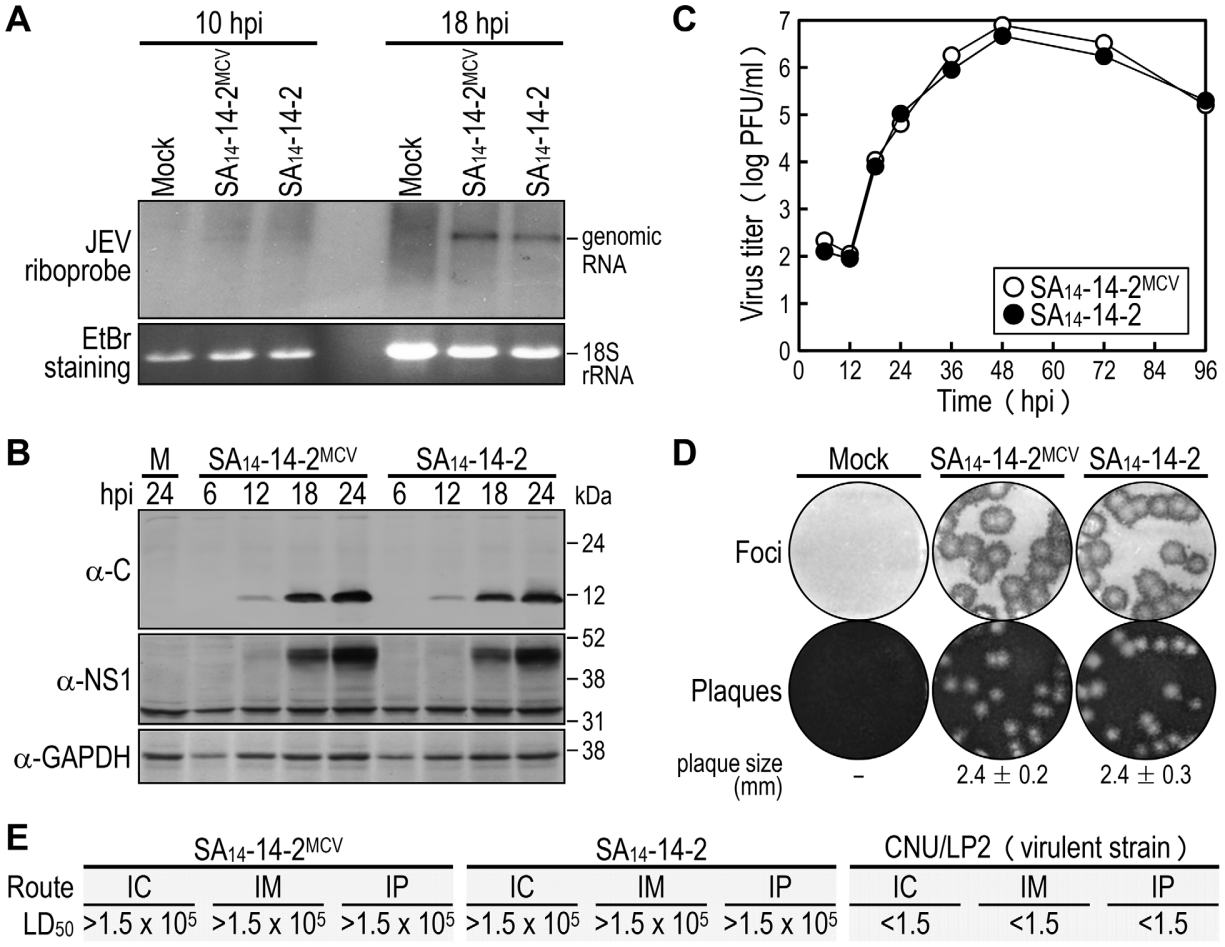
Characterization of biological properties of the molecularly cloned virus SA_14_-14-2^MCV^ *in vitro* and *in vivo*. (**A**-**D**) BHK-21 cells were mock-infected or infected at an MOI of 1 with the molecularly cloned virus (SA_14_-14-2^MCV^) or the original parental virus (SA_14_-14-2). At the time points indicated, cells were lysed to analyze the accumulation levels of viral genomic RNA by Northern blotting (**A**) and viral proteins by immunoblotting (**B**), and culture supernatants were harvested to examine the production levels of progeny virions by plaque titration on BHK-21 cells (**C**). At 4 dpi, cell monolayers were first immunostained with a mouse α-JEV antiserum to visualize the infectious foci, and the same monolayers were then restained with crystal violet to observe the infectious plaques (**D**). The average plaque sizes (mean ± SD) were estimated by counting 10 representative plaques. (**E**) Groups of 3-week-old ICR mice (*n* = 20 per group) were infected IC, IM, or IP with serial 10-fold dilutions of each virus as indicated. The LD_50_ values (in PFU) were calculated by the Reed and Muench method [119]. CNU/LP2, a virulent JEV strain used as a reference.

In cell cultures [75], [76], we assessed the *in vitro* growth properties of the molecularly cloned virus (SA_14_-14-2^MCV^) rescued from the infectious cDNA, as compared to those of the uncloned parental virus (SA_14_-14-2) used for cDNA construction. In hamster kidney BHK-21 cells, which are used most frequently for JEV propagation in laboratories, SA_14_-14-2^MCV^ replicated as efficiently as SA_14_-14-2, with no noticeable difference in the accumulation of viral genomic RNA (Fig. 2A) and proteins (Fig. 2B) over the first 24 h after infection at a multiplicity of infection (MOI) of 1 plaque-forming unit (PFU) per cell. These observations were consistent with their growth kinetics, which were essentially identical for 4 days following infection at three different MOIs: 0.1, 1, and 10 PFU/cell (Fig. 2C and data not shown). Similarly, there was no difference in focus/plaque morphology between SA_14_-14-2^MCV^ and SA_14_-14-2 at 4 days post-infection (dpi) (Fig. 2D); as expected, their foci/plaques were ∼30% smaller than those produced by CNU/LP2, a virulent JEV strain used as a reference (Fig. S1). Also, their growth properties were equivalent in two other cell lines, human neuroblastoma SH-SY5Y and mosquito C6/36 cells, which are potentially relevant to JEV pathogenesis and transmission, respectively (Fig. S2). These data suggest that the uncloned parental and molecularly cloned viruses are indistinguishable in viral replication and spread in both mammalian and insect cells.

In mice [75], [77], we evaluated *in vivo* the attenuation phenotypes of SA_14_-14-2^MCV^ and SA_14_-14-2, with a virulent JEV CNU/LP2 [78] in parallel. Groups of 3-week-old ICR mice (*n* = 20) were infected with various doses (1.5 to 1.5×10^5^ PFU/mouse) of each virus, via three different inoculation routes: intracerebral (IC) for neurovirulence, and intramuscular (IM) and intraperitoneal (IP) for neuroinvasiveness. As with SA_14_-14-2, the 50% lethal doses (LD_50_s) of SA_14_-14-2^MCV^, regardless of the route of inoculation, were all >1.5×10^5^ PFU (Figs. 2E and S3). Specifically, all mice infected with SA_14_-14-2^MCV^ or SA_14_-14-2 remained healthy and displayed no clinical signs of JEV infection (e.g., ruffled fur, hunched posture, tremors, or hindlimb paralysis) after IM or IP inoculation with any of the tested doses; on the other hand, a small fraction of the mice infected with SA_14_-14-2^MCV^ (5–20%) or SA_14_-14-2 (5–10%) developed typical symptoms and death after the IC inoculation with a relatively high dose of ≥1.5×10^3^ PFU/mouse, but not the low dose of ≤1.5×10^2^ PFU/mouse (Fig. S3). In all dead or surviving mice, virus titration confirmed the presence (1.8–4.1×10^6^ PFU/brain) or absence, respectively, of viral replication in their brain tissues. As expected [75], [77], the LD_50_ values of CNU/LP2 [78], irrespective of the inoculation route, were always <1.5 PFU (Figs. 2E and S3); the control groups of mock-infected mice all survived with no signs of disease (Fig. S3). Thus, our data indicate that SA_14_-14-2^MCV^ displays a variety of biological properties identical to those of SA_14_-14-2, both *in vitro* and *in vivo*.

### Generation of three highly neurovirulent variants derived from SA_14_-14-2^MCV^

**Figure 3:**
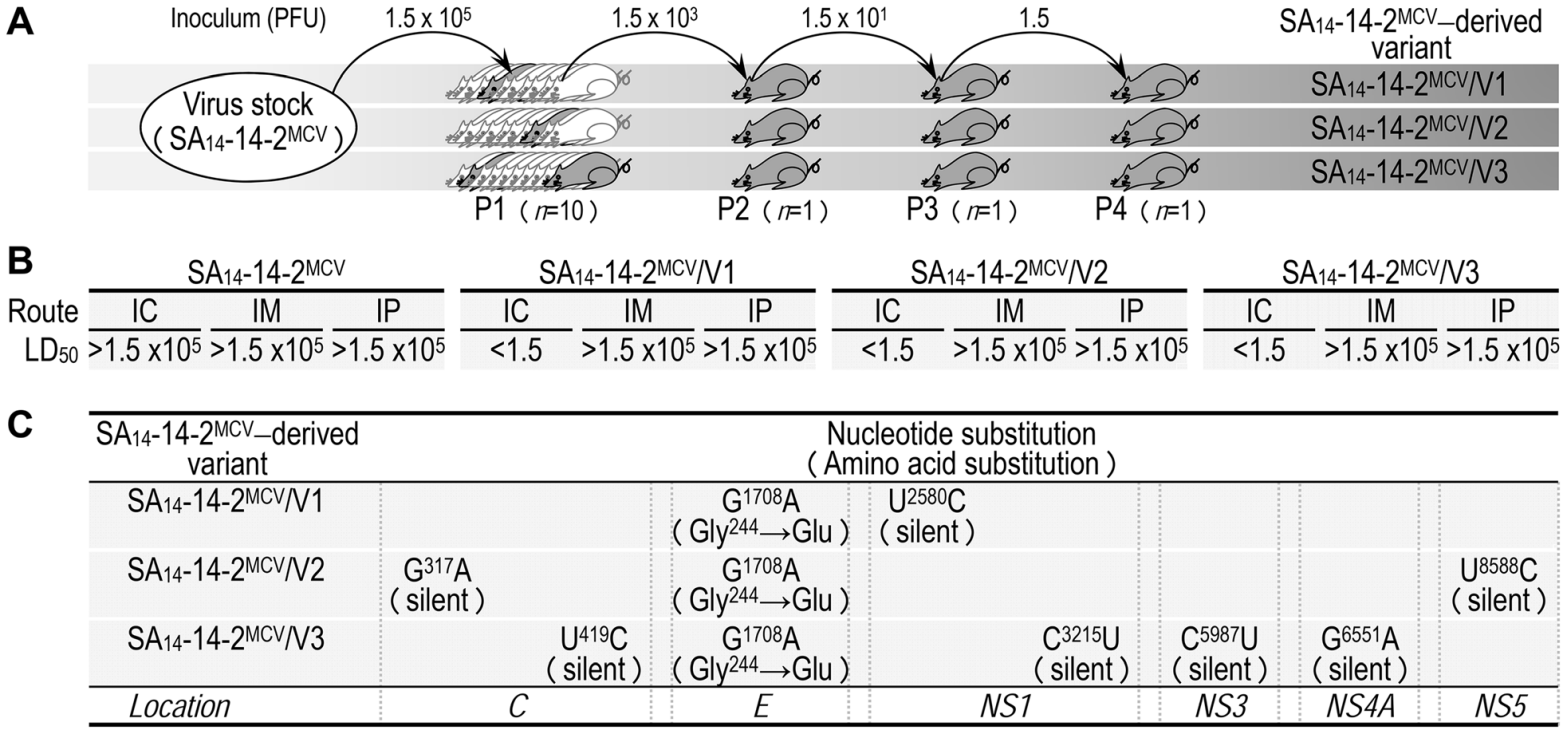
Development of three independent, highly neurovirulent variants from SA_14_-14-2^MCV^ by serial intracerebral passage in mice. (**A**) Diagram illustrating the *in vivo* passage of SA_14_-14-2^MCV^. (**B**) Virological properties of three SA_14_-14-2^MCV^ variants in mice. Groups of 3-week-old ICR mice (*n* = 10 per group) were inoculated IC, IM, or IP with doses of the virus stock serially diluted 10-fold. The LD_50_ values were determined in PFU. (**C**) Comparison of the complete genome sequence of the SA_14_-14-2^MCV^ parental virus and its three variant viruses.

As was true for SA_14_-14-2, direct inoculation of a relatively high dose of SA_14_-14-2^MCV^ into mouse brains initiated a productive infection in the CNS and caused lethal encephalitis, albeit at a very low frequency (Fig. S3). Intrigued by this observation, we decided to generate isogenic neurovirulent variants from SA_14_-14-2^MCV^ by serial brain-to-brain passage in mice (Fig. 3A). At passage 1 (P1), the cDNA-derived SA_14_-14-2^MCV^ was directly inoculated into the brains of 3-week-old ICR mice at 1.5×10^5^ PFU/mouse (three groups, *n* = 10 per group); one or two infected mice per group exhibited clinical symptoms of JEV infection. At the onset of hindlimb paralysis (6–10 dpi), virus was harvested from the brain of a moribund mouse in each group (3 total); in each case, a brain homogenate was prepared for plaque titration and used as an inoculum for the next round of passage. Serial intracerebral passage was continued for three additional rounds, with a gradually decreasing inoculum in order to ensure the stability of selected mutations and a sufficiently pure population of viruses: 1,500 (P2), to 15 (P3), to 1.5 PFU/mouse (P4). Using this approach, we obtained three independently selected variants, SA_14_-14-2^MCV^/V1 to V3 (Fig. 3A).

We first compared the biological properties of the three SA_14_-14-2^MCV^ variants, both *in vitro* and *in vivo*, to those of the parental SA_14_-14-2^MCV^. In three cell cultures (BHK-21, SH-SY5Y, and C6/36), all three variants exhibited characteristics of viral replication identical to the parent, as demonstrated by (*i*) quantitative real-time RT-PCRs to measure the level of viral genomic RNA production, (*ii*) immunoblotting with a panel of JEV-specific rabbit polyclonal antisera to probe the profile and level of viral structural and nonstructural protein accumulation, and (*iii*) one-step growth analyses to assess the yield of progeny virions produced during a single round of infection (data not shown). In 3-week-old ICR mice, however, there was a clear difference between the parent and the three variants in both phenotype and virulence level (Fig. 3B). When peripherally inoculated (i.e., IM and IP), neither the parent nor its three variants caused any symptoms or death at a maximum dose of 1.5×10^5^ PFU/mouse. In contrast, when inoculated IC, the three variants, unlike the parent (IC LD_50_, >1.5×10^5^ PFU), were all highly neurovirulent (IC LD_50_s, <1.5 PFU) (Fig. 3B and Table S1). Our findings show that all three variants still lacked a detectable level of neuroinvasiveness but gained a high level of neurovirulence after serial IC passage in mice.

Next, we determined the complete nucleotide sequence of the genome of the three SA_14_-14-2^MCV^ variants to identify the nucleotide(s) and/or amino acid(s) in specific viral loci/genes that is(are) potentially responsible for the drastic increase in neurovirulence. According to our protocol [77], the consensus genome sequence of each variant was generated by direct sequencing of three overlapping, uncloned cDNA amplicons covering the entire viral RNA genome except the 5′- and 3′-termini; the remaining consensus sequences of the 5′- and 3′-terminal regions were obtained by 5′- and 3′-RACE reactions, each followed by cDNA cloning and sequencing of 10–15 independent clones. In all three variants, when the consensus genome sequence was compared to that of the parent, a single nucleotide G-to-A transition was always found at nucleotide 1708, changing a Gly (GGG) to Glu (GAG) codon at amino acid 244 of the viral E glycoprotein (Fig. 3C). In addition, each of the three variants also contained a small number of unique silent point mutations scattered over the genome, confirming they were indeed independent variants (Fig. 3C): one in SA_14_-14-2^MCV^/V1 (U^2580^C), two in SA_14_-14-2^MCV^/V2 (G^317^A and U^8588^C), and four in SA_14_-14-2^MCV^/V3 (U^419^C, C^3215^U, C^5987^U, and G^6551^A). These results suggest that the G^1708^A substitution, the only mutation observed in all three variants, may contribute to the viral neurovirulence in mice.

### Identification of a single Gly-to-Glu change at position E-244 that is responsible for the reversion to neurovirulence

**Figure 4:**
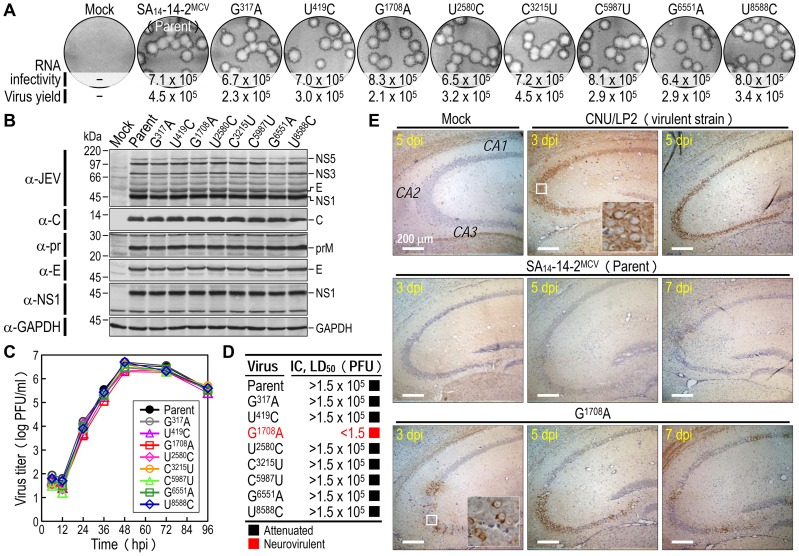
Discovery of a single locus that leads to the reversion of SA_14_-14-2^MCV^ to lethal neurovirulence. (**A**-**C**) *In vitro* replicability. BHK-21 cells were mock-transfected or transfected with RNAs transcribed from the parent or each mutant cDNA, as indicated. RNA infectivity (in PFU/µg) at 4 dpt was determined by infectious center assay, combined with staining of cell monolayers using an α-JEV antiserum, and virus yield (in PFU/ml) at 22 hpt was measured by plaque titration on BHK-21 cells (**A**); viral protein accumulation at 18 hpt was examined by immunoblotting of cell lysates with a panel of antibodies as indicated (**B**). For viral growth analysis, BHK-21 cells were infected at an MOI of 0.1 with the parent or each mutant virus obtained from the corresponding RNA-transfected cells. At the indicated time points, culture supernatants harvested for virus titration on BHK-21 cells (**C**). (**D**-**E**) *In vivo* neurovirulence. Groups of 3-week-old ICR mice (*n* = 10 per group) were inoculated IC with doses of the virus stock serially diluted 10-fold, and the LD_50_ values were determined (**D**). For immunohistochemical staining, groups of the mice (*n* = 15 per group) were mock-infected or infected IC with 10^3^ PFU of each virus. At 3, 5, and 7 dpi, five mice were processed for brain section staining with an α-NS1 antiserum. Shown are representative hippocampal slides (**E**).

To identify a key point mutation(s) in three variants of SA_14_-14-2^MCV^ that leads to the acquisition of neurovirulence, we generated eight derivatives of SA_14_-14-2^MCV^, each containing one of the eight point mutations found in our three variants, by cloning them individually into the infectious SA_14_-14-2 cDNA and transfecting the synthetic RNAs derived from each mutant cDNA into BHK-21 cells. In all cases, the mutant RNA was as infectious as the parent RNA, with a specific infectivity of 6.4–8.3×10^5^ PFU/µg; the sizes of the foci/plaques produced by each mutant RNA were indistinguishable from those generated by the parent RNA, paralleling their levels of virus production, with an average yield of 2.1–4.5×10^5^ PFU/ml at 22 hpt (Fig. 4A). In agreement with these results, no difference was observed in the profile or expression level of the viral proteins, i.e., three structural (C, prM, and E) and one nonstructural (NS1), as determined by immunoblotting of RNA-transfected cells at 18 hpt (Fig. 4B). All the mutant viruses grew as efficiently as did the parental virus over the course of 96 h after infection at an MOI of 0.1 in BHK-21 cells (Fig. 4C). Thus, there was no apparent effect of any of the eight introduced genetic changes on virus replication.

In mice, we examined the neurovirulence of these eight mutant viruses. Groups of 3-week-old ICR mice (*n* = 10 per group) were infected by IC inoculation with various doses (1.5 to 1.5×10^5^ PFU/mouse) of the parent or each mutant virus. One of the eight mutants containing the G^1708^A substitution had an IC LD_50_ of <1.5 PFU, making it capable of killing all mice within ∼7 dpi with a minimum dose of 1.5 PFU/mouse; the other seven mutants had IC LD_50_ values all >1.5×10^5^ PFU and behaved like the parental virus, with only <20% of infected mice developing clinical symptoms and death at a maximum dose of 1.5×10^5^ PFU/mouse (Fig. 4D and Table S2). In all dead or surviving mice, virus titration confirmed the presence (1.4–3.5×10^6^ PFU/brain) or absence, respectively, of productive viral replication in the brain tissues; as expected, all mock-infected mice survived with no signs of disease (data not shown). Thus, our findings showed that of the eight point mutations, a single G^1708^A substitution, replacing a Gly with Glu at amino acid residue 244 of the viral E glycoprotein, is sufficient to confer lethal neurovirulence in mice.

To determine whether the mutant G^1708^A, unlike the parent SA_14_-14-2^MCV^, is able to replicate and spread in the CNS, we immunohistochemically stained for JEV NS1 antigen in mouse brains after IC inoculation (Fig. 4E shows hippocampal slides, and Fig. S4 presents slides of other brain areas, i.e., amygdala, cerebral cortex, thalamus, hypothalamus, and brainstem): (*i*) In brains infected with a virulent JEV CNU/LP2 (control) [75], [77], [78], a large number of NS1-positive neurons were observed at 3 dpi in all areas we stained; this number was increased significantly at 5 dpi. In the hippocampus, most infected neurons were found in the CA2/3 region at 3 dpi and had spread to the CA1 region by 5 dpi. (*ii*) In brains infected with the parent SA_14_-14-2^MCV^, almost no NS1-positive cells were found in any brain region during the entire 7-day course of the experiment. In a few atypical cases, a small number of NS1-positive neurons were noted at 5–7 dpi in the hippocampal CA2/3 region, but not the CA1 region (data not shown). (*iii*) In brains infected with the mutant G^1708^A, a considerable number of NS1-positive neurons were observed at 3 dpi, mainly in the hippocampal CA2/3 region, and only a few in other areas (amygdala, cerebral cortex, thalamus, and brainstem); overall, the number of infected neurons was much lower than in brains infected with JEV CNU/LP2. At 5–7 dpi, the number of NS1-positive neurons was noticeably increased in the hippocampus (now in the CA1) and amygdala, but not in other brain regions. Our findings show that, in mice, a single G^1708^A substitution changing a Gly with Glu at position E-244 promotes susceptibility to SA_14_-14-2^MCV^ infection of neurons.

### Understanding the novel regulatory role in neurovirulence of E-244, located in the *ij* hairpin of the viral E glycoprotein

**Figure 5:**
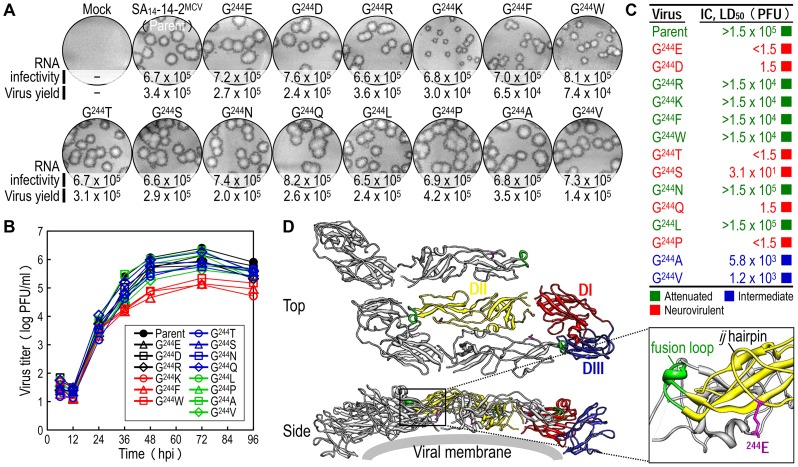
E-244: A key neurovirulence factor located in the *ij* hairpin of the viral E glycoprotein. (**A**) RNA infectivity and replicability. BHK-21 cells were mock-transfected or transfected with RNAs transcribed from the parent or one of the 14 E-244 mutant cDNAs. RNA infectivity (in PFU/µg) at 4 dpt was estimated by infectious center assay, coupled with staining of cell monolayers using an α-JEV antiserum, and virus yield (in PFU/ml) at 22 hpt was determined by plaque titration on BHK-21 cells. (**B**) Viral growth. BHK-21 cells were infected at an MOI of 0.1 with the parent or one of the 14 E-244 mutant viruses. At the indicated time points, culture supernatants were used for virus titration on BHK-21 cells. (**C**) Neurovirulence. Groups of 3-week-old ICR mice (*n* = 10 per group) were infected IC with serial 10-fold dilutions of each virus stock, and the LD_50_ values were determined. (**D**) Homology model. The predicted model of the E ectodomain of JEV SA_14_-14-2 was built based on the crystal structure of the E ectodomain of WNV NY99 [79], and the model was then fitted into the cryo-EM structure of WNV NY99 [18]. Illustrated is an icosahedral asymmetric unit of the three E monomers on the viral membrane. Highlighted in the inset is the critical residue Glu at E-244 in the *ij* hairpin adjacent to the fusion loop of the viral E DII.

To probe the functional importance of the amino acid side chain at position E-244 for the viral replication and neurovirulence of SA_14_-14-2^MCV^, we performed site-directed mutagenesis, replacing G^244^ with 14 other amino acids of six different classes: (*1*) aliphatic A, V, and L; (*2*) hydroxyl S and T; (*3*) cyclic P; (*4*) aromatic F and W; (*5*) basic R and K; and (*6*) acidic and their amides D, E, N, and Q. We first tested the viability of synthetic RNAs transcribed *in vitro* from the corresponding mutant cDNAs by measuring their infectivity after transfection of BHK-21 cells. In all cases, the mutant RNA was as viable as the parent RNA, with a specific infectivity of 6.5–8.2×10^5^ PFU/µg (Fig. 5A, RNA infectivity). However, three mutants (G^244^K, G^244^F, and G^244^W) were noticeably different from the parent and the other 11 mutants, as demonstrated by a ∼10-fold decrease in the yield of progeny virions released into culture medium during the first 22 hpt (Fig. 5A, virus yield) and a ∼2-2.5-fold reduction in the size of foci/plaques produced at 96 hpt (Fig. 5A, foci/plaques), although no significant difference was observed in the level of viral proteins (i.e., C, prM, E, and NS1) accumulated in RNA-transfected cells at 18 hpt (Fig. S5). As compared to G^244^K, the mutant G^244^R exhibited a barely marginal decrease in focus/plaque size and no detectable change in virus production (Fig. 5A). Overall, these findings were more evident when all mutant viruses were evaluated in multistep growth assays over the course of 96 h after infection at an MOI of 0.1, assessing their ability to grow and establish a productive infection (Fig. 5B). Our findings indicate that in BHK-21 cells, the amino acid side chain at position E-244 has no effect on the viability of the mutant RNAs, although it has a negative impact on the production and spread of infectious virions in the case of the three mutants G^244^K, G^244^F, and G^244^W.

In mice, we determined the neurovirulence of our 14 mutant viruses by IC inoculating groups of 3-week-old ICR mice (*n* = 10 per group) with various doses ranging from 1.5 to 1.5×10^4^ or 10^5^ PFU/mouse of the parent or each mutant virus. According to their IC LD_50_ values, the 14 mutant viruses are classified into three groups (Fig. 5C and Table S3): (*i*) group 1 (six mutants), neurovirulent, with an IC LD_50_ of ≤1.5 to 31 PFU, exemplified by replacing G^244^ with E, D, T, S, Q, and P; (*ii*) group 2 (six mutants), non-neurovirulent or neuroattenuated, with an IC LD_50_ of >1.5×10^4^ or 10^5^ PFU, behaving like the parent SA_14_-14-2^MCV^ and exemplified by exchanging G^244^ with R, K, F, W, N, and L; and (*iii*) group 3 (two mutants), with an intermediate phenotype and an IC LD_50_ of 1.2–5.8×10^3^ PFU, exemplified by substituting G^244^ with A and V. We confirmed the presence or absence of viral replication in the brain tissues of all dead or surviving mice, respectively; all mock-infected mice survived with no signs of disease (data not shown). Also, the mutation and phenotype relationship was corroborated by sequence analysis of recovered viruses from brain tissues of moribund or dead mice following IC inoculation. We analyzed all of the 14 mutants except for four group 2 mutants (G^244^R, G^244^F, G^244^W, and G^244^L), which failed to produce a lethal infection. In each case, the complete 2,001-nucleotide coding region of the prM and E genes was amplified from each of four randomly selected brain samples, followed by cloning and sequencing of at least seven independent clones per brain sample. In all six group 1 and two group 3 mutants, we found that the initial mutations introduced at the G^244^ codon were maintained with no second-site mutations, consistent with the high and intermediate levels of their neurovirulent phenotype (Table 1). In the remaining two group 2 mutants (G^244^K and G^244^N), however, a majority of the sequenced clones contained a point mutation in the same codon that led to an amino acid substitution (i.e., K→E/T and N→D, respectively), converting both mutants into neurovirulent viruses and highlighting the biological importance of the amino acid at position E-244 for neurovirulence (Table 1).

**Table 1:** Genetic stability of 14 E-244 mutants recovered from mouse brains after IC inoculation.

Input virus				Recovered virus	
	Virus	Initial codon	(amino acid)	Nucleotide change (amino acid change)	No. of independent clones
*A*	SA_14_-14-2^MCV^ (Parent)	^1707^GGG^1709^	(Gly)		
*N*	G^244^E	GAG	(Glu)	None	28/28
*N*	G^244^D	GAC	(Asp)	None	30/30
*A*	G^244^R	AGA	(Arg)	N.A.	
*A*	G^244^K	AAG	(Lys)	None	1/30
				AAG→GAG (Lys→Glu)	25/30
				AAG→ACG (Lys→Thr)	4/30
*A*	G^244^F	UUC	(Phe)	N.A.	
*A*	G^244^W	UGG	(Trp)	N.A.	
*N*	G^244^T	ACG	(Thr)	None	30/30
*N*	G^244^S	AGC	(Ser)	None	30/30
*A*	G^244^N	AAC	(Asn)	None	8/30
				AAC→GAC (Asn→Asp)	22/30
*N*	G^244^Q	CAG	(Gln)	None	30/30
*A*	G^244^L	CUG	(Leu)	N.A.	
*N*	G^244^P	CCG	(Pro)	None	28/28
*I*	G^244^A	GCC	(Ala)	None	30/30
*I*	G^244^V	GUC	(Val)	None	30/30

*A*, Attenuated; *I*, Intermediate; *N*, Neurovirulent.

N.A., Not available.

We next performed homology modeling to gain insight into the structural basis of E-244 function. The 3D model of the E monomer of JEV SA_14_-14-2 was constructed using the 3.0-Å crystal structure of the E monomer of WNV NY99 [79] as a template, with 75.5% sequence identity. The model was then fitted into the outer layer of the cryo-electron microscopy (EM) structure of WNV NY99 [18], thereby visualizing three monomers placed into an icosahedral asymmetric unit on the viral membrane. In each E monomer of SA_14_-14-2 containing three domains (DI, DII, and DIII), we noted that E-244 lies within the *ij* hairpin adjacent to the fusion loop at the tip of DII, with its amino acid side chain pointing toward the viral membrane (Fig. 5D). We also confirmed the location of E-244 in the crystal structure of the E ectodomain of JEV SA_14_-14-2 [80] that has been described recently (Fig. S6).

### Elucidation of the functional role of E-244 during JEV infection of neuronal cells *in vitro*

**Figure 6:**
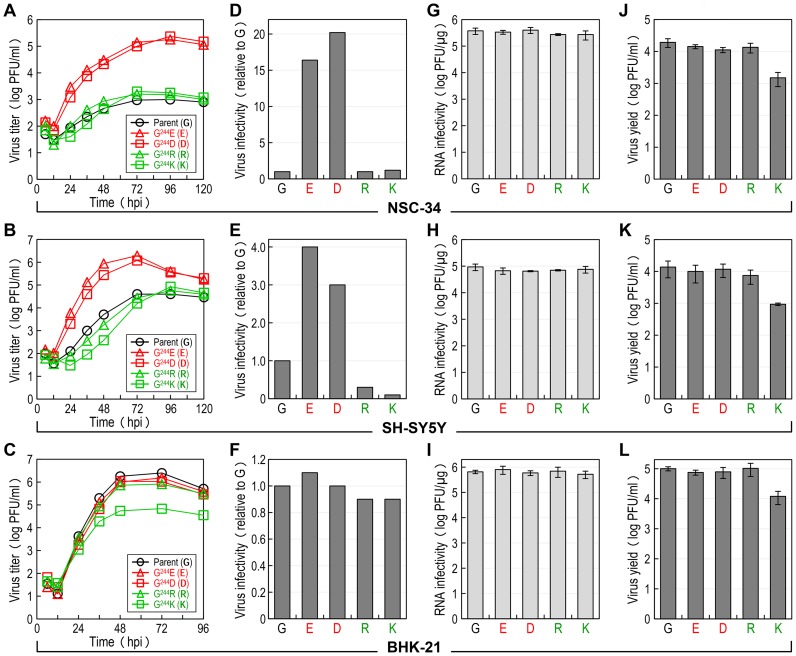
E-244: A major determinant of viral infectivity in mouse and human neuronal cells *in vitro*. (**A**-**C**) Viral growth. NSC-34 (**A**), SH-SY5Y (**B**), or BHK-21 (**C**) cells were infected at an MOI of 0.1 with the parent or one of the following four E-244 mutant viruses: G^244^E, G^244^D, G^244^R, and G^244^K. At the indicated time points, culture supernatants were harvested for virus titration on BHK-21 cells. (**D**-**F**) Viral particle infectivity. NSC-34 (**D**), SH-SY5Y (**E**), or BHK-21 (**F**) cells were infected at an MOI of 1 with each of the five viruses, as indicated. At 12–15 hpi, the number of infected cells was measured by flow cytometry using mouse α-JEV antiserum. The results are the average of two independent experiments, presented as -fold changes in infectivity relative to the parent (infectivity value of 1). (**G**-**I**) Viral RNA infectivity/replicability. NSC-34 (**G**), SH-SY5Y (**H**), or BHK-21 (**I**) cells were transfected with RNAs transcribed from the parent or each mutant cDNA, as indicated. At 4 dpt, RNA infectivity was quantified by infectious center assay, coupled with staining of cell monolayers using an α-JEV antiserum. (**J**-**L**) Virus yield. At 20 h after RNA transfection, culture supernatants from RNA-transfected NSC-34 (**J**), SH-SY5Y (**K**), or BHK-21 (**L**) cells were harvested for virus titration on BHK-21 cells.

We hypothesized that E-244, located at the *ij* hairpin of the viral E glycoprotein, plays an important role in JEV infection of neuronal cells. To test this hypothesis, we performed multistep growth assays in two neuronal cells, NSC-34 (mouse motor neuron) and SH-SY5Y (human neuroblastoma), by infecting at an MOI of 0.1 with the non-neurovirulent parent SA_14_-14-2^MCV^ and each of the four representative E-244 mutant viruses, i.e., two neurovirulent (G^244^E and G^244^D) and two non-neurovirulent (G^244^R and G^244^K). In parallel, the non-neuronal BHK-21 cells were also infected for comparison with the same set of five viruses. In NSC-34 cells, while the two neurovirulent viruses grew rapidly and reached their maximum titers of 1.8–2.4×10^5^ PFU/ml at 72–96 hours post-infection (hpi), the three non-neurovirulent viruses, including the parent, all replicated poorly, with peak titers only approaching 1.0–2.0×10^3^ PFU/ml, ∼100-fold lower than those of the two neurovirulent viruses (Fig. 6A). In SH-SY5Y cells, a similar defect in viral growth was also observed, with a ∼50- to 100-fold difference in maximum virus titers between the neurovirulent and the non-neurovirulent viruses. In addition, we noted a differential growth defect in the three non-neurovirulent viruses, with G^244^R replicating more poorly than the parent but better than G^244^K (Fig. 6B). In contrast to the pattern of viral growth observed in NSC-34 and SH-SY5Y cells, we found that in BHK-21 cells, only G^244^K had a noticeable defect in viral growth, with ∼20-fold lower peak titers than those of the other four viruses that grew well to maximum titers of 0.8–2.5×10^6^ PFU/ml at 48–72 hpi (Fig. 6C). These data indicate that E-244 plays a crucial role in the productive infection of JEV in neuronal cells.

Subsequently, we examined the infectivity/replicability of the parent and its four E-244 mutant viruses/RNAs in NSC-34 and SH-SY5Y cells, in parallel with BHK-21 cells for comparison. First, virus infectivity was quantified using flow cytometry by infecting the three cell types at an MOI of 1 with each of the five viruses and counting the number of cells stained with a mouse α-JEV antiserum at 12–15 hpi. In NSC-34 cells, the two non-neurovirulent mutants (G^244^R and G^244^K) exhibited infectivities nearly identical to that of the non-neurovirulent parent (Fig. 6D), whereas the two neurovirulent mutants (G^244^E and G^244^D) showed infectivities ∼16- to 20-fold higher than that of the non-neurovirulent parent. Similarly, the E-244 mutation also altered virus infectivity in SH-SY5Y cells. Specifically, the two neurovirulent mutants had ∼3- to 4-fold higher infectivities than the non-neurovirulent parent; on the other hand, the two non-neurovirulent mutants displayed even lower infectivities than the parent (∼3-fold for G^244^R and ∼10-fold for G^244^K) (Fig. 6E). In contrast, no significant difference in virus infectivity was observed among all five viruses in BHK-21 cells (Fig. 6F).

Next, the replication efficiency of the viral genomic RNA was quantified by directly transfecting the three cell types with each of the five synthetic RNAs transcribed *in vitro* from the respective JEV cDNAs and estimating the number of infectious foci stained with the mouse α-JEV antiserum at 4 days post-transfection (dpt). In each of the three cell types, there was no detectable difference in the specific infectivity of the five RNAs (NSC-34, Fig. 6G; SH-SY5Y, Fig. 6H; and BHK-21, Fig. 6I). Also, quantitative real-time RT-PCRs indicated that the level of the viral genomic RNAs accumulated in the RNA-transfected cells over the first 15 h of transfection was indistinguishable between the parent and the four different E-244 mutants (data not shown). Regardless of cell type, however, the G^244^K mutant was different from the parent and the other three E-244 mutants, as demonstrated by a ∼1-log decrease in the yield of infectious virions released into culture medium during the first 20 hpt (NSC-34, Fig. 6J; SH-SY5Y, Fig. 6K; and BHK-21, Fig. 6L). Overall, these results show that the E-244 mutation alters JEV infectivity in a neuronal cell-specific manner, in agreement with the neurovirulence phenotype observed in mice, and it also affects infectious particle production in a cell type-nonspecific manner.

### Comparison of the amino acid sequences of the *ij* hairpin in encephalitic and non-encephalitic flaviviruses

**Figure 7:**
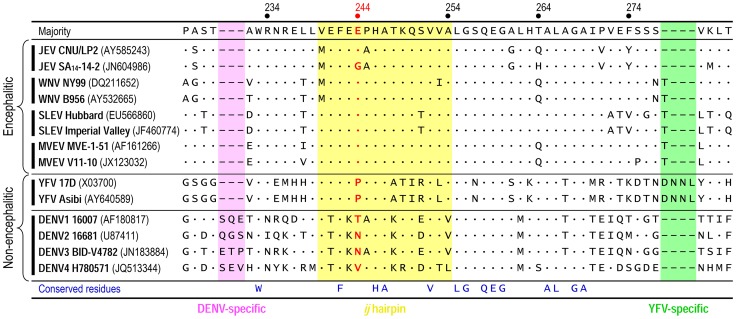
Structure-based, *ij*-hairpin amino acid sequence alignment for six representative flaviviruses (14 strains total): JEV, WNV, SLEV, MVEV, YFV, and DENV. All sequence information was retrieved from the GenBank database with accession numbers indicated. Multiple sequence alignments were performed using ClustalX [116]. Highlighted are the ∼15-aa *ij*-hairpin (yellow), the 3-aa DENV-specific motif (pink), and the 4-aa YFV-specific motif (green). The consensus sequence of the *ij* hairpin and its flanking region is presented on top, and only differences from that sequence are shown. Deletions are indicated by hyphens. The amino acid residue is numbered based on the JEV SA_14_-14-2 (GenBank accession no. JN604986).

We initially generated a multiple sequence alignment using all 154 full-length JEV genomes available from the GenBank sequence database. Of note is the fact that SA_14_ and SA_14_-14-2 have been fully sequenced by three and four independent groups, respectively; their nucleotide and deduced amino acid sequences are not identical [63]–[65], [77], [81]. The sequence alignment showed a Glu residue at position E-244 in the *ij* hairpin of all JEV strains isolated from infected mosquitoes, pigs, or humans, except for the Gln-encoding mosquito-derived K94P05 and three Gly-encoding SA_14_-derived attenuated strains (i.e., SA_14_-2-8, SA_14_-12-1-7, and all four different versions of SA_14_-14-2) (Fig. S7). In case of SA_14_, it is intriguing to note that one version has a Glu residue at position E-244, but the other two versions have a Gly residue at that position (Fig. S7); this discrepancy is likely due to variations in the cultivation history of the virus [77]. We next performed the structure-based, *ij*-hairpin amino acid sequence alignment with six representative flaviviruses (14 strains total), including four encephalitic (JEV, WNV, SLEV, and MVEV) and two non-encephalitic (YFV and DENV) flaviviruses. In addition to the importance of the E-244 amino acid, we noted (*i*) the evolutionally conserved residues in the *ij* hairpin and its flanking region in all six flaviviruses, i.e., W^233^, F^242^, H^246^, A^247^, V^252^, L^255^, G^256^, Q^258^, E^259^, and G^260^; (*ii*) the sequence similarities in the four encephalitic flaviviruses, particularly in a ∼15-aa *ij*-hairpin-containing region; and (*iii*) the sequence differences between the four encephalitic and two non-encephalitic flaviviruses, e.g., the 4-aa YFV-specific motif and the 3-aa DENV-specific motif (Fig. 7). Overall, our findings suggest that the *ij* hairpin of the E DII plays a key role in determining encephalitic flavivirus neurovirulence, and its function is regulated by the chemical properties of the amino acid at position E-244 in that hairpin.

## Discussion

In this work, we have developed a reverse genetics system for SA_14_-14-2, a live-attenuated JE vaccine [53], [82], by constructing an infectious cDNA and rescuing molecularly cloned virus from the cDNA. This reverse genetics system offers us a unique opportunity to elucidate the genetic and molecular basis of JEV neurovirulence. Using our infectious SA_14_-14-2 cDNA technology, we (*i*) generated three isogenic SA_14_-14-2 variants that unlike its parent, displayed lethal neurovirulence in mice; (*ii*) identified a single point mutation, G^1708^→A, causing a Gly→Glu change at position 244 of the viral E glycoprotein that is sufficient to confer a full neurovirulence by promoting viral infection into neurons in the mouse CNS *in vivo* and mouse/human neuronal cells *in vitro*; and (*iii*) demonstrated the structure-function relationship for neurovirulence of E-244 in the *ij* hairpin adjacent to the fusion loop at the tip of the viral E DII. Thus, our findings reveal fundamental insights into the neurotropism and neurovirulence of JEV and other taxonomically related encephalitic flaviviruses, including WNV, SLEV, and MVEV. Intriguingly, our results also provide a new target, the *ij* hairpin, for the development of novel antivirals for the prevention and treatment of infection with the encephalitic flaviviruses.

The flavivirus glycoprotein E mediates receptor-mediated endocytosis and low pH-triggered membrane fusion [33], [83], [84]. On the viral membrane, 180 E monomers are packed into 30 protein “rafts”, each composed of three E head-to-tail homodimers [17]–[19]. Each E monomer is composed of three parts: (*i*) an elongated ectodomain that directs receptor binding and membrane fusion; (*ii*) a “stem” region containing two amphipathic α-helices that lies flat on the viral membrane underneath the ectodomain; and (*iii*) a membrane “anchor” region containing two transmembrane antiparallel coiled-coils. The E ectodomain folds into three β-barrel domains [85]: (*i*) DI, a structural domain centrally located in the molecule; (*ii*) DII, an elongated dimerization domain containing the highly conserved fusion loop at its tip [86]; and (*iii*) DIII, an Ig-like domain implicated in receptor binding [20], [87], [88] and antibody neutralization [89]–[92]. Based on pre- and post-fusion crystal structures of the ectodomain [28], [30], [31], [47], [79], [80], [85] and biochemical analyses [29], [32], [93], a current, detailed model for flavivirus membrane fusion has been developed. In this model, the fusion is initiated by a low pH-induced dissociation of the antiparallel E homodimers that leads to the exposure of the fusion loops and their insertion into the host membrane, followed by a large-scale structural rearrangement into a parallel E homotrimer [33], [83], [84], [94]. In the parallel conformation, DIII folds back toward DII, presumably with the stem extended from the C-terminus of DIII along DII and toward the fusion loop (“zipping”), driving the fusion of the viral and host membranes [95]–[98]. Despite our detailed knowledge about the fusion process, there is little available structural information about how flaviviruses bind to their cellular receptors. In encephalitic flaviviruses, the presence of an RGD motif in DIII and carbohydrate moieties on the viral surface suggests a mechanism involving interaction with the RGD motif-recognizing integrins and sugar-binding lectins on the cell surface, respectively. However, blocking/alteration of either the RGD motif or glycan does not abolish viral entry [22], [99]–[101]. Thus, the viral factors and the interacting cellular counterparts required for viral entry are still elusive.

In flaviviruses, the *ij* hairpin is a structural motif that is closely associated with the fusion loop at the tip of the viral E DII, but its role is thus far unknown. In JEV, we now report that a single amino acid in the *ij* hairpin, E-244, serves as a key regulator to control the level of neurovirulence of SA_14_-14-2 in mice. This amino acid was also correlated with a differential ability to infect neurons, the primary target cells in the CNS. Consistent with this finding, we found that site-directed mutagenesis of the codon for E-244 in SA_14_-14-2 created a panel of 14 recombinant viruses of varying neurovirulence: (*i*) non-neurovirulent viruses, produced by substitutions of positively-charged (R, K), aromatic (F, W), polar (N), or aliphatic (L) residues; (*ii*) neurovirulent viruses, produced by substitutions of negatively-charged (E, D), hydroxyl (T, S), polar (Q), or cyclic (P) residues; and (*iii*) viruses intermediate in neurovirulence, produced by substitutions of aliphatic (A, V) residues. These results highlight the role of E-244 in neurovirulence, which was directed by a combination of three major properties of its amino acid side chain: (*i*) charge (R/K vs. E/D); (*ii*) size (N vs. Q and L vs. A/V); and (*iii*) functional group (N vs. D). Our data suggest that the *ij* hairpin acts as a viral factor that promotes JEV infection of neurons within the CNS, likely through its role in one of three major steps involved in viral entry: binding, endocytosis, or membrane fusion [33], [83], [84]. Alternatively, it is possible that the late steps in the virus life cycle in neurons, such as assembly, maturation, and release, could be affected. For JEV, WNV, and tick-borne encephalitis virus, the assembly/release of infectious virions or virus-like particles has been shown to be affected by the N-glycosylation of the viral prM and/or E protein in non-neuronal cells [44], [75], [102]–[104]. Moreover, a conserved single N-glycosylation site in the JEV prM protein has been shown to be important for viral pathogenicity in mice [75].

Over the years, the virulence of JEV has been an active area of research. Initially, comparison of the genomic sequences of several JEV strains with a different degree of pathogenicity had predicted a number of potential loci in the viral genome that are involved in virulence [63]–[74]. Due to the complexity and variation of the mutations, however, the identity of the major viral factor that is critical for JEV virulence remains unclear. In particular, SA_14_-14-2 has been reported to have a total of 47–64 nucleotide changes (17–27 amino acid substitutions) when compared to its virulent parental strain SA_14_; the number of mutations varies and depends on the cultivation history of the viruses [63]–[65]. Of the ten viral proteins, the E protein has been the primary target of genetic studies in virulence, mainly because it is involved in cell/tissue tropism and pathogenesis. Several amino acid residues in the E protein have been suggested to contribute to the neurovirulence and/or neuroinvasiveness of JEV *in vivo*: (*i*) E-123, illustrated by an S^123^R substitution that is capable of enhancing the neuroinvasiveness of the Mie/41/2002 strain in 3-week-old ddY mice [105]; (*ii*) E-279, exemplified by an M^279^K mutation that is able to increase the neurovirulence of ChimeriVax-JE (a chimeric virus that carries the prM and E genes of JEV SA_14_-14-2 on a YFV 17D genetic background) in suckling mice and rhesus monkeys [106]; and (*iii*) E-138, indicated by two reciprocal mutations: (1) a K^138^E substitution, when combined with at least two other mutations, which elevates the neurovirulence of the ChimeriVax-JE virus in 4-week-old ICR mice [107], and (2) an E^138^K substitution, which lowers the neurovirulence and neuroinvasiveness of three different JEVs (the JaOArS982 strain in 2- to 5-week-old Swiss ICR mice [73] and the AT31 and NT109 strains in 3-week-old BALB/c mice [108], [109]). These data indicate that multiple amino acid residues in the E protein of JEV function in a more coordinated way to achieve the maximal level of neurovirulence and/or neuroinvasiveness [53], [107]. This notion is consistent with our finding that although a single G^244^E mutation in the E protein of SA_14_-14-2 is sufficient to confer lethal neurovirulence in 3-week-old ICR mice, the spread of the virus in the brains is still slow and limited, as compared to the highly virulent CNU/LP2 strain. These and previous findings suggest that in addition to E-244, other amino acid residues in the E protein play a role in determining the neurovirulence of SA_14_-14-2. In addition, JEV NS1' (a product of ribosomal frameshifting [110], [111]) is reported to be produced in cells infected with SA_14_ but not with SA_14_-14-2, and its lack of expression is shown to contribute to the attenuation phenotype of SA_14_-14-2 in mice [112]. Similarly, the expression of NS1' is also suggested to be associated with the neuroinvasiveness of WNV [110].

The neuroattenuation phenotype of SA_14_-14-2 has been tested in several laboratory animals, including mice and monkeys [53]. In 2- to 4-week-old ICR and ddY mice, no morbidity or mortality has been observed after subcutaneous and intracerebral inoculations with 10^4^-10^6^ PFU of SA_14_-14-2 [53], [63], [113]; in a rare case, however, the virus was found to be able to cause the death of the mice following IC inoculation [113]. In line with these previous results, we also found that none of the 3-week-old ICR mice inoculated IM or IP with up to ∼10^5^ PFU of SA_14_-14-2 showed clinical signs or death; on the other hand, although there was some variability among the groups of mice and the doses of virus inoculum, ∼5–30% of the mice inoculated IC with a dose of 10^3^–10^5^ PFU developed JEV-specific symptoms and death. This low but unexpected morbidity and mortality after the IC inoculation of SA_14_-14-2 is likely caused by a combination of factors and conditions imposed on our infection experiments, particularly the age and strain of mice: (*i*) Age-dependent susceptibility of flaviviruses, including JEV, in the murine model has been documented previously, although its molecular mechanisms remain unclear [106], [114], [115]. (*ii*) A noticeable variability in mortality has also been reported when two different lineages of the age-matched outbred ICR mice are inoculated IC with a mutant of ChimeriVax-JE that contains two amino acid substitutions (F^107^L and K^138^E) in the SA_14_-14-2 E protein-coding region, suggesting that differences in the genetic background of mice may account for the variable neurovirulence [107]. More importantly, it should be pointed out that in our study, all the revertants recovered from the mice inoculated IC with SA_14_-14-2 appeared to have the G^244^E mutation, which is sufficient to confer lethal neurovirulence to the virus, corroborating that the parental SA_14_-14-2 virus is highly attenuated in neurovirulence. Furthermore, it is intriguing to note that the G^244^E substitution has been introduced into ChimeriVax-JE, in which no mortality occurs when eight 4-week-old ICR mice are injected IC with 10^4^ PFU of the mutant virus [107]; therefore, it appears that neurovirulence may depend on the genetic background of the pathogen. Further investigation is needed to fully elucidate the neurovirulence and neuroinvasiveness of JEV.

In summary, we show for the first time that E-244 in the *ij* hairpin of the viral E DII is a key regulator determining the neurovirulence of SA_14_-14-2, and we also provide direct evidence that viral E can contribute to the neurovirulence of JEV and possibly other closely related encephalitic flaviviruses via its role in the early or late stage of viral replication in neurons. A detailed, complete understanding of the evolutionally conserved viral *ij* hairpin and its function in the virus life cycle will have direct application to the design of a novel and promising class of broad-spectrum antivirals (e.g., ligands and small molecules) to expand the currently available preventive and therapeutic arsenal against infection with encephalitic flaviviruses.

## Materials and Methods

### Viruses and cells

An original stock of JEV SA_14_-14-2 was retrieved directly from a batch of commercial vaccine vials (Chengdu Institute of Biological Products, China) for viral genome sequencing and cDNA construction, to avoid any potential concern that its adaptation could occur during propagation in cell culture. This virus stock was propagated twice in BHK-21 cells to generate high-titer viral preparations for cell and mouse infection experiments. Stocks of JEV CNU/LP2 were derived from the infectious cDNA pBAC^SP6^/JVFLx/XbaI [78]. BHK-21 cells were grown in alpha minimal essential medium (α-MEM) containing 10% fetal bovine serum (FBS), 2 mM L-glutamine, vitamins, and penicillin-streptomycin at 37°C in 5% CO_2_ [78]. SH-SY5Y cells were cultivated in a 1∶1 mixture of MEM and Ham's F-12 nutrient mix supplemented with 10% FBS, 0.1 mM nonessential amino acids, and penicillin-streptomycin at 37°C in 5% CO_2_ [75]. NSC-34 cells were maintained in Dulbecco's modified Eagle's medium containing 10% FBS and penicillin-streptomycin at 37°C in 5% CO_2_.

### JEV reverse genetics

As a vector, we used the BAC plasmid pBeloBAC11 [78]. First, four cDNA fragments covering the entire viral genome were cloned into the vector individually, then joined sequentially at three natural restriction sites (*Bsr*GI, *Bam*HI, and *Ava*I) to generate a single BAC clone that contained the full-length SA_14_-14-2 cDNA, named pBAC/SA_14_-14-2 (Fig. 1). The SP6 promoter sequence was positioned just upstream of the viral 5′-end, and an artificial *Xba*I run-off site was engineered just downstream of the viral 3′-end. A pre-existing *Xba*I site at nucleotide 9131 was removed by introducing a silent point mutation (A^9134^→T); this mutation also served as a rescue marker to identify the cDNA-derived SA_14_-14-2. All mutations were created by overlap extension PCR. All PCR-generated fragments were sequenced. Detailed cloning procedures are described in Supporting Information.

### RNA transcription and transfection

All BAC plasmids were purified by centrifugation using CsCl-ethidium bromide equilibrium density gradients. The closed circular plasmids were linearized by *Xba*I and mung bean nuclease digestion to produce DNA templates for *in vitro* run-off transcription. RNA was transcribed from a linearized plasmid with SP6 RNA polymerase as described [78]. The resulting RNA was stored at −80°C until needed. RNA yield was measured on the basis of the incorporation rate of [^3^H]UTP, and RNA integrity was evaluated by agarose gel electrophoresis. RNA was transfected by electroporation into cells under our optimized conditions (980 V, a 99-µs pulse length, and five pulses for BHK-21 cells; and 760 V, a 99-µs pulse length, and five pulses for NSC-34 and SH-SY5Y cells) [78]. RNA infectivity was determined by infectious center assay as reported [78]. The infectious centers of foci were detected by decorating of cells with a mouse α-JEV antibody (American Type Culture Collection [ATCC], 1∶500) and a horseradish peroxidase-conjugated goat α-mouse IgG (Jackson ImmunoResearch, 1∶1,000), followed by staining with 3,3′-diaminobenzidine (Vector).

### Northern blots

Total RNA was extracted with TRIzol reagent (Invitrogen). Northern blot analysis was performed as described [78]. JEV genomic RNA was detected with an antisense riboprobe that binds to a 209-bp region (nt 9143–9351) in the NS5 protein-coding region. The probe was synthesized with [α-^32^P]CTP by using the T7-MEGAscript kit (Ambion). The blots were prehybridized, hybridized, and washed at 55°C. Autoradiographs were obtained by exposure to film for 24–48 h.

### Immunoblots

Cells were lysed in sample buffer (80 mM Tri-HCl [pH 6.8], 2.0% SDS, 10% glycerol, 0.1 M dithiothreitol, 0.2% bromophenol blue). Equal amounts of the lysates were run on SDS-polyacrylamide gels, transferred to polyvinylidene difluoride membranes, and subjected to immunoblotting as described [78]. The following polyclonal antisera were used as primary antibodies [75], [76]: α-JEV (mouse, 1∶1,000), α-C (rabbit, 1∶1,000), α-pr (rabbit, 1∶4,000), α-E (rabbit, 1∶500), α-NS1 (rabbit, 1∶1,000), and α-GAPDH (rabbit, 1∶10,000). An alkaline phosphatase-conjugated goat α-mouse or α-rabbit IgG (Jackson ImmunoResearch, 1∶5,000) was used for the secondary antibody, as appropriate. The specific signals were visualized by chromogenic membrane staining with a mixture of 5-bromo-4-chloro-3-indolyl-phosphate and nitroblue tetrazolium (Sigma-Aldrich).

### Flow cytometry

Cells (5×10^5^) were harvested by trypsinization, washed with phosphate-buffered saline (PBS), and collected by centrifugation at 1,000×*g* for 5 min. The cells were resuspended in 250 µl of Cytofix/Cytoperm solution (BD Biosciences) and incubated at 4°C for 20 min in the dark. All subsequent wash and staining steps were performed in Perm/Wash buffer (BD Biosciences). The cells were washed twice and incubated in 200 µl of mouse α-JEV antiserum (ATCC, 1∶500) for 1 h at 4°C. Subsequently, the cells were washed twice and incubated in 200 µl of Alexa Fluor 488 goat α-mouse IgG (Molecular Probes, 1∶1,000) for 1 h at 4°C. The cells were then washed twice and resuspended in 200 µl of Perm/Wash buffer. The samples were analyzed on a FACSAriaIII cell sorter with Diva 6.1.3 software (BD Biosciences). For each sample, 50,000 events were collected within the linear range of detection.

### Sequence analysis

The full genome sequences of SA_14_-14-2 and its neurovirulent variants were determined as described [77]. Sequencing of the prM-E coding region of the E-244 mutants was done as follows: (*i*) amplification of a 2,069-bp cDNA by RT-PCR using a set of three primers (prMErt, prMEfw, and prMErv; see Table S4); (*ii*) cloning of a 2,057-bp *Xho*I-*Sac*II fragment into the pRS2 vector; and (*iii*) sequencing of ∼30 randomly picked independent clones containing the insert. Multiple sequence alignments were performed using ClustalX [116].

### Mouse infection

Female 3-week-old ICR mice (Charles River) were used. Groups of 10 or 20 mice were inoculated IC (20 µl), IM (50 µl), or IP (50 µl) with 10-fold serial dilutions of virus stock in α-MEM. Mice were monitored for any JEV-induced clinical signs or death every 12 h for 24 days. The LD_50_ values were determined as described [75], [77]. In all mice, viral replication in brain tissue was confirmed by plaque titration and/or RT-PCR [75].

### Ethics statement

All animal studies were conducted in strict accordance with the regulations in the Guide for the Care and Use of Laboratory Animals issued by the Ministry of Health and Welfare of the Republic of Korea. The protocol was approved by the Institutional Animal Care and Use Committee of the Chungbuk National University Medical School (Permit Number: LML08-73). All mice were housed in our animal facility located at the Chungbuk National University Medical School, and every effort was made to minimize suffering.

### Immunohistochemistry

Groups of 3-week-old female ICR mice (*n* = 15 per group) were infected IC with 10^3^ PFU of virus in 20 µl of α-MEM; 10 control mice were inoculated IC with an equivalent volume of supernatant from uninfected control BHK-21 cell cultures at comparable dilution. At 3, 5, and 7 dpi, five randomly selected mice were transcardially perfused with ice-cold PBS, followed by 4% paraformaldehyde (PFA). Brains were fixed in 4% PFA, embedded in paraffin, and cut into 6-µm sections. Brain sections were treated in microwave for antigen retrieval and incubated with 1% H_2_O_2_ in ice-cold methanol for 30 min to block endogenous peroxidase. They were then blocked with 1% normal goat serum and incubated with rabbit α-NS1 antiserum (1∶200) for 12 h at 4°C, followed by incubation with biotinylated α-rabbit IgG plus the avidin-biotin-peroxidase complex (Vector). Signals were visualized by staining with 3,3′-diaminobenzidine solution containing 0.003% H_2_O_2_ and counterstaining with hematoxylin.

### Homology modeling

The sequence and structure of the E ectodomain of WNV NY99 (PDB accession code 2HG0) was used as template for the homology modeling. The sequence alignment was done using the online version of ClustalW2 [117]. Protein structure homology modeling was performed using the SWISS-MODEL Workspace, accessible via the ExPASy web server [118]. The generated model was visualized using UCSF Chimera 1.5.3. The model is in agreement with a recent crystal structure of the E ectodomain of JEV SA_14_-14-2 (PDB accession code 3P54) [80].

## Supporting Information

Figure S1 **Representative focus/plaque morphologies of SA_14_-14-2^MCV^.** BHK-21 cells were mock-infected or infected with one of the following three JEVs: SA_14_-14-2^MCV^, SA_14_-14-2, or CNU/LP2 (a virulent strain used as a reference). After infection, cells were overlaid with agarose to examine focus/plaque morphologies. At 4 dpi, cell monolayers were first immunostained with a mouse α-JEV antiserum to visualize the infectious foci, and the same monolayers were then restained with crystal violet to observe the infectious plaques. The average plaque sizes (mean ± SD) were determined by counting 10 representative plaques.
(PPT)

Figure S2 **Viral growth properties of SA_14_-14-2^MCV^ in SH-SY5Y and C6/36 cells.** Cells were infected at an MOI of 1 with the molecularly cloned virus (SA_14_-14-2^MCV^) rescued from the full-length infectious cDNA or the original parental virus (SA_14_-14-2) used for cDNA construction. Culture supernatants were collected at the hour postinfection (hpi) indicated, and virus titers were determined by plaque assays on BHK-21 cells.
(PPT)

Figure S3 **Virological properties of SA_14_-14-2^MCV^ in mice.** Groups of 3-week-old female ICR mice (*n* = 20 per group) were mock-inoculated or inoculated intracerebrally (IC), intramuscularly (IM), or intraperitoneally (IP) with serial 10-fold dilutions of SA_14_-14-2^MCV^, SA_14_-14-2, or CNU/LP2 (a virulent JEV strain used as a reference). Mice were observed for any JEV-induced clinical signs and death every 12 h for 24 days. Survival curves were plotted by the Kaplan-Meier method.
(PPT)

Figure S4 **A single point mutation promotes susceptibility to SA_14_-14-2^MCV^ infection of neurons in the CNS.** Groups of 3-week-old female ICR mice (*n* = 15 per group) were mock-infected or infected IC with 10^3^ PFU of SA_14_-14-2^MCV^ (Parent), G^1708^A, or CNU/LP2 (a virulent JEV strain used as a reference). On the indicated days after infection, five mice were subjected for immunostaining of JEV NS1 antigen in fixed brain slices with an α-NS1 antiserum. Presented are representative slides of amygdala, cerebral cortex, thalamus, hypothalamus, and brainstem (note that hippocampal slides are shown in Fig. 4E). Arrowheads indicate the NS1-positive cells.
(PPT)

Figure S5 **Levels of JEV protein accumulation in BHK-21 cells transfected with 14 E-244 mutant RNAs.** BHK-21 cells were mock-transfected or transfected with RNAs transcribed from SA_14_-14-2^MCV^ (Parent) or each of the 14 E-244 mutant cDNAs as indicated. At 18 hpt, viral protein accumulation was analyzed by immunoblotting of cell lysates with a panel of JEV-specific antisera. In parallel, GAPDH protein was used as a loading and transfer control.
(PPT)

Figure S6 **The location of E-244 on the crystal structure of the E ectodomain of JEV SA_14_-14-2.** The E ectodomain of JEV SA_14_-14-2: DI (colored red), DII (yellow), DIII (blue), and the fusion loop (green). The critical residue Gly at E-244 in the *ij* hairpin adjacent to the fusion loop of the viral E DII is shown. The crystal structure of the E ectodomain of JEV SA_14_-14-2 was retrieved from the RCSB Protein Data Bank (PDB accession code 3P54).
(PPT)

Figure S7 **Amino acid sequence alignment of 154 fully sequenced JEV strains at the conserved** ***ij*** **hairpin of viral E glycoprotein.** Multiple sequence alignments were performed using the amino acid sequence of 154 fully sequenced JEV genomes, including SA_14_ (red), SA_14_-14-2 (green), and two other SA_14_-derived attenuated strains, SA_14_-2-8 (orange) and SA_14_-12-1-7 (blue). Note that SA_14_ and SA_14_-14-2 have been sequenced by three and four independent research groups, respectively. The consensus sequence of the *ij* hairpin and its flanking region is presented on top, and only differences from that sequence are shown. Highlighted are the ∼15-aa *ij*-hairpin and the position E-244 in that hairpin.
(PPT)

Table S1 **Neurovirulence and neuroinvasiveness of SA_14_-14-2^MCV^ and its three variants in 3-week-old ICR mice.**
(PPT)

Table S2 **Neurovirulence of SA_14_-14-2^MCV^ and its eight mutants in 3-week-old ICR mice.**
(PPT)

Table S3 **Neurovirulence of SA_14_-14-2^MCV^ and its 14 E-244 mutants in 3-week-old ICR mice.**
(PPT)

Table S4 **Oligonucleotides used for ligation, cDNA synthesis, and PCR amplification.**
(PPT)
