# Correction: A Molecularly Cloned, Live-Attenuated Japanese Encephalitis Vaccine SA_14_-14-2 Virus: A Conserved Single Amino Acid in the *ij* Hairpin of the Viral E Glycoprotein Determines Neurovirulence in Mice

**DOI:** 10.1371/journal.ppat.1004465

**Published:** 2014-09-22

**Authors:** The PLOS Pathogens Staff

The file “Text S1” is omitted from the list of Supporting Information in the original article. It can be downloaded below. The reference to “Supporting Information” in the section entitled, “JEV reverse genetics”, refers to the supplementary materials and methods file, “Text S1”.

## Supporting Information

Text S1 Supplementary Materials and Methods.
(PDF)
