# Supplementary figures and images for: A Molecularly Cloned, Live-Attenuated Japanese Encephalitis Vaccine SA14-14-2 Virus: A Conserved Single Amino Acid in the ij Hairpin of the Viral E Glycoprotein Determines Neurovirulence in Mice
Source: PLoS Pathog. 2014 Jul 31;10(7):e1004290. doi: 10.1371/journal.ppat.1004290 (PMC4117607; doi:10.1371/journal.ppat.1004290)

## Slide 1
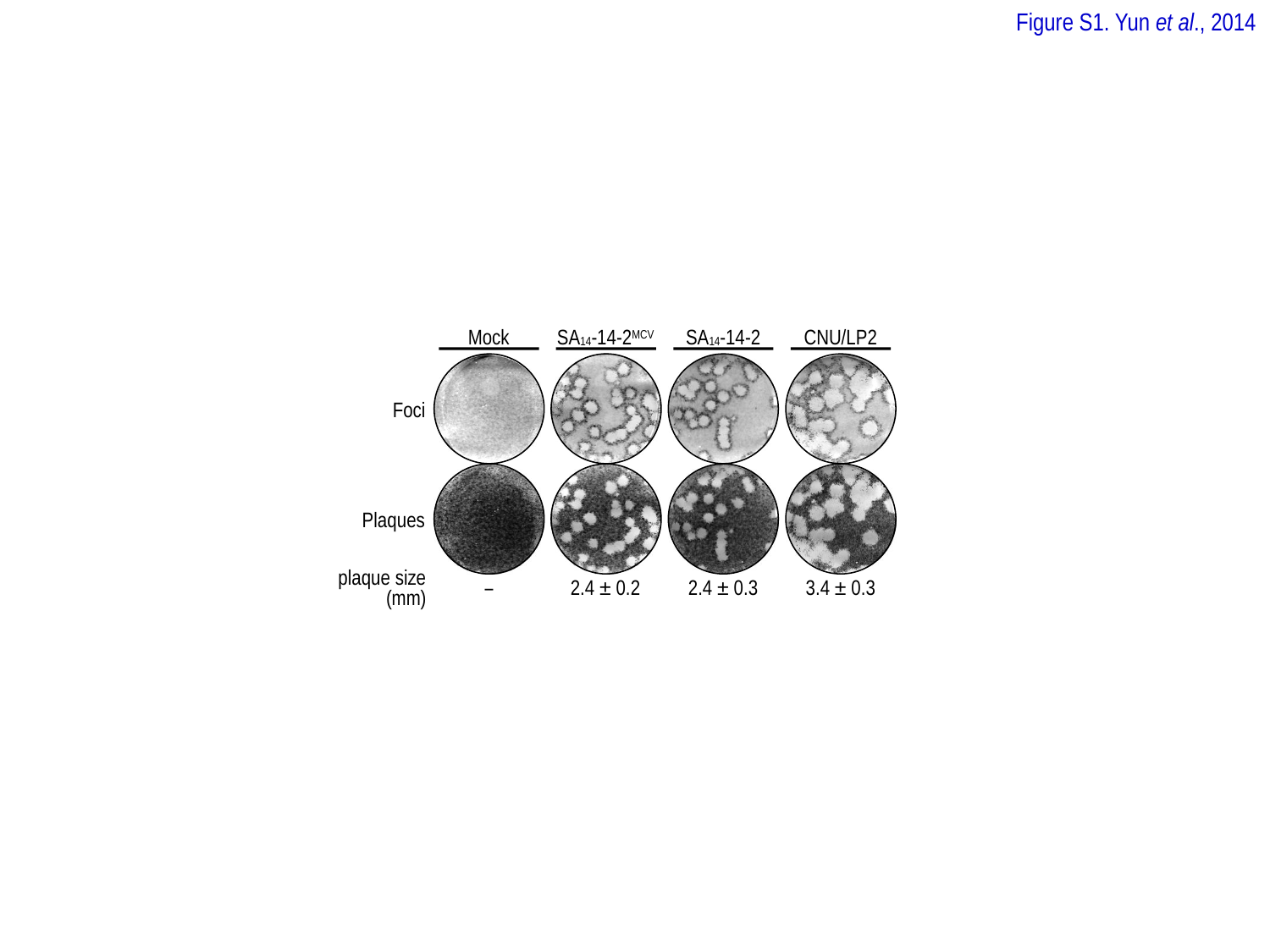

Figure S1. Yun et al., 2014
Mock
−
SA14-14-2MCV
2.4 ± 0.2
SA14-14-2
2.4 ± 0.3
CNU/LP2
3.4 ± 0.3
Foci
Plaques
plaque size
(mm)

Supplement: Figure S1 — Representative focus/plaque morphologies of SA14-14-2MCV. BHK-21 cells were mock-infected or infected with one of the following three JEVs: SA14-14-2MCV, SA14-14-2, or CNU/LP2 (a virulent strain used as a reference). After infection, cells were overlaid with agarose to examine focus/plaque morphologies. At 4 dpi, cell monolayers were first immunostained with a mouse α-JEV antiserum to visualize the infectious foci, and the same monolayers were then restained with crystal violet to observe the infectious plaques. The average plaque sizes (mean ± SD) were determined by counting 10 representative plaques. (PPT) [file ppat.1004290.s001.ppt]

## Slide 1
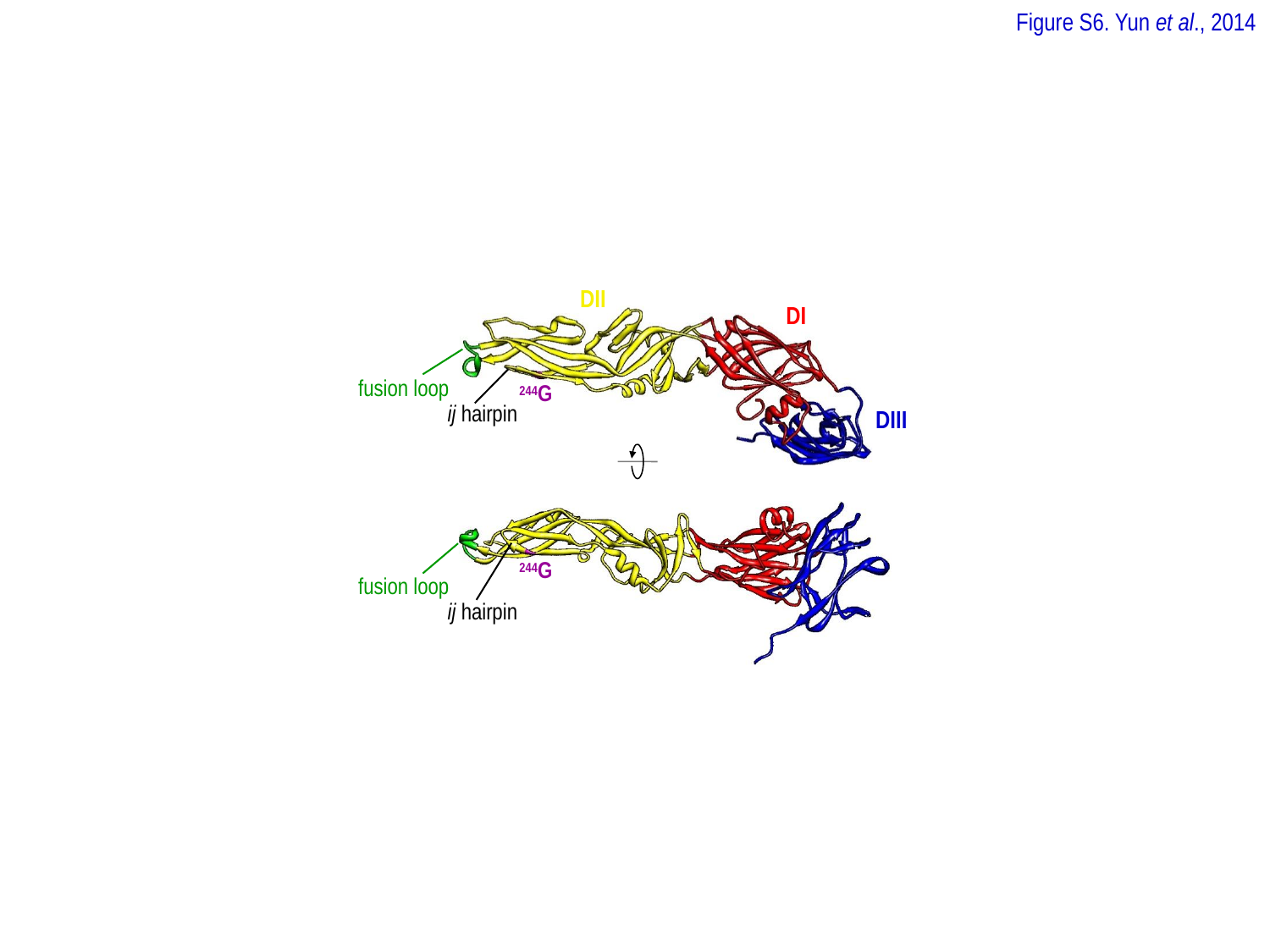

Figure S6. Yun et al., 2014
DII
DI
244G
DIII
244G
fusion loop
ij hairpin
fusion loop
ij hairpin

Supplement: Figure S6 — The location of E-244 on the crystal structure of the E ectodomain of JEV SA14-14-2. The E ectodomain of JEV SA14-14-2: DI (colored red), DII (yellow), DIII (blue), and the fusion loop (green). The critical residue Gly at E-244 in the ij hairpin adjacent to the fusion loop of the viral E DII is shown. The crystal structure of the E ectodomain of JEV SA14-14-2 was retrieved from the RCSB Protein Data Bank (PDB accession code 3P54). (PPT) [file ppat.1004290.s006.ppt]
