# Supplementary material for: A Molecularly Cloned, Live-Attenuated Japanese Encephalitis Vaccine SA14-14-2 Virus: A Conserved Single Amino Acid in the ij Hairpin of the Viral E Glycoprotein Determines Neurovirulence in Mice
Source: PLoS Pathog. 2014 Jul 31;10(7):e1004290. doi: 10.1371/journal.ppat.1004290 (PMC4117607; doi:10.1371/journal.ppat.1004290)
Supplement: Figure S2 — Viral growth properties of SA14-14-2MCV in SH-SY5Y and C6/36 cells. Cells were infected at an MOI of 1 with the molecularly cloned virus (SA14-14-2MCV) rescued from the full-length infectious cDNA or the original parental virus (SA14-14-2) used for cDNA construction. Culture supernatants were collected at the hour postinfection (hpi) indicated, and virus titers were determined by plaque assays on BHK-21 cells. (PPT) [file ppat.1004290.s002.ppt]

## Slide 1
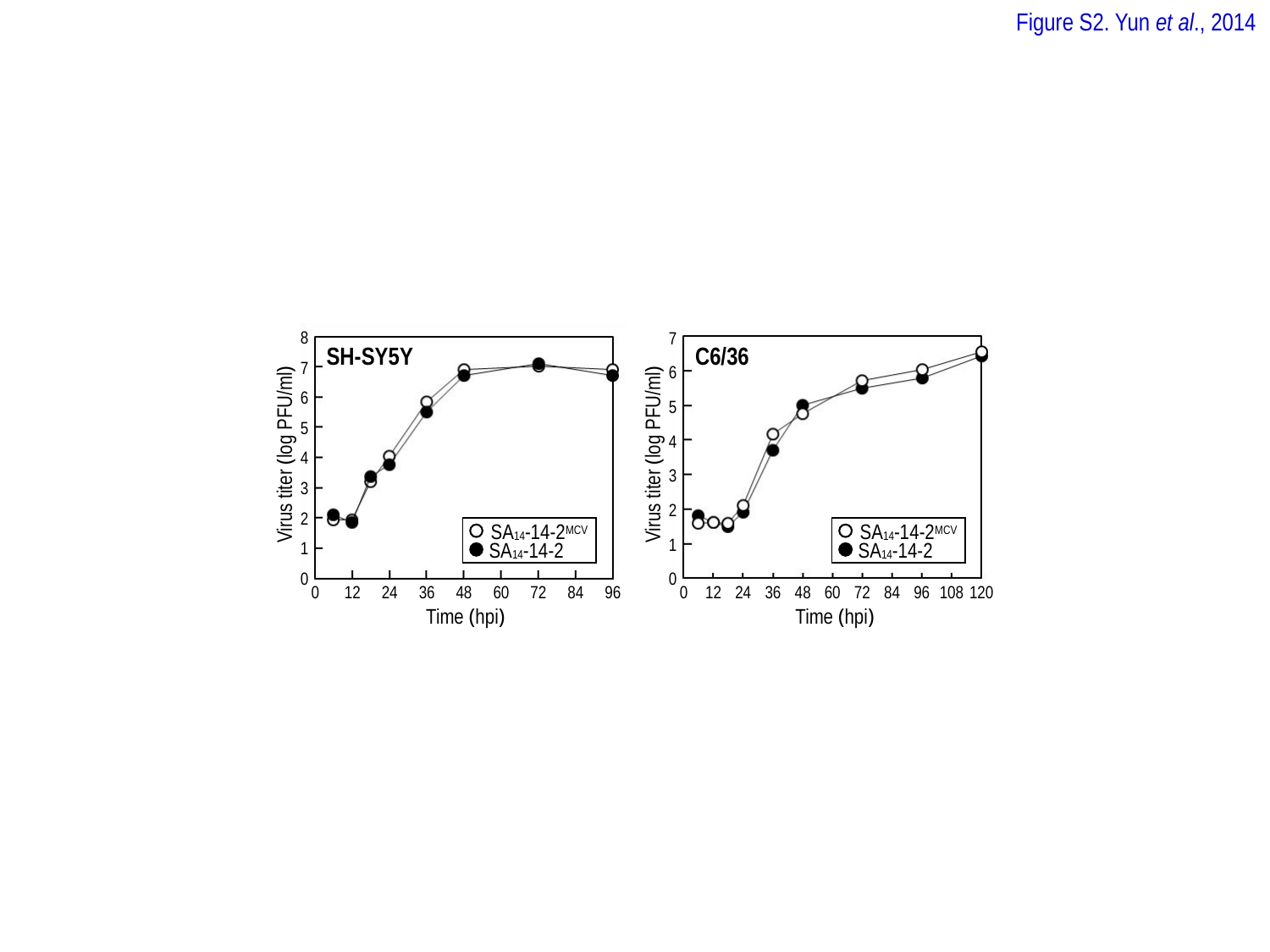

Figure S2. Yun et al., 2014
8
7
6
5
4
3
2
1
0
7
6
5
4
3
2
1
0
SH-SY5Y
C6/36
Virus titer (log PFU/ml)
Virus titer (log PFU/ml)
SA14-14-2MCV
SA14-14-2
SA14-14-2MCV
SA14-14-2
0
12
24
36
48
60
72
84
96
0
12
24
36
48
60
72
84
96
108
120
Time (hpi)
Time (hpi)
