# Supplementary material for: A Molecularly Cloned, Live-Attenuated Japanese Encephalitis Vaccine SA14-14-2 Virus: A Conserved Single Amino Acid in the ij Hairpin of the Viral E Glycoprotein Determines Neurovirulence in Mice
Source: PLoS Pathog. 2014 Jul 31;10(7):e1004290. doi: 10.1371/journal.ppat.1004290 (PMC4117607; doi:10.1371/journal.ppat.1004290)
Supplement: Figure S3 — Virological properties of SA14-14-2MCV in mice. Groups of 3-week-old female ICR mice (n = 20 per group) were mock-inoculated or inoculated intracerebrally (IC), intramuscularly (IM), or intraperitoneally (IP) with serial 10-fold dilutions of SA14-14-2MCV, SA14-14-2, or CNU/LP2 (a virulent JEV strain used as a reference). Mice were observed for any JEV-induced clinical signs and death every 12 h for 24 days. Survival curves were plotted by the Kaplan-Meier method. (PPT) [file ppat.1004290.s003.ppt]

## Slide 1
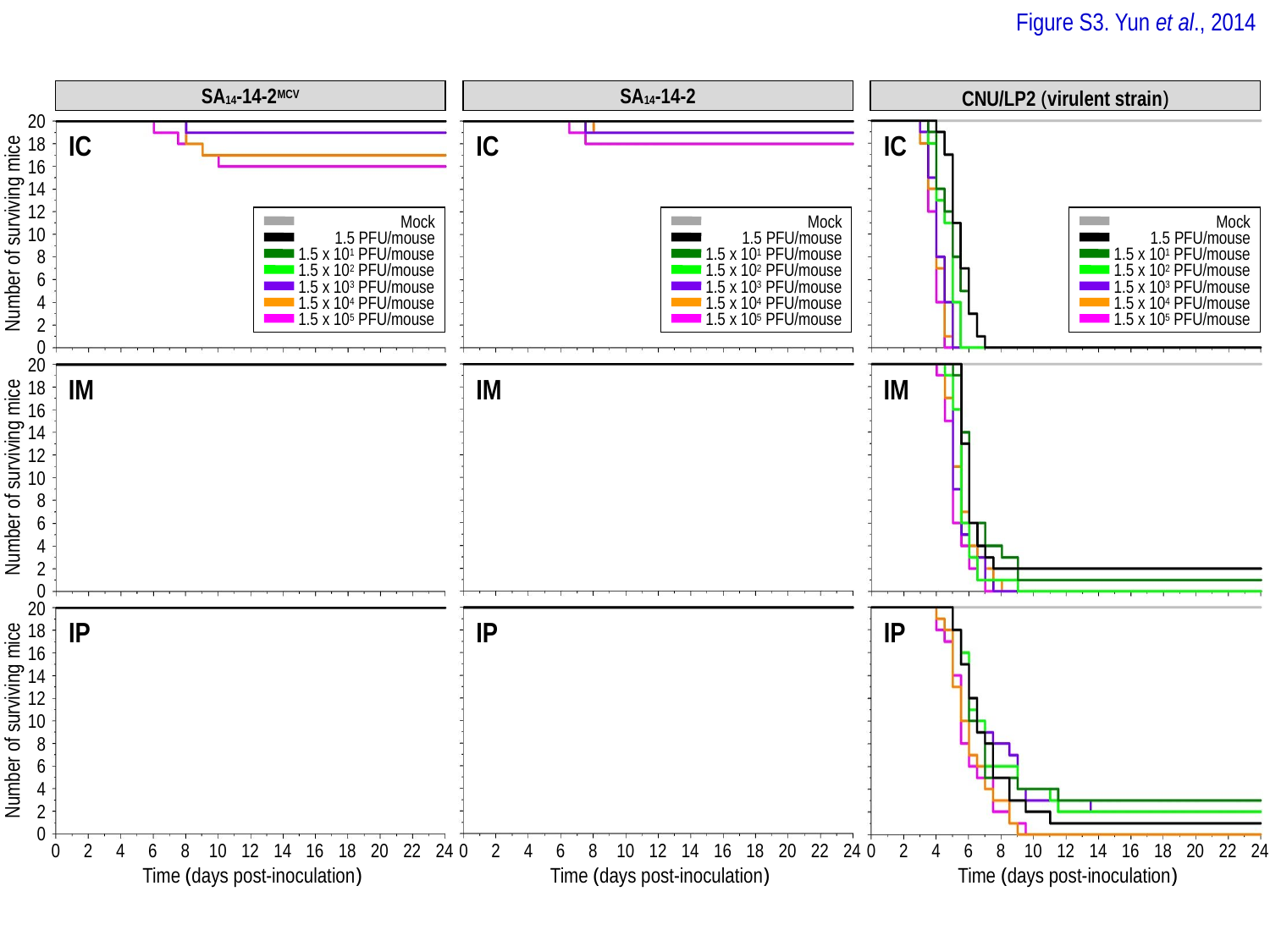

Figure S3. Yun et al., 2014
SA14-14-2MCV
SA14-14-2
CNU/LP2 (virulent strain)
20
18
16
14
12
10
8
6
4
2
0
IC
IC
IC
Mock
1.5 PFU/mouse
1.5 x 101 PFU/mouse
1.5 x 10^2^ PFU/mouse
1.5 x 10^3^ PFU/mouse
1.5 x 10^4^ PFU/mouse
1.5 x 10^5^ PFU/mouse
Mock
1.5 PFU/mouse
1.5 x 101 PFU/mouse
1.5 x 10^2^ PFU/mouse
1.5 x 10^3^ PFU/mouse
1.5 x 10^4^ PFU/mouse
1.5 x 10^5^ PFU/mouse
Mock
1.5 PFU/mouse
1.5 x 101 PFU/mouse
1.5 x 10^2^ PFU/mouse
1.5 x 10^3^ PFU/mouse
1.5 x 10^4^ PFU/mouse
1.5 x 10^5^ PFU/mouse
Number of surviving mice
20
18
16
14
12
10
8
6
4
2
0
IM
IM
IM
Number of surviving mice
20
18
16
14
12
10
8
6
4
2
0
IP
IP
IP
Number of surviving mice
0
2
4
6
8
10
12
14
16
18
20
22
24
Time (days post-inoculation)
0
2
4
6
8
10
12
14
16
18
20
22
24
Time (days post-inoculation)
0
2
4
6
8
10
12
14
16
18
20
22
24
Time (days post-inoculation)
