# Supplementary material for: A Molecularly Cloned, Live-Attenuated Japanese Encephalitis Vaccine SA14-14-2 Virus: A Conserved Single Amino Acid in the ij Hairpin of the Viral E Glycoprotein Determines Neurovirulence in Mice
Source: PLoS Pathog. 2014 Jul 31;10(7):e1004290. doi: 10.1371/journal.ppat.1004290 (PMC4117607; doi:10.1371/journal.ppat.1004290)
Supplement: Figure S4 — A single point mutation promotes susceptibility to SA14-14-2MCV infection of neurons in the CNS. Groups of 3-week-old female ICR mice (n = 15 per group) were mock-infected or infected IC with 103 PFU of SA14-14-2MCV (Parent), G1708A, or CNU/LP2 (a virulent JEV strain used as a reference). On the indicated days after infection, five mice were subjected for immunostaining of JEV NS1 antigen in fixed brain slices with an α-NS1 antiserum. Presented are representative slides of amygdala, cerebral cortex, thalamus, hypothalamus, and brainstem (note that hippocampal slides are shown in Fig. 4E). Arrowheads indicate the NS1-positive cells. (PPT) [file ppat.1004290.s004.ppt]

## Slide 1
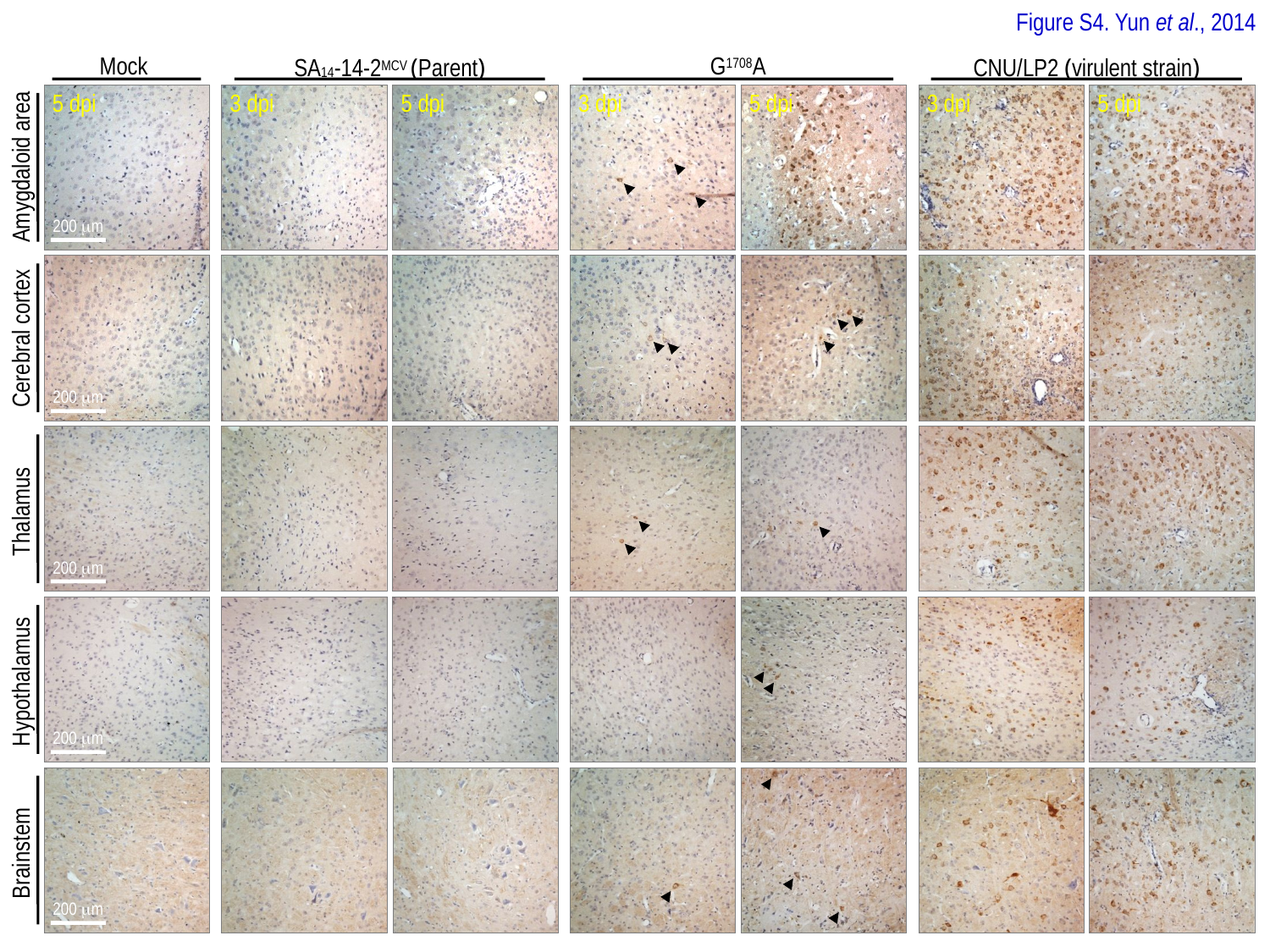

Figure S4. Yun et al., 2014
Mock
G1708A
SA14-14-2MCV (Parent)
CNU/LP2 (virulent strain)
5 dpi
3 dpi
5 dpi
3 dpi
5 dpi
3 dpi
5 dpi
Amygdaloid area
200 μm
Cerebral cortex
200 μm
Thalamus
200 μm
Hypothalamus
200 μm
Brainstem
200 μm
