# Supplementary material for: A Molecularly Cloned, Live-Attenuated Japanese Encephalitis Vaccine SA14-14-2 Virus: A Conserved Single Amino Acid in the ij Hairpin of the Viral E Glycoprotein Determines Neurovirulence in Mice
Source: PLoS Pathog. 2014 Jul 31;10(7):e1004290. doi: 10.1371/journal.ppat.1004290 (PMC4117607; doi:10.1371/journal.ppat.1004290)
Supplement: Figure S5 — Levels of JEV protein accumulation in BHK-21 cells transfected with 14 E-244 mutant RNAs. BHK-21 cells were mock-transfected or transfected with RNAs transcribed from SA14-14-2MCV (Parent) or each of the 14 E-244 mutant cDNAs as indicated. At 18 hpt, viral protein accumulation was analyzed by immunoblotting of cell lysates with a panel of JEV-specific antisera. In parallel, GAPDH protein was used as a loading and transfer control. (PPT) [file ppat.1004290.s005.ppt]

## Slide 1
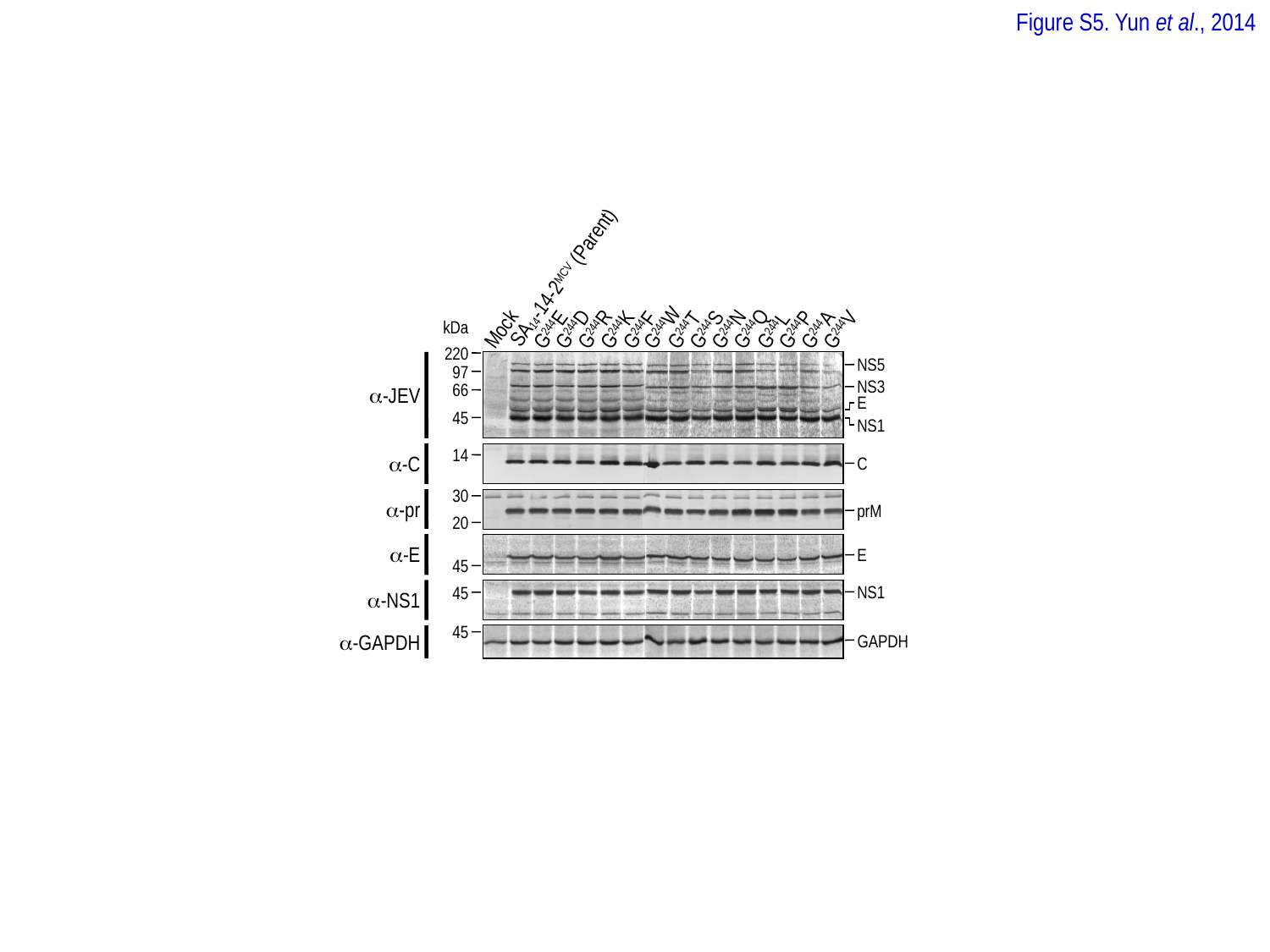

Figure S5. Yun et al., 2014
SA14-14-2MCV (Parent)
G244W
Mock
G244Q
G244D
G244R
G244N
G244E
G244K
G244S
G244P
G244A
G244V
G244F
G244T
G244L
kDa
220
97
66
45
14
30
20
45
45
45
NS5
NS3
E
NS1
C
prM
E
NS1
GAPDH
α-JEV
α-C
α-pr
α-E
α-NS1
α-GAPDH
