# Supplementary material for: A Molecularly Cloned, Live-Attenuated Japanese Encephalitis Vaccine SA14-14-2 Virus: A Conserved Single Amino Acid in the ij Hairpin of the Viral E Glycoprotein Determines Neurovirulence in Mice
Source: PLoS Pathog. 2014 Jul 31;10(7):e1004290. doi: 10.1371/journal.ppat.1004290 (PMC4117607; doi:10.1371/journal.ppat.1004290)
Supplement: Figure S7 — Amino acid sequence alignment of 154 fully sequenced JEV strains at the conserved ij hairpin of viral E glycoprotein. Multiple sequence alignments were performed using the amino acid sequence of 154 fully sequenced JEV genomes, including SA14 (red), SA14-14-2 (green), and two other SA14-derived attenuated strains, SA14-2-8 (orange) and SA14-12-1-7 (blue). Note that SA14 and SA14-14-2 have been sequenced by three and four independent research groups, respectively. The consensus sequence of the ij hairpin and its flanking region is presented on top, and only differences from that sequence are shown. Highlighted are the ∼15-aa ij-hairpin and the position E-244 in that hairpin. (PPT) [file ppat.1004290.s007.ppt]

## Slide 1
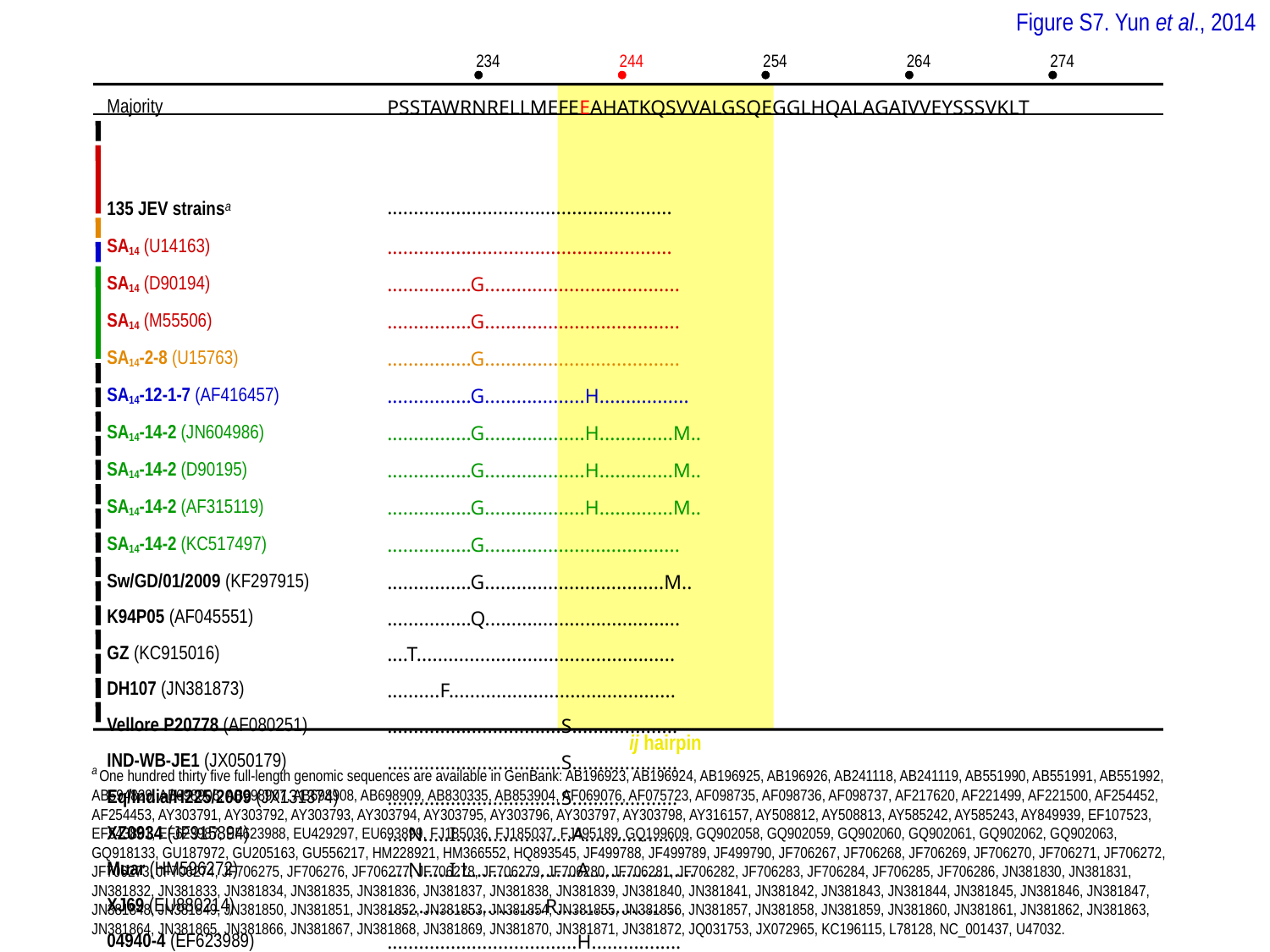

Figure S7. Yun et al., 2014
234
244
254
264
274
| Majority | PSSTAWRNRELLMEFEEAHATKQSVVALGSQEGGLHQALAGAIVVEYSSSVKLT |
| --- | --- |
| | |
| 135 JEV strainsa | ...................................................... |
| SA14 (U14163) | ...................................................... |
| SA14 (D90194) | ................G..................................... |
| SA14 (M55506) | ................G..................................... |
| SA14-2-8 (U15763) | ................G..................................... |
| SA14-12-1-7 (AF416457) | ................G...................H................. |
| SA14-14-2 (JN604986) | ................G...................H..............M.. |
| SA14-14-2 (D90195) | ................G...................H..............M.. |
| SA14-14-2 (AF315119) | ................G...................H..............M.. |
| SA14-14-2 (KC517497) | ................G..................................... |
| Sw/GD/01/2009 (KF297915) | ................G..................................M.. |
| K94P05 (AF045551) | ................Q..................................... |
| GZ (KC915016) | ....T................................................. |
| DH107 (JN381873) | ..........F........................................... |
| Vellore P20778 (AF080251) | .................................S.................... |
| IND-WB-JE1 (JX050179) | .................................S.................... |
| Eq/India/H225/2009 (JX131374) | .................................S.................... |
| XZ0934 (JF915894) | ....N.....I......................A.................... |
| Muar (HM596272) | ....N.....I.L....................A.................... |
| XJ69 (EU880214) | ..............................R....................... |
| 04940-4 (EF623989) | ....................................H................. |
| Nakayama (EF571853) | ................................................N..... |
| GD (JN711458) | ................................................N..... |
| HN2 (JN711459) | ................................................N..... |
| Sw/Mie/51/2006 (AB698905) | .....................................................A |
ij hairpin
a One hundred thirty five full-length genomic sequences are available in GenBank: AB196923, AB196924, AB196925, AB196926, AB241118, AB241119, AB551990, AB551991, AB551992, AB594829, AB698906, AB698907, AB698908, AB698909, AB830335, AB853904, AF069076, AF075723, AF098735, AF098736, AF098737, AF217620, AF221499, AF221500, AF254452,
AF254453, AY303791, AY303792, AY303793, AY303794, AY303795, AY303796, AY303797, AY303798, AY316157, AY508812, AY508813, AY585242, AY585243, AY849939, EF107523, EF543861, EF623987, EF623988, EU429297, EU693899, FJ185036, FJ185037, FJ495189, GQ199609, GQ902058, GQ902059, GQ902060, GQ902061, GQ902062, GQ902063,
GQ918133, GU187972, GU205163, GU556217, HM228921, HM366552, HQ893545, JF499788, JF499789, JF499790, JF706267, JF706268, JF706269, JF706270, JF706271, JF706272,
JF706273, JF706274, JF706275, JF706276, JF706277, JF706278, JF706279, JF706280, JF706281, JF706282, JF706283, JF706284, JF706285, JF706286, JN381830, JN381831,
JN381832, JN381833, JN381834, JN381835, JN381836, JN381837, JN381838, JN381839, JN381840, JN381841, JN381842, JN381843, JN381844, JN381845, JN381846, JN381847,
JN381848, JN381849, JN381850, JN381851, JN381852, JN381853, JN381854, JN381855, JN381856, JN381857, JN381858, JN381859, JN381860, JN381861, JN381862, JN381863,
JN381864, JN381865, JN381866, JN381867, JN381868, JN381869, JN381870, JN381871, JN381872, JQ031753, JX072965, KC196115, L78128, NC_001437, U47032.
