# Supplementary material for: A Molecularly Cloned, Live-Attenuated Japanese Encephalitis Vaccine SA14-14-2 Virus: A Conserved Single Amino Acid in the ij Hairpin of the Viral E Glycoprotein Determines Neurovirulence in Mice
Source: PLoS Pathog. 2014 Jul 31;10(7):e1004290. doi: 10.1371/journal.ppat.1004290 (PMC4117607; doi:10.1371/journal.ppat.1004290)
Supplement: Table S2 — Neurovirulence of SA14-14-2MCV and its eight mutants in 3-week-old ICR mice. (PPT) [file ppat.1004290.s009.ppt]

## Slide 1
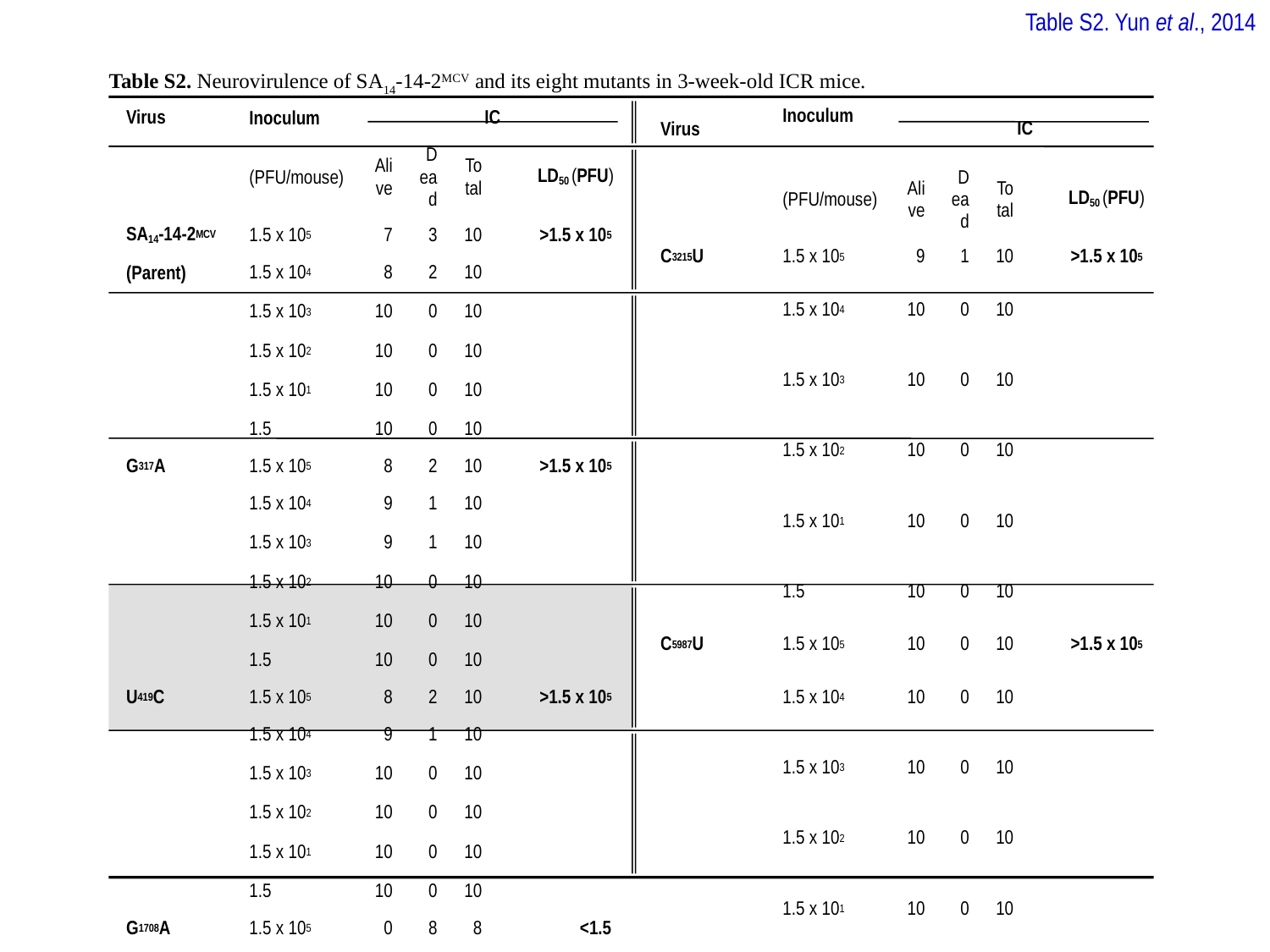

Table S2. Yun et al., 2014
Table S2. Neurovirulence of SA14-14-2MCV and its eight mutants in 3-week-old ICR mice.
| Virus | Inoculum | IC | | | |
| --- | --- | --- | --- | --- | --- |
| | (PFU/mouse) | Alive | Dead | Total | LD50 (PFU) |
| SA14-14-2MCV | 1.5 x 10^5^ | 7 | 3 | 10 | >1.5 x 10^5^ |
| (Parent) | 1.5 x 10^4^ | 8 | 2 | 10 | |
| | 1.5 x 10^3^ | 10 | 0 | 10 | |
| | 1.5 x 10^2^ | 10 | 0 | 10 | |
| | 1.5 x 101 | 10 | 0 | 10 | |
| | 1.5 | 10 | 0 | 10 | |
| G317A | 1.5 x 10^5^ | 8 | 2 | 10 | >1.5 x 10^5^ |
| | 1.5 x 10^4^ | 9 | 1 | 10 | |
| | 1.5 x 10^3^ | 9 | 1 | 10 | |
| | 1.5 x 10^2^ | 10 | 0 | 10 | |
| | 1.5 x 101 | 10 | 0 | 10 | |
| | 1.5 | 10 | 0 | 10 | |
| U419C | 1.5 x 10^5^ | 8 | 2 | 10 | >1.5 x 10^5^ |
| | 1.5 x 10^4^ | 9 | 1 | 10 | |
| | 1.5 x 10^3^ | 10 | 0 | 10 | |
| | 1.5 x 10^2^ | 10 | 0 | 10 | |
| | 1.5 x 101 | 10 | 0 | 10 | |
| | 1.5 | 10 | 0 | 10 | |
| G1708A | 1.5 x 10^5^ | 0 | 8 | 8 | <1.5 |
| | 1.5 x 10^4^ | 0 | 10 | 10 | |
| | 1.5 x 10^3^ | 0 | 10 | 10 | |
| | 1.5 x 10^2^ | 0 | 10 | 10 | |
| | 1.5 x 101 | 0 | 10 | 10 | |
| | 1.5 | 0 | 10 | 10 | |
| U2580C | 1.5 x 10^5^ | 8 | 2 | 10 | >1.5 x 10^5^ |
| | 1.5 x 10^4^ | 10 | 0 | 10 | |
| | 1.5 x 10^3^ | 10 | 0 | 10 | |
| | 1.5 x 10^2^ | 10 | 0 | 10 | |
| | 1.5 x 101 | 10 | 0 | 10 | |
| | 1.5 | 10 | 0 | 10 | |
| Virus | Inoculum | IC | | | |
| --- | --- | --- | --- | --- | --- |
| | (PFU/mouse) | Alive | Dead | Total | LD50 (PFU) |
| C3215U | 1.5 x 10^5^ | 9 | 1 | 10 | >1.5 x 10^5^ |
| | 1.5 x 10^4^ | 10 | 0 | 10 | |
| | 1.5 x 10^3^ | 10 | 0 | 10 | |
| | 1.5 x 10^2^ | 10 | 0 | 10 | |
| | 1.5 x 101 | 10 | 0 | 10 | |
| | 1.5 | 10 | 0 | 10 | |
| C5987U | 1.5 x 10^5^ | 10 | 0 | 10 | >1.5 x 10^5^ |
| | 1.5 x 10^4^ | 10 | 0 | 10 | |
| | 1.5 x 10^3^ | 10 | 0 | 10 | |
| | 1.5 x 10^2^ | 10 | 0 | 10 | |
| | 1.5 x 101 | 10 | 0 | 10 | |
| | 1.5 | 10 | 0 | 10 | |
| G6551A | 1.5 x 10^5^ | 10 | 0 | 10 | >1.5 x 10^5^ |
| | 1.5 x 10^4^ | 10 | 0 | 10 | |
| | 1.5 x 10^3^ | 10 | 0 | 10 | |
| | 1.5 x 10^2^ | 10 | 0 | 10 | |
| | 1.5 x 101 | 10 | 0 | 10 | |
| | 1.5 | 10 | 0 | 10 | |
| U8588C | 1.5 x 10^5^ | 10 | 0 | 10 | >1.5 x 10^5^ |
| | 1.5 x 10^4^ | 9 | 1 | 10 | |
| | 1.5 x 10^3^ | 10 | 0 | 10 | |
| | 1.5 x 10^2^ | 10 | 0 | 10 | |
| | 1.5 x 101 | 10 | 0 | 10 | |
| | 1.5 | 10 | 0 | 10 | |
| | | | | | |
| | | | | | |
| | | | | | |
| | | | | | |
| | | | | | |
| | | | | | |
