# Supplementary material for: A Molecularly Cloned, Live-Attenuated Japanese Encephalitis Vaccine SA14-14-2 Virus: A Conserved Single Amino Acid in the ij Hairpin of the Viral E Glycoprotein Determines Neurovirulence in Mice
Source: PLoS Pathog. 2014 Jul 31;10(7):e1004290. doi: 10.1371/journal.ppat.1004290 (PMC4117607; doi:10.1371/journal.ppat.1004290)
Supplement: Table S3 — Neurovirulence of SA14-14-2MCV and its 14 E-244 mutants in 3-week-old ICR mice. (PPT) [file ppat.1004290.s010.ppt]

## Slide 1
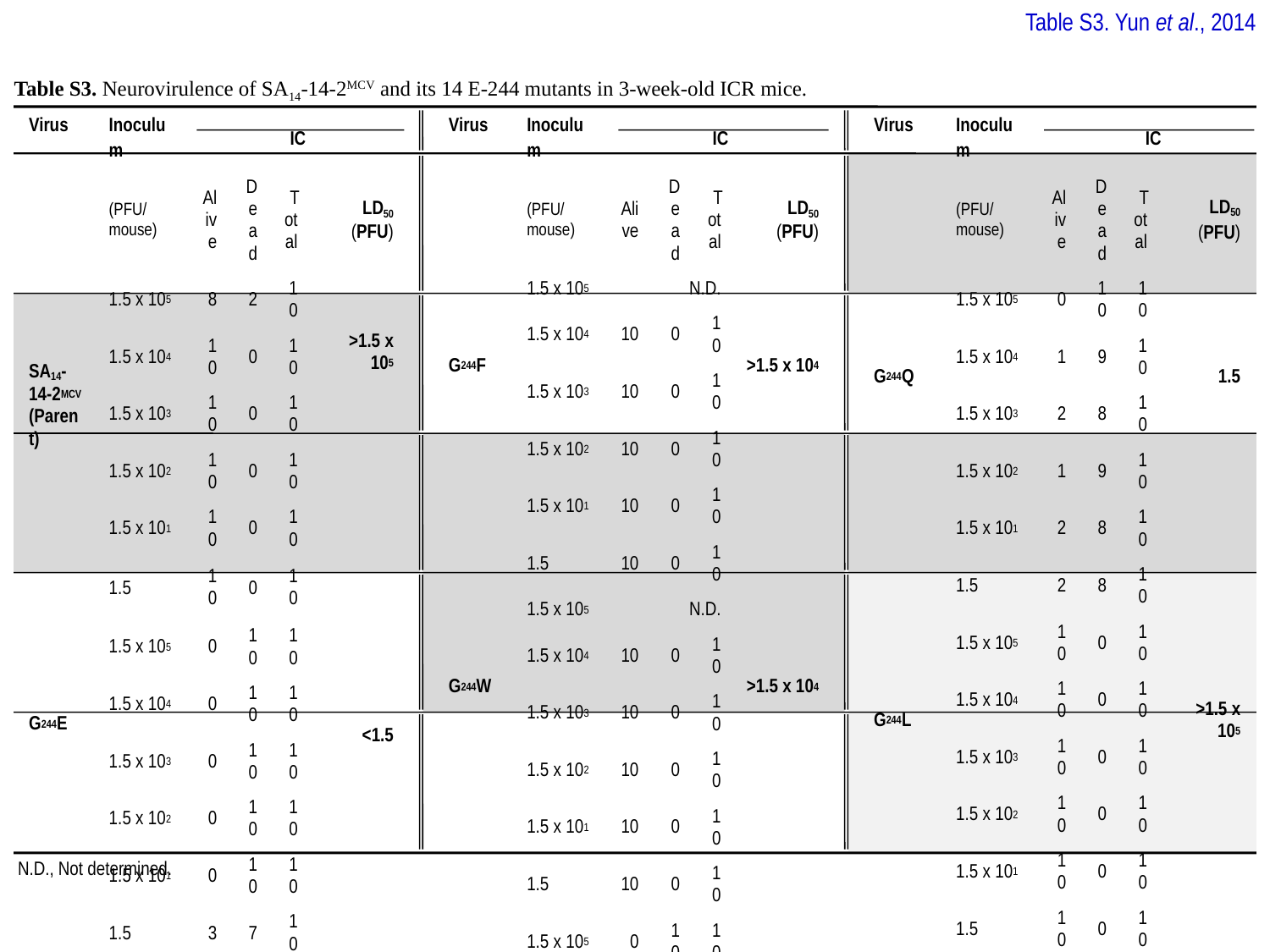

Table S3. Yun et al., 2014
Table S3. Neurovirulence of SA14-14-2MCV and its 14 E-244 mutants in 3-week-old ICR mice.
| Virus | Inoculum | IC | | | |
| --- | --- | --- | --- | --- | --- |
| | (PFU/mouse) | Alive | Dead | Total | LD50 (PFU) |
| SA14-14-2MCV (Parent) | 1.5 x 10^5^ | 8 | 2 | 10 | >1.5 x 10^5^ |
| | 1.5 x 10^4^ | 10 | 0 | 10 | |
| | 1.5 x 10^3^ | 10 | 0 | 10 | |
| | 1.5 x 10^2^ | 10 | 0 | 10 | |
| | 1.5 x 101 | 10 | 0 | 10 | |
| | 1.5 | 10 | 0 | 10 | |
| G244E | 1.5 x 10^5^ | 0 | 10 | 10 | <1.5 |
| | 1.5 x 10^4^ | 0 | 10 | 10 | |
| | 1.5 x 10^3^ | 0 | 10 | 10 | |
| | 1.5 x 10^2^ | 0 | 10 | 10 | |
| | 1.5 x 101 | 0 | 10 | 10 | |
| | 1.5 | 3 | 7 | 10 | |
| G244D | 1.5 x 10^5^ | 0 | 10 | 10 | 1.5 |
| | 1.5 x 10^4^ | 0 | 10 | 10 | |
| | 1.5 x 10^3^ | 0 | 10 | 10 | |
| | 1.5 x 10^2^ | 0 | 10 | 10 | |
| | 1.5 x 101 | 2 | 8 | 10 | |
| | 1.5 | 4 | 6 | 10 | |
| G244R | 1.5 x 10^5^ | N.D. | | | >1.5 x 10^4^ |
| | 1.5 x 10^4^ | 10 | 0 | 10 | |
| | 1.5 x 10^3^ | 10 | 0 | 10 | |
| | 1.5 x 10^2^ | 10 | 0 | 10 | |
| | 1.5 x 101 | 10 | 0 | 10 | |
| | 1.5 | 10 | 0 | 10 | |
| G244K | 1.5 x 10^5^ | N.D. | | | >1.5 x 10^4^ |
| | 1.5 x 10^4^ | 7 | 3 | 10 | |
| | 1.5 x 10^3^ | 9 | 1 | 10 | |
| | 1.5 x 10^2^ | 10 | 0 | 10 | |
| | 1.5 x 101 | 10 | 0 | 10 | |
| | 1.5 | 10 | 0 | 10 | |
| Virus | Inoculum | IC | | | |
| --- | --- | --- | --- | --- | --- |
| | (PFU/mouse) | Alive | Dead | Total | LD50 (PFU) |
| G244F | 1.5 x 10^5^ | N.D. | | | >1.5 x 10^4^ |
| | 1.5 x 10^4^ | 10 | 0 | 10 | |
| | 1.5 x 10^3^ | 10 | 0 | 10 | |
| | 1.5 x 10^2^ | 10 | 0 | 10 | |
| | 1.5 x 101 | 10 | 0 | 10 | |
| | 1.5 | 10 | 0 | 10 | |
| G244W | 1.5 x 10^5^ | N.D. | | | >1.5 x 10^4^ |
| | 1.5 x 10^4^ | 10 | 0 | 10 | |
| | 1.5 x 10^3^ | 10 | 0 | 10 | |
| | 1.5 x 10^2^ | 10 | 0 | 10 | |
| | 1.5 x 101 | 10 | 0 | 10 | |
| | 1.5 | 10 | 0 | 10 | |
| G244T | 1.5 x 10^5^ | 0 | 10 | 10 | <1.5 |
| | 1.5 x 10^4^ | 0 | 10 | 10 | |
| | 1.5 x 10^3^ | 0 | 10 | 10 | |
| | 1.5 x 10^2^ | 0 | 10 | 10 | |
| | 1.5 x 101 | 2 | 8 | 10 | |
| | 1.5 | 3 | 7 | 10 | |
| G244S | 1.5 x 10^5^ | 0 | 10 | 10 | 3.1 x 101 |
| | 1.5 x 10^4^ | 1 | 9 | 10 | |
| | 1.5 x 10^3^ | 1 | 9 | 10 | |
| | 1.5 x 10^2^ | 3 | 7 | 10 | |
| | 1.5 x 101 | 5 | 5 | 10 | |
| | 1.5 | 9 | 1 | 10 | |
| G244N | 1.5 x 10^5^ | 6 | 4 | 10 | >1.5 x 10^5^ |
| | 1.5 x 10^4^ | 9 | 1 | 10 | |
| | 1.5 x 10^3^ | 10 | 0 | 10 | |
| | 1.5 x 10^2^ | 10 | 0 | 10 | |
| | 1.5 x 101 | 10 | 0 | 10 | |
| | 1.5 | 10 | 0 | 10 | |
| Virus | Inoculum | IC | | | |
| --- | --- | --- | --- | --- | --- |
| | (PFU/mouse) | Alive | Dead | Total | LD50 (PFU) |
| G244Q | 1.5 x 10^5^ | 0 | 10 | 10 | 1.5 |
| | 1.5 x 10^4^ | 1 | 9 | 10 | |
| | 1.5 x 10^3^ | 2 | 8 | 10 | |
| | 1.5 x 10^2^ | 1 | 9 | 10 | |
| | 1.5 x 101 | 2 | 8 | 10 | |
| | 1.5 | 2 | 8 | 10 | |
| G244L | 1.5 x 10^5^ | 10 | 0 | 10 | >1.5 x 10^5^ |
| | 1.5 x 10^4^ | 10 | 0 | 10 | |
| | 1.5 x 10^3^ | 10 | 0 | 10 | |
| | 1.5 x 10^2^ | 10 | 0 | 10 | |
| | 1.5 x 101 | 10 | 0 | 10 | |
| | 1.5 | 10 | 0 | 10 | |
| G244P | 1.5 x 10^5^ | 0 | 10 | 10 | <1.5 |
| | 1.5 x 10^4^ | 0 | 10 | 10 | |
| | 1.5 x 10^3^ | 0 | 10 | 10 | |
| | 1.5 x 10^2^ | 0 | 10 | 10 | |
| | 1.5 x 101 | 0 | 10 | 10 | |
| | 1.5 | 0 | 10 | 10 | |
| G244A | 1.5 x 10^5^ | 1 | 9 | 10 | 5.8 x 10^3^ |
| | 1.5 x 10^4^ | 4 | 6 | 10 | |
| | 1.5 x 10^3^ | 6 | 4 | 10 | |
| | 1.5 x 10^2^ | 8 | 2 | 10 | |
| | 1.5 x 101 | 10 | 0 | 10 | |
| | 1.5 | 10 | 0 | 10 | |
| G244V | 1.5 x 10^5^ | 0 | 10 | 10 | 1.2 x 10^3^ |
| | 1.5 x 10^4^ | 3 | 7 | 10 | |
| | 1.5 x 10^3^ | 3 | 7 | 10 | |
| | 1.5 x 10^2^ | 4 | 6 | 10 | |
| | 1.5 x 101 | 8 | 2 | 10 | |
| | 1.5 | 9 | 1 | 10 | |
N.D., Not determined.
