# Supplementary material for: A Molecularly Cloned, Live-Attenuated Japanese Encephalitis Vaccine SA14-14-2 Virus: A Conserved Single Amino Acid in the ij Hairpin of the Viral E Glycoprotein Determines Neurovirulence in Mice
Source: PLoS Pathog. 2014 Jul 31;10(7):e1004290. doi: 10.1371/journal.ppat.1004290 (PMC4117607; doi:10.1371/journal.ppat.1004290)
Supplement: Table S4 — Oligonucleotides used for ligation, cDNA synthesis, and PCR amplification. (PPT) [file ppat.1004290.s011.ppt]

## Slide 1
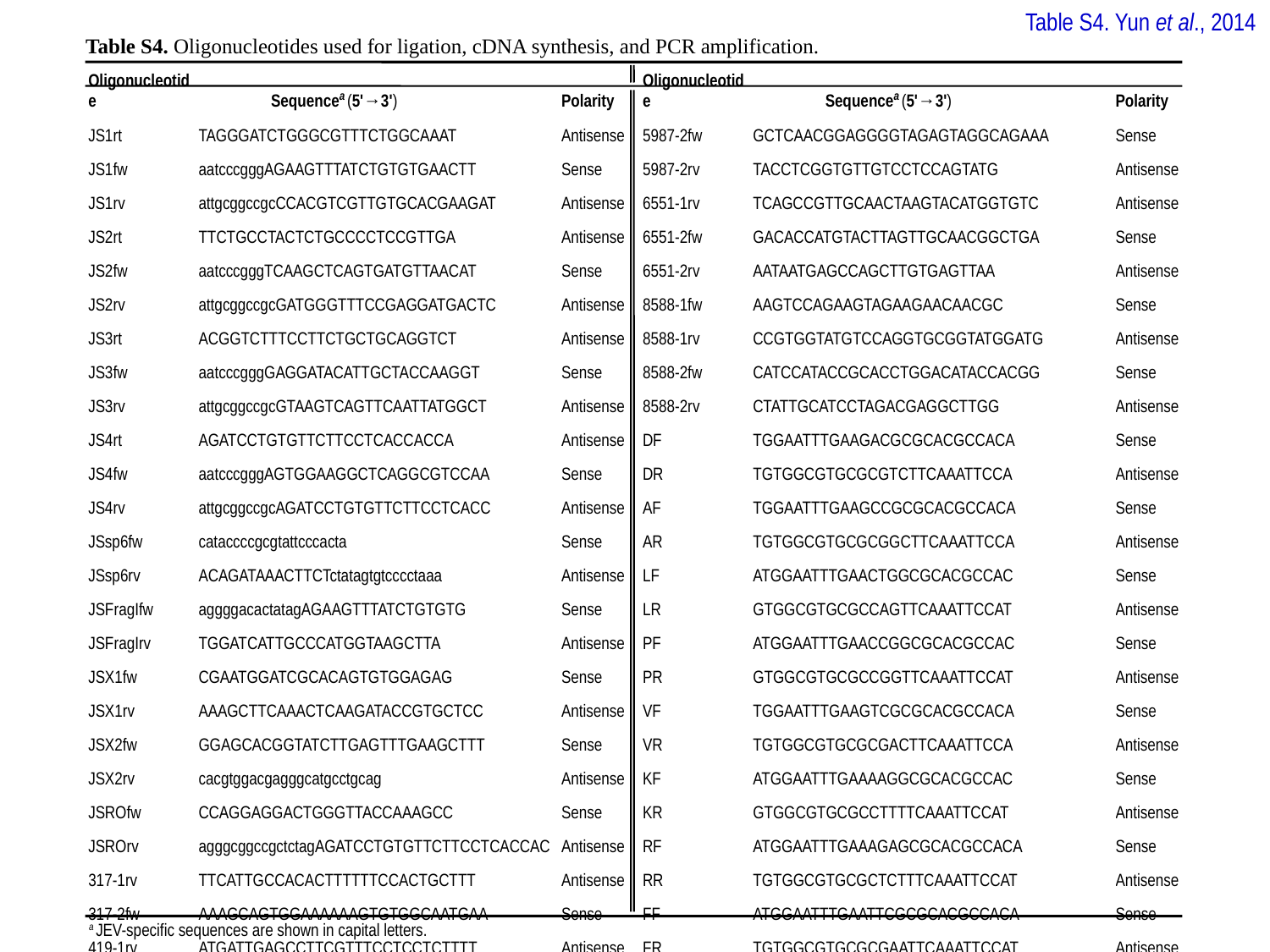

Table S4. Yun et al., 2014
Table S4. Oligonucleotides used for ligation, cDNA synthesis, and PCR amplification.
| Oligonucleotide | Sequencea (5'→3') | Polarity |
| --- | --- | --- |
| JS1rt | TAGGGATCTGGGCGTTTCTGGCAAAT | Antisense |
| JS1fw | aatcccgggAGAAGTTTATCTGTGTGAACTT | Sense |
| JS1rv | attgcggccgcCCACGTCGTTGTGCACGAAGAT | Antisense |
| JS2rt | TTCTGCCTACTCTGCCCCTCCGTTGA | Antisense |
| JS2fw | aatcccgggTCAAGCTCAGTGATGTTAACAT | Sense |
| JS2rv | attgcggccgcGATGGGTTTCCGAGGATGACTC | Antisense |
| JS3rt | ACGGTCTTTCCTTCTGCTGCAGGTCT | Antisense |
| JS3fw | aatcccgggGAGGATACATTGCTACCAAGGT | Sense |
| JS3rv | attgcggccgcGTAAGTCAGTTCAATTATGGCT | Antisense |
| JS4rt | AGATCCTGTGTTCTTCCTCACCACCA | Antisense |
| JS4fw | aatcccgggAGTGGAAGGCTCAGGCGTCCAA | Sense |
| JS4rv | attgcggccgcAGATCCTGTGTTCTTCCTCACC | Antisense |
| JSsp6fw | cataccccgcgtattcccacta | Sense |
| JSsp6rv | ACAGATAAACTTCTctatagtgtcccctaaa | Antisense |
| JSFragIfw | aggggacactatagAGAAGTTTATCTGTGTG | Sense |
| JSFragIrv | TGGATCATTGCCCATGGTAAGCTTA | Antisense |
| JSX1fw | CGAATGGATCGCACAGTGTGGAGAG | Sense |
| JSX1rv | AAAGCTTCAAACTCAAGATACCGTGCTCC | Antisense |
| JSX2fw | GGAGCACGGTATCTTGAGTTTGAAGCTTT | Sense |
| JSX2rv | cacgtggacgagggcatgcctgcag | Antisense |
| JSROfw | CCAGGAGGACTGGGTTACCAAAGCC | Sense |
| JSROrv | agggcggccgctctagAGATCCTGTGTTCTTCCTCACCAC | Antisense |
| 317-1rv | TTCATTGCCACACTTTTTTCCACTGCTTT | Antisense |
| 317-2fw | AAAGCAGTGGAAAAAAGTGTGGCAATGAA | Sense |
| 419-1rv | ATGATTGAGCCTTCGTTTCCTCCTCTTTT | Antisense |
| 419-2fw | AAAAGAGGAGGAAACGAAGGCTCAATCAT | Sense |
| 1708-1fw | ACATTGGACGTCCGCATGATTAACA | Sense |
| 1708-1rv | TTGTGGCGTGCGCCTCTTCAAATTCCATG | Antisense |
| 1708-2fw | CATGGAATTTGAAGAGGCGCACGCCACAA | Sense |
| 1708-2rv | GATTTTTCGCGAACGAGAATTTTTC | Antisense |
| JSBsrGIfw | CTGGCTCTGAAAGGCACAACCTATG | Sense |
| 2580-1rv | GGGCGTTTCTGGCAGATATTTATACCTAT | Antisense |
| 2580-2fw | ATAGGTATAAATATCTGCCAGAAACGCCC | Sense |
| JSBamHIrv | TCATGGATTGGGGCATTTGAGTCAG | Antisense |
| 3215-1rv | TTTGGTCCGGCTATAGTGTGCGGAATGAT | Antisense |
| 3215-2fw | ATCATTCCGCACACTATAGCCGGACCAAA | Sense |
| JSBamHIfw | CTTTATGACAGCGACCCCGCCTGGA | Sense |
| 5987-1rv | TTTCTGCCTACTCTACCCCTCCGTTGAGC | Antisense |
| Oligonucleotide | Sequencea (5'→3') | Polarity |
| --- | --- | --- |
| 5987-2fw | GCTCAACGGAGGGGTAGAGTAGGCAGAAA | Sense |
| 5987-2rv | TACCTCGGTGTTGTCCTCCAGTATG | Antisense |
| 6551-1rv | TCAGCCGTTGCAACTAAGTACATGGTGTC | Antisense |
| 6551-2fw | GACACCATGTACTTAGTTGCAACGGCTGA | Sense |
| 6551-2rv | AATAATGAGCCAGCTTGTGAGTTAA | Antisense |
| 8588-1fw | AAGTCCAGAAGTAGAAGAACAACGC | Sense |
| 8588-1rv | CCGTGGTATGTCCAGGTGCGGTATGGATG | Antisense |
| 8588-2fw | CATCCATACCGCACCTGGACATACCACGG | Sense |
| 8588-2rv | CTATTGCATCCTAGACGAGGCTTGG | Antisense |
| DF | TGGAATTTGAAGACGCGCACGCCACA | Sense |
| DR | TGTGGCGTGCGCGTCTTCAAATTCCA | Antisense |
| AF | TGGAATTTGAAGCCGCGCACGCCACA | Sense |
| AR | TGTGGCGTGCGCGGCTTCAAATTCCA | Antisense |
| LF | ATGGAATTTGAACTGGCGCACGCCAC | Sense |
| LR | GTGGCGTGCGCCAGTTCAAATTCCAT | Antisense |
| PF | ATGGAATTTGAACCGGCGCACGCCAC | Sense |
| PR | GTGGCGTGCGCCGGTTCAAATTCCAT | Antisense |
| VF | TGGAATTTGAAGTCGCGCACGCCACA | Sense |
| VR | TGTGGCGTGCGCGACTTCAAATTCCA | Antisense |
| KF | ATGGAATTTGAAAAGGCGCACGCCAC | Sense |
| KR | GTGGCGTGCGCCTTTTCAAATTCCAT | Antisense |
| RF | ATGGAATTTGAAAGAGCGCACGCCACA | Sense |
| RR | TGTGGCGTGCGCTCTTTCAAATTCCAT | Antisense |
| FF | ATGGAATTTGAATTCGCGCACGCCACA | Sense |
| FR | TGTGGCGTGCGCGAATTCAAATTCCAT | Antisense |
| WF | ATGGAATTTGAATGGGCGCACGCCAC | Sense |
| WR | GTGGCGTGCGCCCATTCAAATTCCAT | Antisense |
| SF | ATGGAATTTGAAAGCGCGCACGCCACA | Sense |
| SR | TGTGGCGTGCGCGCTTTCAAATTCCAT | Antisense |
| TF | ATGGAATTTGAAACGGCGCACGCCAC | Sense |
| TR | GTGGCGTGCGCCGTTTCAAATTCCAT | Antisense |
| NF | ATGGAATTTGAAAACGCGCACGCCACA | Sense |
| NR | TGTGGCGTGCGCGTTTTCAAATTCCAT | Antisense |
| QF | ATGGAATTTGAACAGGCGCACGCCAC | Sense |
| QR | GTGGCGTGCGCCTGTTCAAATTCCAT | Antisense |
| prMErt | ATTTATACCTATCCACCCAGGCTTCC | Antisense |
| prMEfw | aatctcgagAGTTGTCATAGCTTGTGCAGG | Sense |
| prMErv | attccgcggTGATGTCAATGGCACATCCAG | Antisense |
a JEV-specific sequences are shown in capital letters.
