# Supplementary material for: Correction: A Molecularly Cloned, Live-Attenuated Japanese Encephalitis Vaccine SA14-14-2 Virus: A Conserved Single Amino Acid in the ij Hairpin of the Viral E Glycoprotein Determines Neurovirulence in Mice
Source: PLoS Pathog. 2014 Sep 22;10(9):e1004465. doi: 10.1371/journal.ppat.1004465 (PMC4171538; doi:10.1371/journal.ppat.1004465)
Supplement: Text S1 — Supplementary Materials and Methods. (PDF) [file ppat.1004465.s001.pdf]

[REVISED: PPATHOGENS-D-13-02269, VERSION 2]

[Supporting Information]

A Molecularly Cloned, Live-Attenuated Japanese Encephalitis Vaccine SA_{14}-14-2 Virus: A Conserved Single Amino Acid in the *ij* Hairpin of the Viral E Glycoprotein Determines Neurovirulence in Mice

Sang-Im Yun[1¶], Byung-Hak Song[1¶], Jin-Kyoung Kim[1], Gil-Nam Yun[2], Eun-Young Lee[3], Long Li[4], Richard J. Kuhn[4], Michael G. Rossmann[4], John D. Morrey[1], Young-Min Lee[1]*

1 Department of Animal, Dairy, and Veterinary Sciences; Utah Science Technology and Research, College of Agriculture and Applied Sciences, Utah State University, Logan, Utah, United States of America, **2** Department of Microbiology, College of Medicine, Chungbuk National University, Cheongju, Republic of Korea, **3** Department of Anatomy, College of Medicine, Chungbuk National University, Cheongju, Republic of Korea, **4** Department of Biological Sciences, Purdue University, West Lafayette, Indiana, United States of America

Short Title: JEV Neurovirulence

- **Manuscript:**
  Total Number of Text Pages: 46
  Total Number of Characters: 91,220 (with spaces)
  Total Number of Words in Abstract: 287
  Total Number of Tables: 1
  Total Number of Figures: 7
- **Supporting Information:**
  One Text File
  Total Number of Supplemental Tables: 4 (Tables S1 to S4)
  Total Number of Supplemental Figures: 7 (Figures S1 to S7)

¶ These authors contributed equally to this work.

* Corresponding author: UMC 9830, Old Main Hill, Logan, UT 84322-9830, USA. Phone: 435-797-9667. E-mail: youngmin.lee@usu.edu.

This article contains supporting information provided separately.

Materials and Methods

Construction of a full-length infectious cDNA of SA_{14}-14-2

All plasmids were constructed by standard recombinant DNA techniques [1]. The oligonucleotides used in this study are listed in Table S4. Using the SA_{14}-14-2 genomic RNA as a template, a contig of four cDNA fragments (Frag-I to IV) was first synthesized by RT-PCR using the following primers: Frag-I (2573 bp), JS1rt and JS1fw+JS1rv; Frag-II (4171 bp), JS2rt and JS2fw+JS2rv; Frag-III (3922 bp), JS3rt and JS3fw+JS3rv; and Frag-IV (1798 bp), JS4rt and JS4fw+JS4rv. The four overlapping cDNAs were individually subcloned into pBAC/PRRSV/FL [2], a derivative of the pBeloBAC11 plasmid, by ligating the 15426-bp *Pme*I-*Not*I fragment of pBAC/PRRSV/FL with the 2559-, 4157-, 3908-, and 1784-bp *Sma*I-*Not*I fragments of the Frag-I to IV amplicons, respectively. This created pBAC/Frag-I to IV.

To introduce the SP6 promoter immediately upstream of the 5'-end of the full-length SA_{14}-14-2 cDNA, the pBAC/Frag-I was modified. Two DNA fragments were first amplified by (*i*) PCR of $pBAC^{SP6}$/JVFLx/*Xba*I with primers JSsp6fw+JSsp6rv (JSsp6rv incorporates the antisense sequence of the SP6 promoter) and (*ii*) PCR of pBAC/Frag-I with primers JSFragIfw+JSFragIrv. These two fragments were then fused by a second round of PCR with primers JSsp6fw+JSFragIrv. The 760-bp *Pac*I-*Bsi*WI fragment of the fused PCR amplicons was ligated with the 9532-bp *Pac*I-*Bsi*WI fragment of pBAC/Frag-I. This generated pBAC/Frag-I^{SP6}.

To engineer an artificial *Xba*I run-off site immediately downstream of the 3'-end of the full-length SA_{14}-14-2 cDNA, the pre-existing, internal *Xba*I site at position 9131 in the NS5 protein-coding region was first removed by introducing a silent point mutation, $A^{9134}\rightarrow T$. Thus, two DNA fragments were generated by PCR of pBAC/Frag-III with two pairs of primers, JSX1fw+JSX1rv and JSX2fw+JSX2rv. These two fragments were then fused by a second round of PCR with primers JSX1fw+JSX2rv. The 949-bp *Avr*II-*Not*I fragment of the fused amplicons was ligated with the 16245-

bp *Not*I-*Bsi*WI and 2141-bp *Bsi*WI-*Avr*II fragments of pBAC/Frag-III to produce pBAC/Frag-III^{KO}. Then, a new *Xba*I run-off site was introduced by site-directed mutagenesis. A DNA fragment was amplified by PCR of pBAC/Frag-IV with primers JSROfw+JSROrv (JSROrv incorporates the antisense sequence of *Xba*I and *Not*I recognition sites in a row). The 283-bp *Sfi*I-*Not*I fragment of the resulting amplicons was ligated with the 16933-bp *Sfi*I-*Not*I fragment of pBAC/Frag-IV to create pBAC/Frag-IV^{RO}.

Thus far, we had constructed a set of four final subclones: pBAC/Frag-I^{SP6}, pBAC/Frag-II, pBAC/Frag-III^{KO}, and pBAC/Frag-IV^{RO}. As summarized in Fig. 1, the full-length SA$_{14}$-14-2 cDNA (designated pBAC/SA$_{14}$-14-2) was assembled by joining the 7456-bp *Not*I-*Pac*I fragment of pBAC^{SP6}/JVFLx/XbaI with the following four fragments in a sequential manner: (*i*) the 2022-bp *Pac*I-*Bsr*GI fragment of pBAC/Frag-I^{SP6}, (*ii*) the 3689-bp *Bsr*GI-*Bam*HI fragment of pBAC/Frag-II, (*iii*) the 3800-bp *Bam*HI-*Ava*I fragment of pBAC/Frag-III^{KO}, and (*iv*) the 1607-bp *Ava*I-*Not*I fragment of pBAC/Frag-IV^{RO}.

Mutagenesis of the full-length infectious SA$_{14}$-14-2 cDNA

Two panels of SA$_{14}$-14-2 mutants were constructed by overlap extension PCR mutagenesis. In all cases, pBAC/SA$_{14}$-14-2 was used as an initial template for PCR.

(*i*) 8 pseudoreversion mutants (G^{317}A, U^{419}C, G^{1708}A, U^{2580}C, C^{3215}U, C^{5987}U, G^{6551}A, and U^{8588}C): For the G^{317}A and U^{419}C mutants, two overlapping fragments were first amplified by PCR with two pairs of primers, JSsp6fw+317-1rv/317-2fw+JSFragIrv and JSsp6fw+419-1rv/419-2fw+JSFragIrv, respectively. In both cases, the two synthesized fragments were fused by a second round of PCR with primers JSsp6fw and JSFragIrv, and the 760-bp *Pac*I-*Bsi*WI fragment of the resulting amplicons was ligated with the 4951-bp *Bsi*WI-*Bam*HI and 12863-bp *Bam*HI-*Pac*I fragments of pBAC/SA$_{14}$-14-2.

For the $G^{1708}A$ mutant, the first two fragments were produced by PCR with the following two primer pairs: 1708-1fw+1708-1rv/1708-2fw+1708-2rv. These two fragments were then fused by a second round of PCR with primers 1708-1fw and 1708-2rv. The 762-bp *Nhe*I-*Bsr*GI fragment of the fused amplicons was ligated with the 9096-bp *Bsr*GI-*Not*I and 8716-bp *Not*I-*Nhe*I fragments of pBAC/SA_{14}-14-2.

For the $U^{2580}C$ and $C^{3215}U$ mutants, two overlapping fragments were first synthesized by PCR with two pairs of primers, namely JSBsrGIfw+2580-1rv/2580-2fw+JSBamHIrv and JSBsrGIfw+3215-1rv/3215-2fw+JSBamHIrv, respectively. In each case, the two fragments were fused by a second round of PCR with the JSBsrGIfw and JSBamHIrv primers. The 3689-bp *Bsr*GI-*Bam*HI fragment of the resulting amplicons was ligated with the 9489-bp *Bam*HI-*Kpn*I and 5396-bp *Kpn*I-*Bsr*GI fragments of pBAC/SA_{14}-14-2.

For the $C^{5987}U$ mutant, the first two fragments were amplified by PCR with the following two primer pairs: JSBamHIfw+5987-1rv/5987-2fw+5987-2rv. These two fragments were fused by a second round of PCR with primers JSBamHIfw and 5987-2rv. The 736-bp *Bam*HI-*Bsi*WI fragment of the resulting amplicons was ligated with the 12127-bp *Bsi*WI-*Pac*I and 5711-bp *Pac*I-*Bam*HI fragments of pBAC/SA_{14}-14-2.

For the $G^{6551}A$ mutant, two overlapping fragments were first generated by PCR with two pairs of primers, JSBamHIfw+6551-1rv/6551-2fw+6551-2rv, and subsequently fused by a second round of PCR with primers JSBamHIfw+6551-2rv. The 1539-bp *Bam*HI-*Nhe*I fragment of the fused amplicons was ligated with the 11324-bp *Nhe*I-*Pac*I and 5711-bp *Pac*I-*Bam*HI fragments of pBAC/SA_{14}-14-2.

For the $U^{8588}C$ mutant, the first two overlapping fragments were produced by PCR with two pairs of primers, 8588-1fw+8588-1rv/8588-2fw+8588-2rv, and then fused by a second round of PCR with primers 8588-1fw+8588-2rv. The 2538-bp *Mlu*I-*Sfi*I fragment of the fused products was ligated with the 13450-bp *Sfi*I-*Bam*HI and 2586-bp *Bam*HI-*Mlu*I fragments of pBAC/SA_{14}-14-2.

(*ii*) 14 E-244 mutants ($G^{244}E$, $G^{244}D$, $G^{244}R$, $G^{244}K$, $G^{244}F$, $G^{244}W$, $G^{244}T$, $G^{244}S$, $G^{244}N$, $G^{244}Q$, $G^{244}L$, $G^{244}P$, $G^{244}A$, and $G^{244}V$): We used the same cloning strategy as described for the construction of the $G^{1708}A$ mutant (now renamed $G^{244}E$). For the other 13 mutants, two fragments were first amplified by PCR with the following two primer pairs: $G^{244}D$, 1708-1fw+DR/DF+1708-2rv; $G^{244}R$, 1708-1fw+RR/RF+1708-2rv; $G^{244}K$, 1708-1fw+KR/KF+1708-2rv; $G^{244}F$, 1708-1fw+FR/FF+1708-2rv; $G^{244}W$, 1708-1fw+WR/WF+1708-2rv; $G^{244}T$, 1708-1fw+TR/TF+1708-2rv; $G^{244}S$, 1708-1fw+SR/SF+1708-2rv; $G^{244}N$, 1708-1fw+NR/NF+1708-2rv; $G^{244}Q$, 1708-1fw+QR/QF+1708-2rv; $G^{244}L$, 1708-1fw+LR/LF+1708-2rv; $G^{244}P$, 1708-1fw+PR/PF+1708-2rv; $G^{244}A$, 1708-1fw+AR/AF+1708-2rv; and $G^{244}V$, 1708-1fw+VR/VF+1708-2rv. In all cases, these two fragments were then fused by a second round of PCR with the 1708-1fw and 1708-2rv primers, and the 762-bp *Nhe*I-*Bsr*GI fragment of the resulting amplicons was ligated with the 9096-bp *Bsr*GI-*Not*I and 8716-bp *Not*I-*Nhe*I fragments of pBAC/SA$_{14}$-14-2.

References

1. Sambrook J, Fritsch EF, Maniatis T (1989) Molecular cloning: a laboratory manual: Cold Spring Harbor Laboratory, Cold Spring Harbor, NY.
2. Choi YJ, Yun SI, Kang SY, Lee YM (2006) Identification of 5' and 3' *cis*-acting elements of the porcine reproductive and respiratory syndrome virus: acquisition of novel 5' AU-rich sequences restored replication of a 5'-proximal 7-nucleotide deletion mutant. J Virol 80: 723-736.
3. Kaplan EL, Meier P (1958) Nonparametric estimation from incomplete observations. J Am Stat Assoc 53: 457-481.

Figure legends

Figure S1. Representative focus/plaque morphologies of SA_{14}-14-2^{MCV}. BHK-21 cells were mock-infected or infected with one of the following three JEVs: SA_{14}-14-2^{MCV}, SA_{14}-14-2, or CNU/LP2 (a virulent strain used as a reference). After infection, cells were overlaid with agarose to examine focus/plaque morphologies. At 4 dpi, cell monolayers were first immunostained with a mouse α-JEV antiserum to visualize the infectious foci, and the same monolayers were then restained with crystal violet to observe the infectious plaques. The average plaque sizes (mean ± SD) were determined by counting 10 representative plaques.

Figure S2. Viral growth properties of SA_{14}-14-2^{MCV} in SH-SY5Y and C6/36 cells. Cells were infected at an MOI of 1 with the molecularly cloned virus (SA_{14}-14-2^{MCV}) rescued from the full-length infectious cDNA or the original parental virus (SA_{14}-14-2) used for cDNA construction. Culture supernatants were collected at the hour postinfection (hpi) indicated, and virus titers were determined by plaque assays on BHK-21 cells.

Figure S3. Virological properties of SA_{14}-14-2^{MCV} in mice. Groups of 3-week-old female ICR mice (n=20 per group) were mock-inoculated or inoculated intracerebrally (IC), intramuscularly (IM), or intraperitoneally (IP) with serial 10-fold dilutions of SA_{14}-14-2^{MCV}, SA_{14}-14-2, or CNU/LP2 (a virulent JEV strain used as a reference). Mice were observed for any JEV-induced clinical signs and death every 12 h for 24 days. Survival curves were plotted by the Kaplan-Meier method [3].

Figure S4. A single point mutation promotes susceptibility to SA_{14}-14-2^{MCV} infection of neurons in the CNS. Groups of 3-week-old female ICR mice (n=15 per group) were mock-infected or infected IC with 10^3 PFU of SA_{14}-14-2^{MCV} (Parent), G^{1708}A, or CNU/LP2 (a virulent JEV strain used as a reference). On the indicated days after infection, five mice were subjected for immunostaining of JEV NS1 antigen in fixed brain slices with an α-NS1 antiserum. Presented are representative slides of

amygdala, cerebral cortex, thalamus, hypothalamus, and brainstem (note that hippocampal slides are shown in Fig. 4E). Arrowheads indicate the NS1-positive cells.

Figure S5. Levels of JEV protein accumulation in BHK-21 cells transfected with 14 E-244 mutant RNAs. BHK-21 cells were mock-transfected or transfected with RNAs transcribed from SA_{14}-14-2^{MCV} (Parent) or each of the 14 E-244 mutant cDNAs as indicated. At 18 hpt, viral protein accumulation was analyzed by immunoblotting of cell lysates with a panel of JEV-specific antisera. In parallel, GAPDH protein was used as a loading and transfer control.

Figure S6. The location of E-244 on the crystal structure of the E ectodomain of JEV SA_{14}-14-2. The E ectodomain of JEV SA_{14}-14-2: DI (colored red), DII (yellow), DIII (blue), and the fusion loop (green). The critical residue Gly at E-244 in the *ij* hairpin adjacent to the fusion loop of the viral E DII is shown. The crystal structure of the E ectodomain of JEV SA_{14}-14-2 was retrieved from the RCSB Protein Data Bank (PDB accession code 3P54).

Figure S7. Amino acid sequence alignment of 154 fully sequenced JEV strains at the conserved *ij* hairpin of viral E glycoprotein. Multiple sequence alignments were performed using the amino acid sequence of 154 fully sequenced JEV genomes, including SA_{14} (red), SA_{14}-14-2 (green), and two other SA_{14}-derived attenuated strains, SA_{14}-2-8 (orange) and SA_{14}-12-1-7 (blue). Note that SA_{14} and SA_{14}-14-2 have been sequenced by three and four independent research groups, respectively. The consensus sequence of the *ij* hairpin and its flanking region is presented on top, and only differences from that sequence are shown. Highlighted are the ~15-aa *ij*-hairpin and the position E-244 in that hairpin.

Table S1. Neurovirulence and neuroinvasiveness of SA_{14}-14-2^{MCV} and its three variants in 3-week-old ICR mice.

Table S2. Neurovirulence of SA_{14}-14-2^{MCV} and its eight mutants in 3-week-old ICR mice.

Table S3. Neurovirulence of SA_{14}-14-2^{MCV} and its 14 E-244 mutants in 3-week-old ICR mice.

Table S4. Oligonucleotides used for ligation, cDNA synthesis, and PCR amplification.

Table S1. Neurovirulence and neuroinvasiveness of SA$_{14}$-14-2^{MCV} and its three variants in 3-week-old ICR mice.

Virus	Inoculum (PFU/mouse)	IC				IM				IP			
		Alive	Dead	Total	LD$_{50}$ (PFU)	Alive	Dead	Total	LD$_{50}$ (PFU)	Alive	Dead	Total	LD$_{50}$ (PFU)
SA$_{14}$-14-2^{MCV}	1.5 x 10^5	7	3	10	>1.5 x 10^5	10	0	10	>1.5 x 10^5	10	0	10	>1.5 x 10^5
	1.5 x 10^4	9	1	10		10	0	10		10	0	10	
	1.5 x 10^3	10	0	10		10	0	10		10	0	10	
	1.5 x 10^2	10	0	10		10	0	10		10	0	10	
	1.5 x 10^1	10	0	10		10	0	10		10	0	10	
	1.5	10	0	10		10	0	10		10	0	10	
SA$_{14}$-14-2^{MCV}/V1	1.5 x 10^5	0	10	10	<1.5	10	0	10	>1.5 x 10^5	10	0	10	>1.5 x 10^5
	1.5 x 10^4	0	10	10		10	0	10		10	0	10	
	1.5 x 10^3	0	10	10		10	0	10		10	0	10	
	1.5 x 10^2	0	10	10		10	0	10		10	0	10	
	1.5 x 10^1	0	10	10		10	0	10		10	0	10	
	1.5	2	8	10		10	0	10		10	0	10	
SA$_{14}$-14-2^{MCV}/V2	1.5 x 10^5	0	10	10	<1.5	10	0	10	>1.5 x 10^5	10	0	10	>1.5 x 10^5
	1.5 x 10^4	0	10	10		10	0	10		10	0	10	
	1.5 x 10^3	0	10	10		10	0	10		10	0	10	
	1.5 x 10^2	0	10	10		10	0	10		10	0	10	
	1.5 x 10^1	0	10	10		10	0	10		10	0	10	
	1.5	0	10	10		10	0	10		10	0	10	
SA$_{14}$-14-2^{MCV}/V3	1.5 x 10^5	0	10	10	<1.5	10	0	10	>1.5 x 10^5	10	0	10	>1.5 x 10^5
	1.5 x 10^4	0	10	10		10	0	10		10	0	10	
	1.5 x 10^3	0	10	10		10	0	10		10	0	10	
	1.5 x 10^2	0	10	10		10	0	10		10	0	10	
	1.5 x 10^1	1	9	10		10	0	10		10	0	10	
	1.5	3	7	10		10	0	10		10	0	10	

Table S2. Neurovirulence of SA$_{14}$-14-2^{MCV} and its eight mutants in 3-week-old ICR mice.

Virus	Inoculum	IC				Virus	Inoculum	IC			
	(PFU/mouse)	Alive	Dead	Total	LD$_{50}$ (PFU)		(PFU/mouse)	Alive	Dead	Total	LD$_{50}$ (PFU)
SA$_{14}$-14-2^{MCV}	1.5×10^5	7	3	10	>1.5×10^5	C^{3215}U	1.5×10^5	9	1	10	>1.5×10^5
(Parent)	1.5×10^4	8	2	10			1.5×10^4	10	0	10	
	1.5×10^3	10	0	10			1.5×10^3	10	0	10	
	1.5×10^2	10	0	10			1.5×10^2	10	0	10	
	1.5×10^1	10	0	10			1.5×10^1	10	0	10	
	1.5	10	0	10			1.5	10	0	10	
G^{317}A	1.5×10^5	8	2	10	>1.5×10^5	C^{5987}U	1.5×10^5	10	0	10	>1.5×10^5
	1.5×10^4	9	1	10			1.5×10^4	10	0	10	
	1.5×10^3	9	1	10			1.5×10^3	10	0	10	
	1.5×10^2	10	0	10			1.5×10^2	10	0	10	
	1.5×10^1	10	0	10			1.5×10^1	10	0	10	
	1.5	10	0	10			1.5	10	0	10	
U^{419}C	1.5×10^5	8	2	10	>1.5×10^5	G^{6551}A	1.5×10^5	10	0	10	>1.5×10^5
	1.5×10^4	9	1	10			1.5×10^4	10	0	10	
	1.5×10^3	10	0	10			1.5×10^3	10	0	10	
	1.5×10^2	10	0	10			1.5×10^2	10	0	10	
	1.5×10^1	10	0	10			1.5×10^1	10	0	10	
	1.5	10	0	10			1.5	10	0	10	
G^{1708}A	1.5×10^5	0	8	8	<1.5	U^{8588}C	1.5×10^5	10	0	10	>1.5×10^5
	1.5×10^4	0	10	10			1.5×10^4	9	1	10	
	1.5×10^3	0	10	10			1.5×10^3	10	0	10	
	1.5×10^2	0	10	10			1.5×10^2	10	0	10	
	1.5×10^1	0	10	10			1.5×10^1	10	0	10	
	1.5	0	10	10			1.5	10	0	10	
U^{2580}C	1.5×10^5	8	2	10	>1.5×10^5						
	1.5×10^4	10	0	10							
	1.5×10^3	10	0	10							
	1.5×10^2	10	0	10							
	1.5×10^1	10	0	10							
	1.5	10	0	10							

Table S3. Neurovirulence of SA_{14}-14-2^{MCV} and its 14 E-244 mutants in 3-week-old ICR mice.

Virus	Inoculum (PFU/mouse)	IC Alive	IC Dead	IC Total	IC LD_{50} (PFU)
SA_{14}-14-2^{MCV} (Parent)	1.5×10^5	8	2	10	$>1.5 \times 10^5$
	1.5×10^4	10	0	10	
	1.5×10^3	10	0	10	
	1.5×10^2	10	0	10	
	1.5×10^1	10	0	10	
	1.5	10	0	10	
$G^{244}E$	1.5×10^5	0	10	10	<1.5
	1.5×10^4	0	10	10	
	1.5×10^3	0	10	10	
	1.5×10^2	0	10	10	
	1.5×10^1	0	10	10	
	1.5	3	7	10	
$G^{244}D$	1.5×10^5	0	10	10	1.5
	1.5×10^4	0	10	10	
	1.5×10^3	0	10	10	
	1.5×10^2	0	10	10	
	1.5×10^1	2	8	10	
	1.5	4	6	10	
$G^{244}R$	1.5×10^5			N.D.	$>1.5 \times 10^4$
	1.5×10^4	10	0	10	
	1.5×10^3	10	0	10	
	1.5×10^2	10	0	10	
	1.5×10^1	10	0	10	
	1.5	10	0	10	
$G^{244}K$	1.5×10^5			N.D.	$>1.5 \times 10^4$
	1.5×10^4	7	3	10	
	1.5×10^3	9	1	10	
	1.5×10^2	10	0	10	
	1.5×10^1	10	0	10	
	1.5	10	0	10	
$G^{244}F$	1.5×10^5			N.D.	$>1.5 \times 10^4$
	1.5×10^4	10	0	10	
	1.5×10^3	10	0	10	
	1.5×10^2	10	0	10	
	1.5×10^1	10	0	10	
	1.5	10	0	10	
$G^{244}W$	1.5×10^5			N.D.	$>1.5 \times 10^4$
	1.5×10^4	10	0	10	
	1.5×10^3	10	0	10	
	1.5×10^2	10	0	10	
	1.5×10^1	10	0	10	
	1.5	10	0	10	
$G^{244}T$	1.5×10^5	0	10	10	<1.5
	1.5×10^4	0	10	10	
	1.5×10^3	0	10	10	
	1.5×10^2	0	10	10	
	1.5×10^1	2	8	10	
	1.5	3	7	10	
$G^{244}S$	1.5×10^5	0	10	10	3.1×10^1
	1.5×10^4	1	9	10	
	1.5×10^3	1	9	10	
	1.5×10^2	3	7	10	
	1.5×10^1	5	5	10	
	1.5	9	1	10	
$G^{244}N$	1.5×10^5	6	4	10	$>1.5 \times 10^5$
	1.5×10^4	9	1	10	
	1.5×10^3	10	0	10	
	1.5×10^2	10	0	10	
	1.5×10^1	10	0	10	
	1.5	10	0	10	
$G^{244}Q$	1.5×10^5	0	10	10	1.5
	1.5×10^4	1	9	10	
	1.5×10^3	2	8	10	
	1.5×10^2	1	9	10	
	1.5×10^1	2	8	10	
	1.5	2	8	10	
$G^{244}L$	1.5×10^5	10	0	10	$>1.5 \times 10^5$
	1.5×10^4	10	0	10	
	1.5×10^3	10	0	10	
	1.5×10^2	10	0	10	
	1.5×10^1	10	0	10	
	1.5	10	0	10	
$G^{244}P$	1.5×10^5	0	10	10	<1.5
	1.5×10^4	0	10	10	
	1.5×10^3	0	10	10	
	1.5×10^2	0	10	10	
	1.5×10^1	0	10	10	
	1.5	0	10	10	
$G^{244}A$	1.5×10^5	1	9	10	5.8×10^3
	1.5×10^4	4	6	10	
	1.5×10^3	6	4	10	
	1.5×10^2	8	2	10	
	1.5×10^1	10	0	10	
	1.5	10	0	10	
$G^{244}V$	1.5×10^5	0	10	10	1.2×10^3
	1.5×10^4	3	7	10	
	1.5×10^3	3	7	10	
	1.5×10^2	4	6	10	
	1.5×10^1	8	2	10	
	1.5	9	1	10	

N.D., Not determined.

Table S4. Oligonucleotides used for ligation, cDNA synthesis, and PCR amplification.

Oligonucleotide	Sequence[a] (5'→3')	Polarity	Oligonucleotide	Sequence[a] (5'→3')	Polarity
JS1rt	TAGGGATCTGGGCGTTTCTGGCAAAT	Antisense	5987-2fw	GCTCAACGGAGGGGTAGAGTAGGCAGAAA	Sense
JS1fw	aatcccgggAGAAGTTTATCTGTGTGAACTT	Sense	5987-2rv	TACCTCGGTGTTGTCCTCCAGTATG	Antisense
JS1rv	attgcggccgcCCACGTCGTTGTGCACGAAGAT	Antisense	6551-1rv	TCAGCCGTTGCAACTAAGTACATGGTGTC	Antisense
JS2rt	TTCTGCCTACTCTGCCCCTCCGTTGA	Antisense	6551-2fw	GACACCATGTACTTAGTTGCAACGGCTGA	Sense
JS2fw	aatcccgggTCAAGCTCAGTGATGTTAACAT	Sense	6551-2rv	AATAATGAGCCAGCTTGTGAGTTAA	Antisense
JS2rv	attgcggccgcGATGGGTTTCCGAGGATGACTC	Antisense	8588-1fw	AAGTCCAGAAGTAGAAGAACAACGC	Sense
JS3rt	ACGGTCTTTCCTTCTGCTGCAGGTCT	Antisense	8588-1rv	CCGTGGTATGTCCAGGTGCGGTATGGATG	Antisense
JS3fw	aatcccgggGAGGATACATTGCTACCAAGGT	Sense	8588-2fw	CATCCATACCGCACCTGGACATACCACGG	Sense
JS3rv	attgcggccgcGTAAGTCAGTTCAATTATGGCT	Antisense	8588-2rv	CTATTGCATCCTAGACGAGGCTTGG	Antisense
JS4rt	AGATCCTGTGTTCTTCCTCACCACCA	Antisense	DF	TGGAATTTGAAGACGCGCACGCCACA	Sense
JS4fw	aatcccgggAGTGGAAGGCTCAGGCGTCCAA	Sense	DR	TGTGGCGTGCGCGTCTTCAAATTCCA	Antisense
JS4rv	attgcggccgcAGATCCTGTGTTCTTCCTCACC	Antisense	AF	TGGAATTTGAAGCCGCGCACGCCACA	Sense
JSsp6fw	catacccgcgtattcccacta	Sense	AR	TGTGGCGTGCGCGGCTTCAAATTCCA	Antisense
JSsp6rv	ACAGATAAACTTCTctatagtgtcccctaaa	Antisense	LF	ATGGAATTTGAACTGGCGCACGCCAC	Sense
JSFragIfw	aggggacactatagAGAAGTTTATCTGTGTG	Sense	LR	GTGGCGTGCGCCAGTTCAAATTCCAT	Antisense
JSFragIrv	TGGATCATTGCCCATGGTAAGCTTA	Antisense	PF	ATGGAATTTGAACCGGCGCACGCCAC	Sense
JSX1fw	CGAATGGATCGCACAGTGTGGAGAG	Sense	PR	GTGGCGTGCGCCGGTTCAAATTCCAT	Antisense
JSX1rv	AAAGCTTCAAACTCAAGATACCGTGCTCC	Antisense	VF	TGGAATTTGAAGTCGCGCACGCCACA	Sense
JSX2fw	GGAGCACGGTATCTTGAGTTTGAAGCTTT	Sense	VR	TGTGGCGTGCGCGACTTCAAATTCCA	Antisense
JSX2rv	cacgtggacgagggcatgcctgcag	Antisense	KF	ATGGAATTTGAAAAGGCGCACGCCAC	Sense
JSROfw	CCAGGAGGACTGGGTTACCAAAGCC	Sense	KR	GTGGCGTGCGCCTTTTCAAATTCCAT	Antisense
JSROrv	agggcggccgctctagAGATCCTGTGTTCTTCCTCACCAC	Antisense	RF	ATGGAATTTGAAAGAGCGCACGCCACA	Sense
317-1rv	TTCATTGCCACACTTTTTTCCACTGCTTT	Antisense	RR	TGTGGCGTGCGCTCTTTCAAATTCCAT	Antisense
317-2fw	AAAGCAGTGGAAAAAAGTGTGGCAATGAA	Sense	FF	ATGGAATTTGAATTCGCGCACGCCACA	Sense
419-1rv	ATGATTGAGCCTTCGTTTCCTCCTCTTTT	Antisense	FR	TGTGGCGTGCGCGAATTCAAATTCCAT	Antisense
419-2fw	AAAAGAGGAGGAAACGAAGGCTCAATCAT	Sense	WF	ATGGAATTTGAATGGGCGCACGCCAC	Sense
1708-1fw	ACATTGGACGTCCGCATGATTAACA	Sense	WR	GTGGCGTGCGCCCATTCAAATTCCAT	Antisense
1708-1rv	TTGTGGCGTGCGCCTCTTCAAATTCCATG	Antisense	SF	ATGGAATTTGAAAGCGCGCACGCCACA	Sense
1708-2fw	CATGGAATTTGAAGAGGCGCACGCCACAA	Sense	SR	TGTGGCGTGCGCGCTTTCAAATTCCAT	Antisense
1708-2rv	GATTTTTCGCGAACGAGAATTTTTC	Antisense	TF	ATGGAATTTGAAACGGCGCACGCCAC	Sense
JSBsrGIfw	CTGGCTCTGAAAGGCACAACCTATG	Sense	TR	GTGGCGTGCGCCGTTTCAAATTCCAT	Antisense
2580-1rv	GGGCGTTTCTGGCAGATATTTATACCTAT	Antisense	NF	ATGGAATTTGAAAACGCGCACGCCACA	Sense
2580-2fw	ATAGGTATAAATATCTGCCAGAAACGCCC	Sense	NR	TGTGGCGTGCGCGTTTTCAAATTCCAT	Antisense
JSBamHIrv	TCATGGATTGGGGCATTTGAGTCAG	Antisense	QF	ATGGAATTTGAACAGGCGCACGCCAC	Sense
3215-1rv	TTTGGTCCGGCTATAGTGTGCGGAATGAT	Antisense	QR	GTGGCGTGCGCCTGTTCAAATTCCAT	Antisense
3215-2fw	ATCATTCCGCACACTATAGCCGGACCAAA	Sense	prMErt	ATTTATACCTATCCACCCAGGCTTCC	Antisense
JSBamHIfw	CTTTATGACAGCGACCCCGCCTGGA	Sense	prMEfw	aatctcgagAGTTGTCATAGCTTGTGCAGG	Sense
5987-1rv	TTTCTGCCTACTCTACCCCTCCGTTGAGC	Antisense	prMErv	attccgcggTGATGTCAATGGCACATCCAG	Antisense

[a] JEV-specific sequences are shown in capital letters.

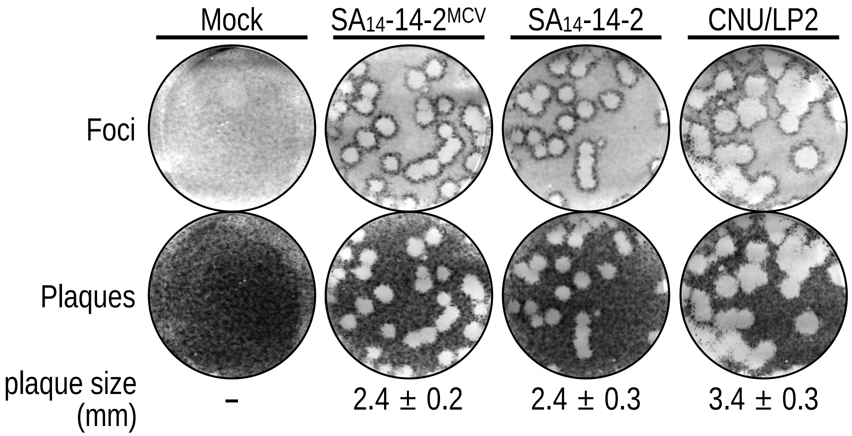
Mock
SA_{14}-14-2^{MCV}
SA_{14}-14-2
CNU/LP2
Foci
Plaques
plaque size (mm)
–
2.4 ± 0.2
2.4 ± 0.3
3.4 ± 0.3

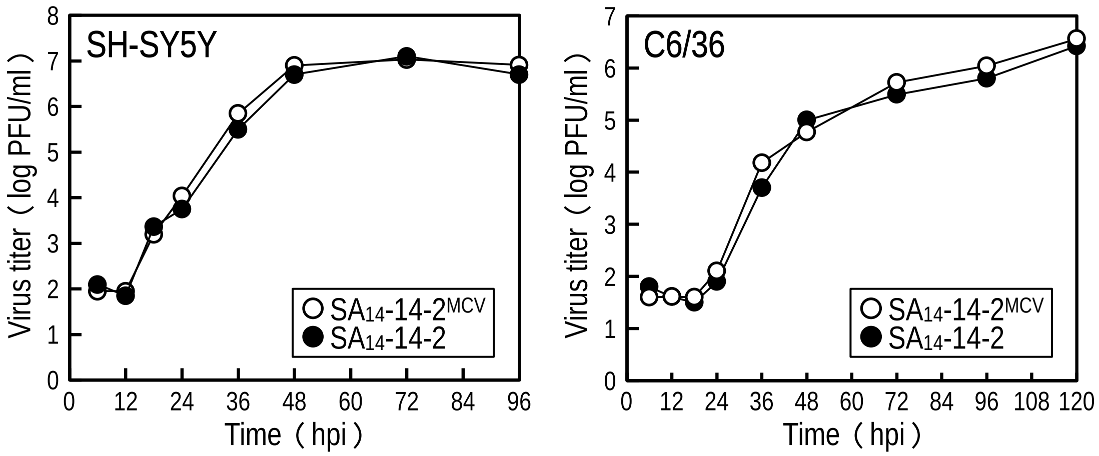
SH-SY5Y
C6/36
Virus titer（log PFU/ml）
Time（hpi）
SA14-14-2MCV
SA14-14-2
0
1
2
3
4
5
6
7
8
0
12
24
36
48
60
72
84
96
108
120

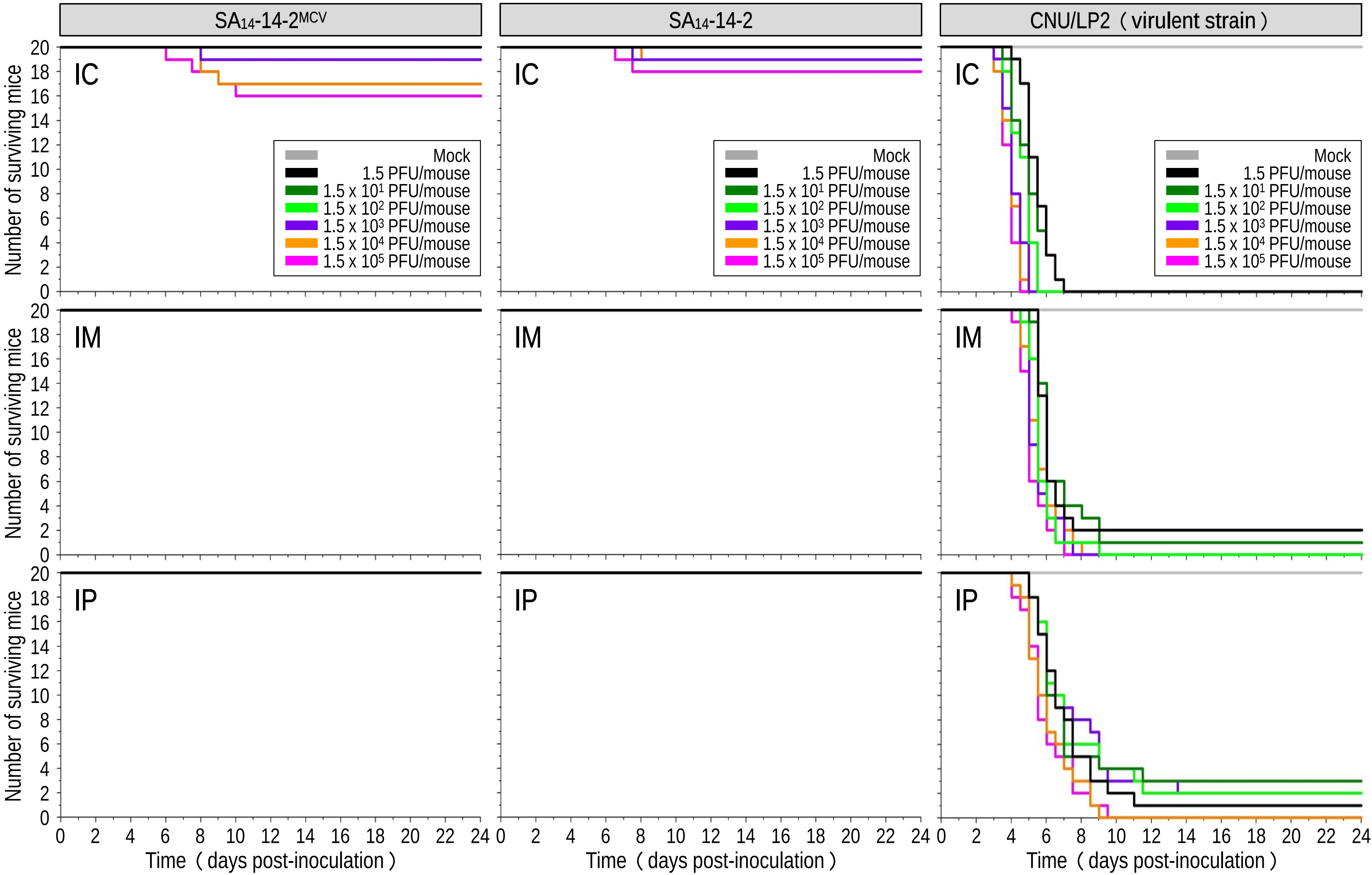

SA14-14-2MCV
SA14-14-2
CNU/LP2（virulent strain）
IC
IM
IP
Number of surviving mice
Time（days post-inoculation）
Mock
1.5 PFU/mouse
1.5 x 10¹ PFU/mouse
1.5 x 10² PFU/mouse
1.5 x 10³ PFU/mouse
1.5 x 10⁴ PFU/mouse
1.5 x 10⁵ PFU/mouse

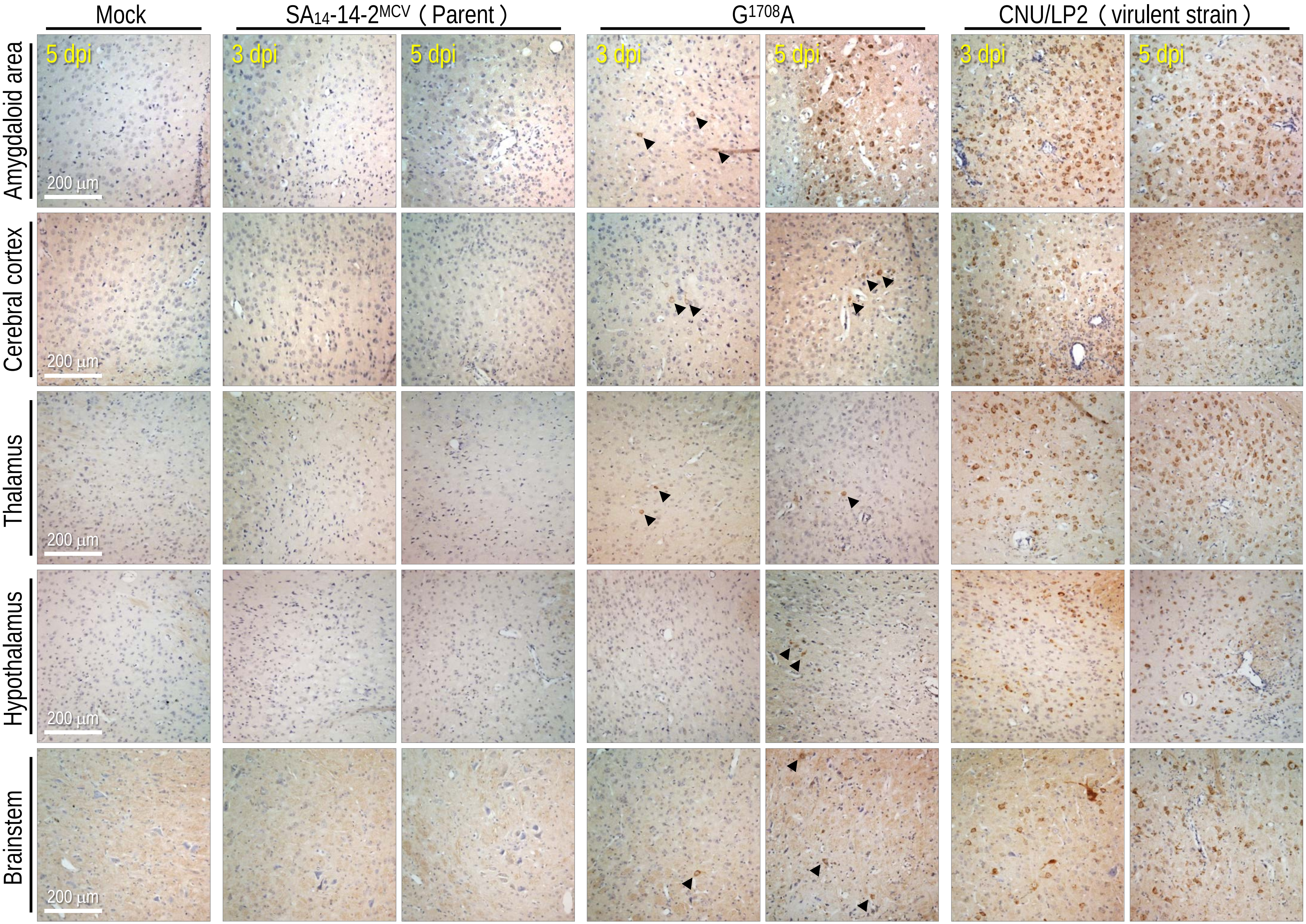
Mock
SA14-14-2MCV (Parent)
G1708A
CNU/LP2 (virulent strain)
Amygdaloid area
Cerebral cortex
Thalamus
Hypothalamus
Brainstem
5 dpi
3 dpi
5 dpi
3 dpi
5 dpi
3 dpi
5 dpi
200 µm

Figure S5. Yun *et al*., 2014

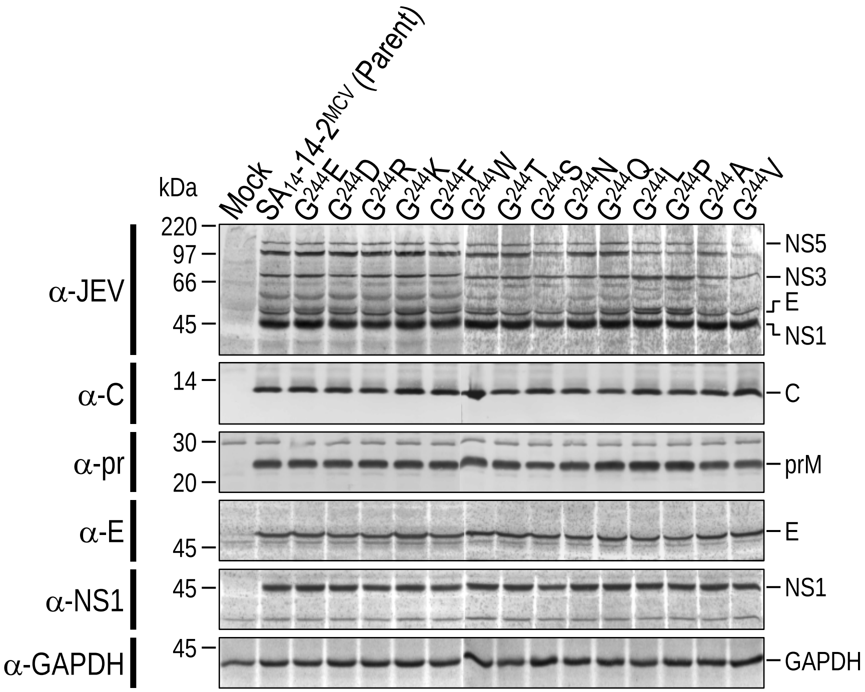

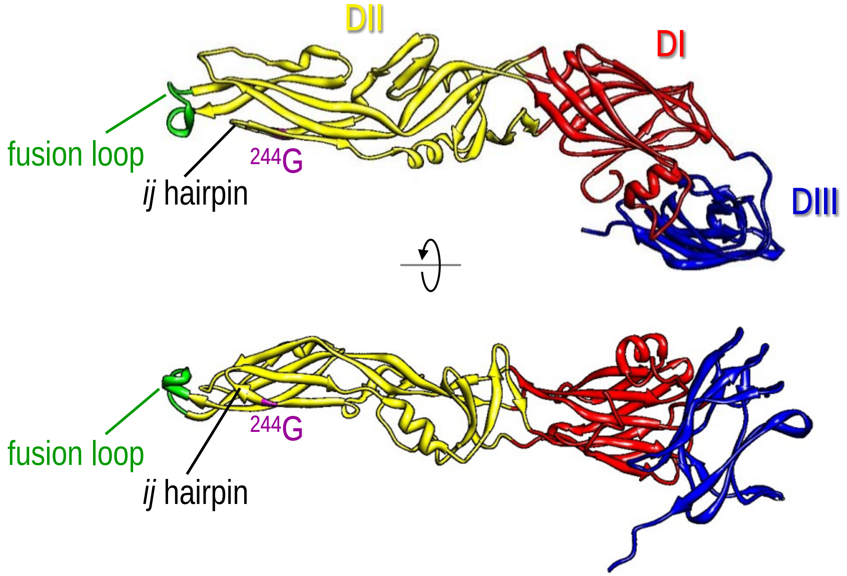
DII
DI
fusion loop
244G
ij hairpin
DIII
fusion loop
244G
ij hairpin

Figure S7. Yun *et al.*, 2014

Strain	228–239	240–254 (ij hairpin)	255–281
Position markers	234 (col. 7)	244 (col. 17)	254, 264, 274
Majority	PSSTAWRNRELL	MEFEEAHATKQSVVA	LGSQEGGLHQALAGAIVVEYSSSVKLT
135 JEV strains[a]	....	....	....
SA_{14} (U14163)	....	....	....
SA_{14} (D90194)	....	....G..........	....
SA_{14} (M55506)	....	....G..........	....
SA_{14}-2-8 (U15763)	....	....G..........	....
SA_{14}-12-1-7 (AF416457)	....	....G..........	.........H.................
SA_{14}-14-2 (JN604986)	....	....G..........	.........H..............M..
SA_{14}-14-2 (D90195)	....	....G..........	.........H..............M..
SA_{14}-14-2 (AF315119)	....	....G..........	.........H..............M..
SA_{14}-14-2 (KC517497)	....	....G..........	....
Sw/GD/01/2009 (KF297915)	....	....G..........	........................M..
K94P05 (AF045551)	....	....Q..........	....
GZ (KC915016)	....T.......	....	....
DH107 (JN381873)	..........F.	....	....
Vellore P20778 (AF080251)	....	....	......S....................
IND-WB-JE1 (JX050179)	....	....	......S....................
Eq/India/H225/2009 (JX131374)	....	....	......S....................
XZ0934 (JF915894)	....N.....I.	....	......A....................
Muar (HM596272)	....N.....I.	L..............	......A....................
XJ69 (EU880214)	....	....	...R.......................
04940-4 (EF623989)	....	....	.........H.................
Nakayama (EF571853)	....	....	.....................N.....
GD (JN711458)	....	....	.....................N.....
HN2 (JN711459)	....	....	.....................N.....
Sw/Mie/51/2006 (AB698905)	....	....	..........................A

ij hairpin

[a] One hundred thirty five full-length genomic sequences are available in GenBank: AB196923, AB196924, AB196925, AB196926, AB241118, AB241119, AB551990, AB551991, AB551992, AB594829, AB698906, AB698907, AB698908, AB698909, AB830335, AB853904, AF069076, AF075723, AF098735, AF098736, AF098737, AF217620, AF221499, AF221500, AF254452, AF254453, AY303791, AY303792, AY303793, AY303794, AY303795, AY303796, AY303797, AY303798, AY316157, AY508812, AY508813, AY585242, AY585243, AY849939, EF107523, EF543861, EF623987, EF623988, EU429297, EU693899, FJ185036, FJ185037, FJ495189, GQ199609, GQ902058, GQ902059, GQ902060, GQ902061, GQ902062, GQ902063, GQ918133, GU187972, GU205163, GU556217, HM228921, HM366552, HQ893545, JF499788, JF499789, JF499790, JF706267, JF706268, JF706269, JF706270, JF706271, JF706272, JF706273, JF706274, JF706275, JF706276, JF706277, JF706278, JF706279, JF706280, JF706281, JF706282, JF706283, JF706284, JF706285, JF706286, JN381830, JN381831, JN381832, JN381833, JN381834, JN381835, JN381836, JN381837, JN381838, JN381839, JN381840, JN381841, JN381842, JN381843, JN381844, JN381845, JN381846, JN381847, JN381848, JN381849, JN381850, JN381851, JN381852, JN381853, JN381854, JN381855, JN381856, JN381857, JN381858, JN381859, JN381860, JN381861, JN381862, JN381863, JN381864, JN381865, JN381866, JN381867, JN381868, JN381869, JN381870, JN381871, JN381872, JQ031753, JX072965, KC196115, L78128, NC_001437, U47032.
